# The Effect of Monodentate Co-Ligands on the Properties of Pt(II) Complexes Bearing a Tridentate C^N*N-Luminophore

**DOI:** 10.3390/molecules28237834

**Published:** 2023-11-29

**Authors:** Stefan Buss, Leon Geerkens, María Victoria Cappellari, Alexander Hepp, Jutta Kösters, Cristian A. Strassert

**Affiliations:** 1 Institut für Anorganische und Analytische Chemie, Universität Münster, Corrensstraße 28/30, 48149 Münster, Germanyl_geer02@uni-muenster.de (L.G.);; 2 CeNTech, CiMIC, SoN, Universität Münster, Heisenbergstraße 11, 48149 Münster, Germany

**Keywords:** soluble triplet emitters, synthesis of Pt(II) complexes, co-ligand exchange, time-resolved photoluminescence spectroscopy, structural characterization, photophysics

## Abstract

In this study, the insertion of different monodentate co-ligands on Pt(II) complexes bearing a monoanionic C^N*N luminophore as a tridentate chelator was achieved beyond the previously reported chlorido- ([**PtCl(L)**]) and cyanido-decorated ([**PtCN(L)**]) analogues. To investigate the impact of the auxiliary ligand on the photophysical properties, we introduced a neutral carbonyl-ligand and observed a lower photoluminescence quantum yield (*Φ*_L_) than with a cyanido moiety. However, the direct substitution of the chlorido co-ligand by a NO-related derivative was not successful. Interestingly, the attempted reduction of the successfully inserted nitrito-*N*-ligand in [**PtNO_2_(L)**] resulted in the oxidation of the Pt(II)-center to Pt(IV), as demonstrated by X-ray diffractometry. For comparison, the trifluoroacetato Pt(II) and chlorido Pt(IV) complexes ([**PtTFA(L)**] and [**PtCl_3_(L)**], respectively) were also synthesized. The photophysical characterization revealed similar photoluminescence profiles for all complexes, indicating a weak effect of the co-ligand on the excited state; in fact, all complexes display emission from metal-perturbed ligand-centered states (even the Pt(IV) species). Nonetheless, longer excited state lifetimes (*τ*_av_) suggest a reduced thermally-activated radiationless deactivation via metal-centered states upon exchange of the chlorido units for other monodentate entities, yet without significantly improving the overall *Φ*_L_ at room temperature. The irreversible oxidation waves (measured via cyclic voltammetry) mostly stem from the Pt(II)-center; the co-ligand-related drop of these potentials correlates with the increasing *σ*-donating capacities of the ancillary ligand. In summary, an enhanced *π*-acceptor capacity does not necessarily improve the *Φ*_L_ and can even impair radiative rates by compromising the perturbative participation of the metal center on the emissive triplet state; in addition, strong *σ*-donor abilities improve the phosphorescence efficiencies by hampering the thermal population of dissociative electronic configurations related to the participation of antibonding d*-orbitals at the metal center.

## 1. Introduction

In the past decades, there has been a notable increase in the attention devoted to triplet-emitting coordination compounds due to their vast range of different applications [1,2,3,4,5]. An exemplary implementation pertains to photocatalysis [1,2,3,6,7]; triplet emitters can also be utilized in OLEDs, where the luminophore is excited electrically via electron and hole recombination events [8,9,10,11,12,13]. Triplet emitters are also valuable in the development of sensing technologies, owing to their manifold quenching pathways [14,15,16,17]. They have also been used in biomedical applications, spanning from multimodal (bio)imaging to photodynamic therapy [18,19,20,21,22,23,24].

Due to their remarkable photophysical performance and adaptability, Pt(II)-based compounds have garnered great attention [6,25,26,27,28,29,30]. The distinct *d*^8^-configuration of Pt(II) results in a square planar coordination geometry where the *d*_z²_-orbitals are available for intermolecular interactions upon aggregation [31,32,33,34].

Advances have been made in the design of potential triplet emitters involving lighter transition metal ions like Pd(II) and Ni(II) [35,36]. However, these species face severe challenges due to the kinetic dominance of radiationless deactivation pathways via thermally accessible metal-centered states (MC). This can be overcome by various strategies [37,38,39,40,41], but the increased ligand field splitting (LFS) from cyanido units is not always sufficient [35,36,42]. Hence, synthetic procedures to incorporate even better co-ligands might lead toward triplet emitters based on more abundant metal centers able to show room-temperature luminescence.

Two prominent classes of monodentate ligands potentially providing a stronger LFS than cyanido units are represented by carbonyl and NO-related species. For instance, Re(I) complexes are known as carbonyl-containing coordination compounds and are currently under investigation for (photo-)biological applications [43]. Furthermore, these co-ligand classes hold an interest in therapeutics, particularly for their properties including (photo-)controlled gas release [44,45,46,47]. CO-releasing complexes are considered for use against neoplastic cells [48,49], whilst the use of NO-releasing agents is also tested for the treatment of cancer and to control blood pressure [50,51,52]. The high spatial selectivity achieved by photo-release (i.e., via phototherapy) of NO or CO molecules is advantageous to reduce the systemic toxicity of bioactive species.

Pt(II) complexes with tri- and tetradentate luminophores are widely utilized for their high rigidity, reducing the potentially dissociative character of metal-centered excited states while slowing down non-radiative processes [53]. A tridentate chelation motif leaves the fourth coordination site free for co-ligand exchange, which can be used to tune the photophysical properties, to introduce functional groups for improved solubility, or for conjugation with other molecules to label, e.g., biological samples [54].

**Figure 1:**
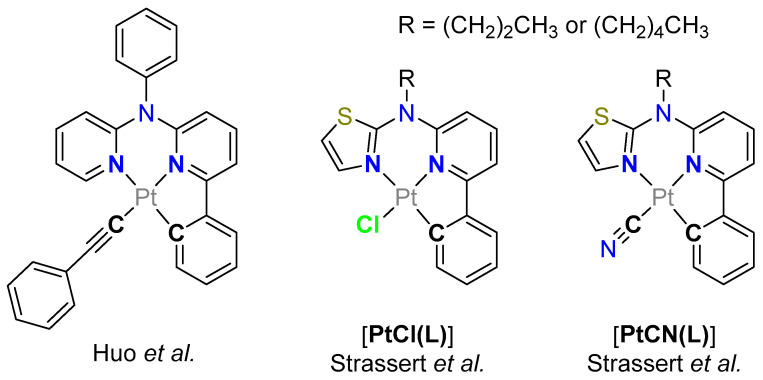
Selected N*N^C-chelated Pt(II) complexes from previous studies. Complexes with a phenylacetylido co-ligand (**left**) [55] as well as the chlorido-[**PtCl(L)**] (**center**) and cyanido-containing analogues [**PtCN(L)**] (**right**) are shown as well [42,56].

Huo et al. have studied the influence of the co-ligand exchange in Pt(II) complexes bearing a C^N*N-luminophore by inserting a phenylacetylido unit (Figure 1) [55]. In our previous work, we reported on a new tridentate C^N*N ligand for functional Pt(II) complexes (allowing to use either a *n*-pentyl-substituted [42] or an *n*-propyl-decorated [56] variation of *N*-(6-phenylpyridin-2-yl)-*N*-alkyl-thiazol-2-amine as the ligand precursor). The photoluminescence quantum yields (*Φ*_L_) and the amplitude-weighted average excited state lifetimes (*τ*_av_) of these compounds are highly dependent on the electronic effects exerted by the co-ligand. This is due to the metal-perturbed ligand-centered (MP-LC) nature of the emissive triplet state, which we have elucidated by comparatively studying analogous chlorido and cyanido complexes ([**PtCl(L)**] and [**PtCN(L)**], respectively) [42,56]. In the work reported herein, we explored the impact of other co-ligands on our C^N*N-based complexes, going from trifluoroacetato (TFA) to carbonyl and oxidizing the Pt(II) complexes to their pseudo-octahedrally coordinated Pt(IV) analogues. We studied the new coordination compounds using steady-state and time-resolved photoluminescence spectroscopy as well as cyclic voltammetry.

## 2. Synthesis and Characterization

The syntheses and photophysical properties of [**PtCl(L)**] and [**PtCN(L)**] were carried out as reported in our previous work [42,56]. The new compounds were characterized by ^1^H-, ^19^F-, ^13^C-, ^195^Pt-, and 2D-nuclear magnetic resonance spectroscopies (NMR, Figures S1–S29) as well as by mass spectrometry (EM-ESI-MS or MALDI-MS).

**Figure 2:**
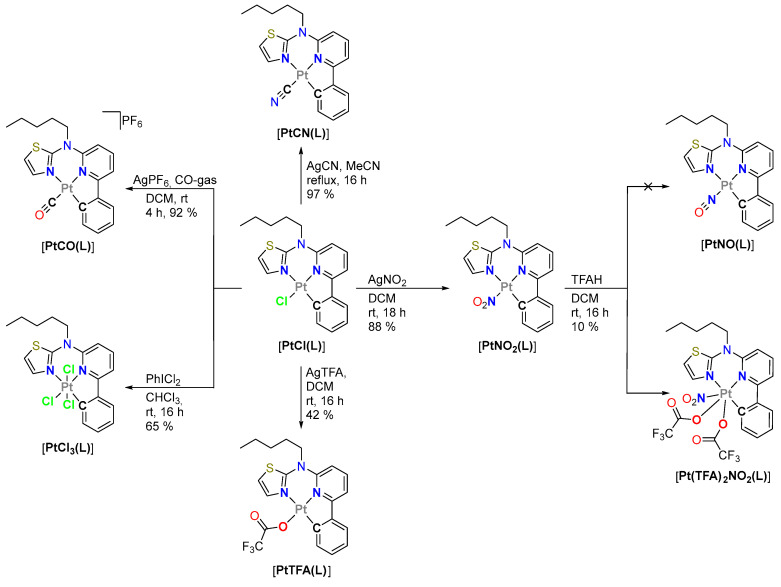
Schematic representation of the co-ligand exchange reactions and the oxidation towards Pt(IV) species.

*Carbonyl complex*: Starting with the product from our established synthetic route towards [**PtCl(L)**], we used this complex as a starting material for the ligand exchange studies [42]. For the introduction of the carbonyl unit, we directly exchanged the ligand by bubbling CO-gas through a dichloromethane (DCM) solution of [**PtCl(L)**] in the absence of oxygen [57]. AgPF_6_ was added from the start to precipitate free chloride anions as insoluble AgCl and to provide a non-coordinating counterion (i.e., PF_6_^−^ to further favor crystallization), which yielded the desired complex [**PtCO(L)**] (charge and counteranion are omitted for clarity in the abbreviation). After multiple days in solution, the complex appears to decompose while leaving a brown solid, which may occur due to unwanted CO-release.

*Nitrito-N complex*: Our methods for the direct exchange of chlorido units towards NO-related species were not successful (NOBF_4_ in DCM; NO-gas in DCM). We also attempted a synthetic route inspired by Slep et al. [58,59], as depicted in Figure 2. They used an acid to reduce a nitrito-*N*-co-ligand on a Ru(II) complex to yield a NO-containing species. Herein, the [**PtNO_2_(L)**] complex was prepared by exchanging the chlorido co-ligand on [**PtCl(L)**] using AgNO_2_. In our attempt to reduce [**PtNO_2_(L)**] to [**PtNO(L)**] with trifluoroacetic acid (TFAH), we obtained a poorly soluble crystalline compound. The X-ray diffractometric analysis revealed the oxidation of the Pt(II) center to Pt(IV) with the coordination of two trifluoroacetato (TFA) co-ligands (*vide infra*) to yield the corresponding complex [**Pt(TFA)_2_NO_2_(L)**]. The poor solubility did not allow for analysis via ^13^C- and 2D-NMR. Although the TFA ligands are orientated axially and equatorially in the solid state, the ^19^F-NMR only shows one main signal. In addition, the yellow crystals slowly turn brown on their surface, indicating that this complex decays in solution and if exposed to air. For comparison with this unexpected product, the analogous trifluoroacetato complex [**PtTFA(L)**] was prepared by using [**PtCl(L)**] and AgTFA in DCM.

*Pt(IV)*: Due to the unexpected oxidation of the Pt(II) center to Pt(IV), we attempted the investigation of the tris-chlorido Pt(IV) complex [**PtCl_3_(L)**] for comparison. The bibliographic literature discusses the oxidation of Pt(II) complexes to their Pt(IV) counterparts in CHCl_3_ under atmospheric conditions [60]. A crystallization attempt of [**PtCl(L)**] in CHCl_3_ led to the formation of single crystals of [**PtCl_3_(L)**] after a month. For a quick preparative approach, hypervalent iodine (PhICl_2_) in CHCl_3_ was used to obtain the [**PtCl_3_(L)**] species in good yields [61]. Further attempts to exchange the chlorido-ligands using AgCN in MeCN under reflux [56] did not yield [**PtCN_3_(L)**] but retained the educt species.

## 3. X-ray Diffractometric Analysis

We were able to obtain the molecular structures of [**PtNO_2_(L)**], [**Pt(TFA)_2_NO_2_(L)**], [**PtTFA(L)**], and [**PtCl_3_(L)**] from single crystals by X-ray diffractometric analysis, as shown in Figure 3 and Figure 4 (further details are found in the SI, Figures S30–S38 and Tables S1–S6).

**Figure 3:**
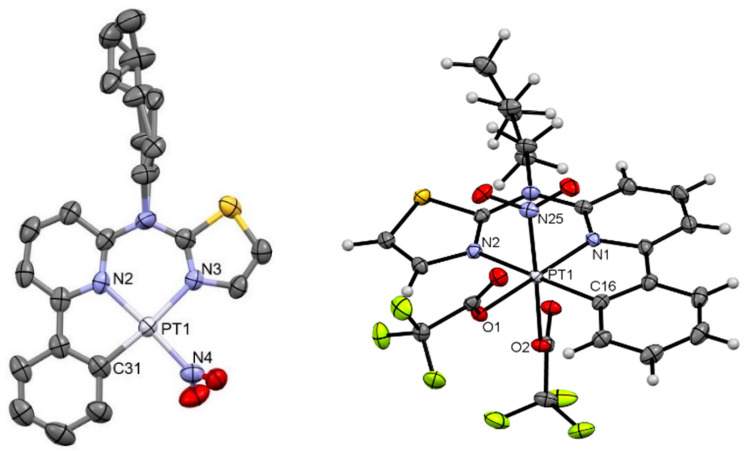
Molecular structure in the crystalline phase of [**PtNO_2_(L)**] (**left**; CCDC-Nr.: 2298207) and [**Pt(TFA)_2_NO_2_(L)**] (**right**; CCDC-Nr.: 2298209). Hydrogen atoms are omitted for clarity (left). Displacement ellipsoids are shown at 50% probability.

*[**PtNO_2_(L)**]*: The molecular structure confirms the chlorido-co-ligand exchange by nitrito-*N* and coordination *via* nitrogen in a slightly distorted square planar configuration (Figure 3, left), with the nitrito unit bent out of the coordination plane by roughly 71° (dihedral angle C31-Pt1-N4-O2). The complex crystallizes in a trigonal system (space group *R*-3), which is isostructural with the precursor complex [**PtCl(L)**] [42]. Not only is the crystal system similar to [**PtCl(L)**] but also the bond lengths and angles are nearly identical. The only exception is the Pt-X (co-ligand) bond, where the value is closer to the [**PtCN(L)**] complex: Pt-N distance 1.999(5) vs. Pt-Cl 2.2982(16) vs. Pt-C 1.948(6). In the case of the chlorido-complex, the alkyl-chain is displaced over two positions. The formation of head-to-tail dimers via *π*–*π*-interactions yields 1D chains (Figure S30). Due to the O–H interaction, the zic-zac shift of dimers in these chains is larger if compared with the Pt-Cl precursor. Other 3D contacts can be described as *van der Waals* interactions.

*[**Pt(TFA)_2_NO_2_(L)**]*: Crystals suitable for X-ray diffractometry precipitated directly from the reaction mixture. The structural assignment reveals an octahedral coordination geometry without counterions in close proximity, in agreement with the proposed oxidation of the metal center towards Pt(IV) while featuring the in-plane tridentate ligand, one axial nitrito-*N*-co-ligand that is shifted out-of-plane upon oxidation, and two TFA-ligands (see Figure 3, right). The complex crystallizes in a monoclinic lattice (space group *P*2_1_/*c*). The bond lengths and angles are similar to those found in the precursor [**PtCl(L)**]. Despite the octahedral coordination environment, the complex forms dimers with H–O interactions between the alkyl-chain and the nitrito-*N*-ligand (Figures S33 and S34). With only one nitrito-*N*-ligand, the interaction is limited to dimers, while the rest of the 3D-packing is dominated by F/O–H interactions from the TFA-ligands.

**Figure 4:**
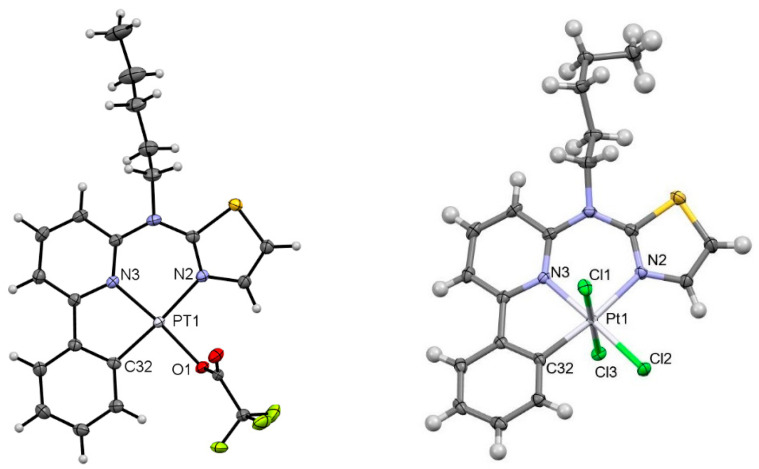
Molecular structure in the crystalline phase of [**PtTFA(L)**] (**left**; CCDC-Nr.: 2298208) and [**PtCl_3_(L)**] (**right**; CCDC-Nr.: 2298210). Displacement ellipsoids are shown at 50% probability.

*[**PtTFA(L)**]*: The diffusion of *n*-hexane into a saturated solution of [**PtTFA(L)**] in DCM led to the formation of rod-like crystals suitable for X-ray crystallography (Figure 4, left). The molecular structure confirms the coordination of TFA *via* oxygen from the carboxylic acid. It crystallizes in a triclinic system (space group *P*-1). As in the case of [**PtNO_2_(L)**], the bond lengths and angles are similar to those found in [**PtCl(L)**] and [**Pt(TFA)_2_NO_2_(L)**]. The complex forms head-to-tail dimers arranged in 1D chains stabilized by *π*–*π* interactions as well as by O–H interactions. The 3D packing is mostly determined by O–H interactions with the neighboring alkyl chains (Figure S36).

*[**PtCl_3_(L)**]*: The botryoidal crystals of [**PtCl_3_(L)**] (Figure 4, right) were obtained by slowly evaporating a saturated solution of [**PtCl(L)**] in CHCl_3_. The oxidation from Pt(II) to Pt(IV) in and with CHCl_3_ is known from the bibliographic literature [60]. As in the case of [**Pt(TFA)_2_NO_2_(L)**], [**PtCl_3_(L)**] crystallizes in a monoclinic system (space group *P*2_1_/*c*). The bond lengths are marginally longer, if compared with their Pt(II) counterparts, namely 2.003(3) vs. 1.967(7) for Pt-C, and 2.3133(7) vs. 2.2982(16) for Pt-Cl. The axial ligands sterically permit *π* interactions, leaving the Cl-H interactions as the main determinants of the 3D arrangement.

## 4. Photophysics

The photophysical properties are summarized in Table 1 and Figure 5 (the complete set of data is detailed in the SI, Table S7, with the spectra and photoluminescence decay plots shown in Figures S39–S53). 

The molar absorption coefficients are depicted in Figure S39. The assignment of the absorption bands can be conducted by comparison with analogous compounds [56,62,63]. The high-energy bands with strong molar absorption coefficients below 325 nm correspond to transitions into states that can be described as ^1^ππ*-configurations (i.e., with ligand-centered character, ^1^LC); thus, the lower energy bands around 350 nm and above can be generally assigned to transitions into states with mixed ligand-centered/metal-to-ligand charge-transfer character (i.e., ^1^LC/^1^MLCT). The energy of the absorption bands remains similar to the reference compound [**PtCl(L)**], and only the relative intensities are somewhat affected by the co-ligand exchange [42,56,62,63].

**Figure 5:**
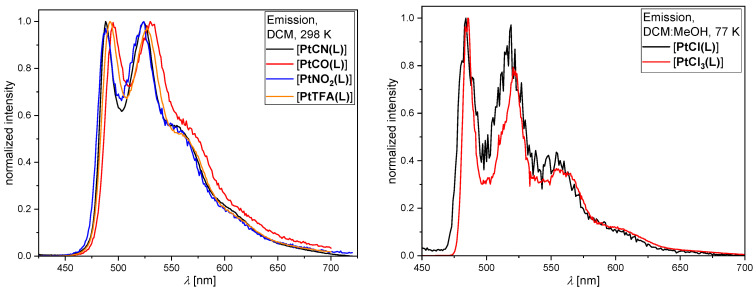
Steady-state photoluminescence spectra at 298 K in liquid DCM at RT (**left**) for [**PtCN(L)**] (black), [**PtCO(L)**] (red), [**PtNO_2_(L)**] (blue), and [**PtTFA(L)**] (orange), as well as at 77 K in frozen glassy matrices of DCM/MeOH (*v*:*v* = 1:1) (**right**) of [**PtCl(L)**] (black) and [**PtCl_3_(L)**] (red). All solutions used for the emission spectra were optically diluted (A < 0.1), and the spectra were normalized to the highest intensity.

At room temperature in liquid DCM, nearly all complexes show a characteristic green photoluminescence peaking at around *λ*_max_ ≈ 490 nm, with an invariant vibrational progression (Figure 5, left); however, [**PtCl_3_(L)**] is not emissive in liquid solutions at room temperature. The triplet character is indicated by the dependency of the *τ*_av_ on the presence of dissolved ^3^O_2_; in fact, all samples show prolonged *τ*_av_ values upon purging the solutions with argon. Compared to [**PtCl(L)**], all *τ*_av_ are longer upon co-ligand exchange, indicating that the non-radiative rate constants are reduced. Despite the ability to tune the radiationless deactivation rates by co-ligand exchange, the relatively low radiative rate constant cannot be enhanced, leading to low *Φ*_L_-values in all cases (except for cyanido; here, the radiationless deactivation is drastically suppressed while allowing a relatively slow yet still competitive phosphorescence). Despite carbonyl being the stronger ligand, [**PtCO(L)**] shows a weaker emission intensity than [**PtCN(L)**]. The strong *π*-acceptor character alone is not sufficient, and the *σ*-donor properties seem to be too weak to properly destabilize antibonding metal-centered d*-orbitals (i.e., in order to avoid the thermal population of dark states). Thus, it is frequently observed that the insertion of phosphane ligands renders the resulting complexes only weakly emissive in liquid solutions at room temperature [64]. The other two co-ligands reported herein provide lifetimes that are longer than in the case of chlorido units but also fail to enhance the room-temperature phosphorescence in solution. At 77 K in frozen glassy matrices, the *τ*_av_ values are further increased (Table S7), which can be explained by considering that the formal population of antibonding metal-centered *d*_x_^2^-_y_^2^ orbitals becomes thermally inaccessible; thus, the non-radiative rate component is slowed down. Due to the loss of solvent stabilization with a concomitantly reduced metal-to-ligand charge-transfer character in frozen glassy matrices, the maxima (*λ*_max_ ≈ 485 nm) appear blue-shifted and display a sharp vibrational progression.

**Table 1:** Selected photophysical data for the complexes in liquid DCM at 298 K. A complete set of data, including values at 77 K, is provided in the SI (see Table S7).

Complex	*λ*_abs_/nm (*ε*/10^3^ M^−1^ cm^−1^)	*λ*_em_/nm	*τ*_av_^a^/μs	*Φ*_L_ ± 0.02
[**PtCl(L)**] [42]	246 (12.5), 266 (20.1), 278 (18.1), 316 (8.1), 348 (8.3), 370 (5.2)	496	0.0191 ± 0.0008	<0.02
[**PtCN(L)**] [42]	257sh (27.7), 268 (29.9), 279sh (27.4), 304 (18.7), 342 (12.7), 358 (13.6), 400 (1.4)	488	35.4 ± 0.3	0.52
[**PtCO(L)**]	263 (19.2), 294 (16.4), 305sh (15.2), 364 (10.7), 388sh (5.7)	495	6.89 ± 0.04	<0.02
[**PtNO_2_(L)**]	263 (28.1), 278sh (21.7), 300 (14.7), 346 (11.0), 357 (10.6), 400sh (1.2)	488	1.044 ± 0.005	<0.02
[**PtTFA(L)**]	244 (20.1), 261 (25.2), 286 (18.1), 307 (12.0), 332sh (6.9), 345 (10.8), 361 (8.6), 411sh (0.7)	493	1.087 ± 0.001	0.03
[**PtCl_3_(L)**]	263 (13.9), 269 (14.5), 298 (13.9), 354 (11.0)	n.d.	n.d.	n.d.

^a^ *λ*_exc_ = 376 nm, reported as amplitude-weighted averaged lifetimes, according to the suggestion from the work of Engelborghs et al. [65] (the single-exponential components, relative amplitudes, and uncertainties are listed in the SI; see Table S7); n.d. means “not determined”.

Overall, the effect of co-ligands on the emission profile is negligible since the excited state character is mostly dominated by the tridentate luminophore with a minor perturbation of the metal center (it does, however, impact the deactivation rates). Even oxidation of the metal center from Pt(II) to Pt(IV) only affects the *τ*_av_, leaving [**PtCl(L)**] and [**PtCl_3_(L)**] with nearly identical emission spectra at 77 K (Figure 5, right). In any case, the exchange of chlorido by other co-ligands prolongs the lifetimes, but only the cyanido species is able to provide complexes with room-temperature phosphorescence in liquid solutions; this can be attributed to its strong *π*-acceptor/*σ*-donor character, as opposed to the *π*-donor/*σ*-donor characteristics of chlorido species.

## 5. Cyclic Voltammetry

The redox potentials of the complexes were determined by cyclic voltammetry in a DCM solution with tetra-*n*-butylammonium hexafluoridophosphate (TBAHFP) (0.1 M) as the supporting electrolyte under an inert atmosphere achieved by purging the solvent with argon. Figures S54–S60 display the cyclic voltammograms (CV) of the [**PtX**(**L**)] complexes, revealing distinct redox profiles for each system, which are either quasi-reversible or irreversible. The irreversible redox processes maintained this character even at different rates, such as 50, 100, 250, 500, and 1000 mVs^−1^.

**Table 2:** Redox potentials of the Pt-based complexes ^a^.

	*E*_ox1_	*E*_ox2_	*E*_red_	*E*_red2_
[**PtCl(L)**]	0.395	0.821	-	-
[**PtCN(L)**]	0.386	-	-	-
[**PtCO(L)**]	0.520	-	−1.260	−1.670
[**PtNO_2_(L)**]	0.808	-	−2.430	-
[**PtTFA(L)**]	0.637	1.133	-	-
[**PtCl_3_(L)**]	-	-	−1.087	-

^a^ From cyclic voltammetry in TBAHFP/DCM. Potentials in V *vs*. ferrocene/ferrocenium. The estimated uncertainties are ≤0.001 V.

The electrochemical data relative to the ferrocene/ferrocenium couple (Fc/Fc^+^) under identical conditions are reported in Table 2. When scanning toward positive potentials, [**PtCO(L)**], [**PtCN(L)**], and [**PtNO_2_(L)**] exhibited irreversible oxidation waves, whereas [**PtCl(L)**] and [**PtTFA(L)**] showed quasi-reversible (yet well-defined) oxidation waves within the range from 0.39 to 0.81 V. The increasing oxidation potentials at the metal center (Pt^II^/Pt^III^) can be attributed to the decreasing *σ*-donating ability of the ancillary ligands [66,67]. It should be noted that distorted-square-planar Pt(III) centers are susceptible to coordination by solvent molecules followed by disproportionation to Pt(II) and Pt(IV) species, resulting in the typically irreversible nature of Pt(II)-based redox processes [68]. Interestingly, in the cases of [**PtCN(L)**], [**PtCl(L)**], and [**PtTFA(L)**], a quasi-reversible redox couple appeared as a secondary oxidation process. We ascribe these additional quasi-reversible couples to be derived from decomposition products generated upon oxidation of the metal center.

On the cathodic side, two distinct features were observed. [**PtCN(L)**], [**PtCl(L)**], and [**PtTFA(L)**] showed no particular signals, suggesting that the reduction processes are shifted beyond the electrochemical window. Conversely, [**PtCO(L)**], [**PtNO_2_(L)**], and [**PtCl_3_(L)**] exhibited irreversible reduction waves, whereas [**PtCl_3_(L)**] exhibited no corresponding oxidation features. The irreversible reduction wave of [**PtNO_2_(L)**] at −2.430 V (located on the edge of the solvent’s window) can be attributed to a nitrito-centered reduction, according to previous reports [69,70]. For [**PtCO(L)**], two smaller yet irreversible reduction peaks were observed at −1.260 V and −1.670 V. In the case of [**PtCl_3_(L)**], one irreversible wave was detected at −1.087 V, which can be assigned to the reduction of the Pt(IV) center [71,72].

For [**Pt(TFA)_2_NO_2_(L)**], we observed a cyclovoltammetric behavior comparable with a Pt(II)-based species (Figure S58); this may result from a decomposition product stemming from this complex.

## 6. Conclusions

In this work, we have reported the synthesis and photophysical characterization of C^N*N-coordinated Pt-based complexes. We performed ligand exchange reactions of the chlorido co-ligand in [**PtCl(L)**] by carbonyl toward [**PtCO(L)**], by nitrito-*N* to form [**PtNO_2_(L)**] and by TFA to yield [**PtTFA(L)**]. Attempts to reduce the nitrito-*N*-complex towards a NO-related derivative resulted in the Pt(IV) entity [**Pt(TFA)_2_NO_2_(L)**]. Additionally, the synthesis of [**PtCl_3_(L)**] was carried out by direct oxidation of [**PtCl(L)**] with PhICl_2_. All the new complexes were characterized by NMR spectroscopy and mass spectrometry. The crystal structures of the new complexes were determined except for [**PtCO(L)**], most likely because of its long-term instability; this might occur due to CO release, which could be the object of further investigation efforts in the context, e.g., of photobiological studies. 

In general, the phosphorescence of all the complexes studied in this work is dominated by metal-perturbed ligand-centered states, resulting in almost invariant emission profiles for all compounds, irrespective of the oxidation state at the metal center and on the particular co-ligands. This leaves the role of the co-ligand to counteract the thermal population of dissociative states, i.e., to slow down non-radiative deactivation processes with the aid of an increased *σ*-donor character. Interestingly, at room temperature, none of these complexes could outperform the cyanido complex [**PtCN(L)**]—even the seemingly competitive carbonyl-analogue is left behind, showing that a high ligand field splitting alone is not sufficient per se; in fact, the strong *σ*-donor character of an anion seems necessary. Thus, the spectrochemical series is insufficient to predict the impact of co-ligands, as an enhanced LFS results both from an increased *σ*-donor capacity (which destabilizes d*-orbitals and corresponding MC-states) as well as from an augmented *π*-accepting character (that eventually reduces metal participation in excited states mediated by *π*-back-bonding with occupied *d_π_*-orbitals, thus impairing efficient spin-orbit coupling, mixing of spin-states, and ultimately reducing the phosphorescence rates). 

Electrochemical studies can reveal the *σ*-donating capacities indirectly via oxidation potentials. As the *σ*-donor ability drops, the oxidation potentials of the species become higher. From the oxidation potentials (see Table 2), we deduce that the cyanido-co-ligand in [**PtCN(L)**] has the strongest *σ*-donor character in the series, which in turn slows down non-radiative deactivation paths to a point where even slow phosphorescence processes are sufficient to reach good quantum yields (52%) with long excited-state lifetimes (35.4 µs). The higher ligand-field splitting character stemming from a carbonyl co-ligand can be associated with stronger *π*-acceptor properties than its cyanido-counterpart; however, the oxidation potentials reveal that it is a weaker *σ*-donor than CN^−^ and even than Cl^−^ anions. The carbonyl-co-ligand therefore is not as potent in blocking non-radiative paths but probably lowers metal participation in emissive ^3^MP-LC states, leading to a relatively short-lived yet weakly emissive triplet state (*Φ*_L_ < 2%).

## 7. Experimental Section

General information about experimental procedures, including instrumental and synthetic methods, structural characterization of the ligand precursors and the complexes, as well as photophysical measurements are provided in the Supporting Information. 

*Materials:* All chemicals were used as purchased from commercially available sources. For the photophysical measurements, spectroscopic-grade solvents (Uvasol^®^) were used.

*Synthesis:* The detailed synthetic procedures and analytical data are provided in the SI. The syntheses of [**PtCl(L)**] and [**PtCN(L)**] are analogous to those yielding the *n*-propyl-substituted complexes reported previously [42,56]. The new compounds were characterized by ^1^H- and ^19^F-, ^13^C-, ^195^Pt-, and 2D-nuclear magnetic resonance spectroscopies (NMR (Figures S1–S29) as well as by mass spectrometry (EM-ESI-MS or MALDI-MS). The metal complexes were further analyzed by (time-resolved) photoluminescence spectroscopy and cyclic voltammetry.

*X-ray diffractometry:* Suitable single crystals for X-ray diffraction measurements were obtained by slowly evaporating the solvent from a saturated DCM/CHCl_3_ solution or by diffusion of cyclohexane or *n*-hexane into such a solution. The full set of data is given in the SI (Tables S1–S6; Figures S30–S38) and have been uploaded to the CCDC database (CCDC-Nr. 2298207–2298210). The molecular structures were graphically processed using the software package Mercury from CCDC [73]. For [**PtNO_3_(L)**] the alert A: PLAT973_ALERT_2_A Check Calcd Positive Resid. Density on Pt1 2.08 eA^−3^ was obtained, which is a known alert for heavy metals and is probably due to difficulties in (or inefficiency in) absorption correction [74].

## Data Availability

CCDC 2298207–2298210 contain the supplementary crystallographic data for this paper. These data can be obtained free of charge via www.ccdc.cam.ac.uk/data_request/cif, or by emailing data_request@ccdc.cam.ac.uk, or by contacting The Cambridge Crystallographic Data Center, 12 Union Road, Cambridge CB2 1EZ, UK; fax: +44-1223-226033.

## Supplementary Materials

The supporting information can be downloaded at: https://www.mdpi.com/article/10.3390/molecules28237834/s1. It contains the detailed synthetic procedures and data, 1D and 2D-NMR spectra of all compounds, details about the X-ray diffractometric measurements and analysis, steady-state and time-resolved photoluminescence spectroscopy set-up, a summary of the spectroscopic results as well as details about the cyclovoltammetry measurements.
