# Supplementary material for: The Effect of Monodentate Co-Ligands on the Properties of Pt(II) Complexes Bearing a Tridentate C^N*N-Luminophore
Source: Molecules. 2023 Nov 29;28(23):7834. doi: 10.3390/molecules28237834 (PMC10708416; doi:10.3390/molecules28237834)
Supplement: Supplementary file 1 [file molecules-28-07834-s001.zip › molecules-2675905-supplementary.pdf]

molecules

MDPI

Supporting Information

The effect of monodentate co-ligands on the properties of Pt(II) complexes bearing a tridentate C^N*N-luminophore

Stefan Buss [1,2,†], Leon Geerkens [1,2,†], María Victoria Cappellari [1,2,†], Alexander Hepp [1], Jutta Kösters [1] and Cristian A. Strassert [1,2,*]

[1] Institut für Anorganische und Analytische Chemie, Universität Münster, Corrensstraße 28/30, 48149 Münster, Germany; l_geer02@uni-muenster.de (L.G.)
[2] CeNTech, CiMIC, SoN, Universität Münster, Heisenbergstraße 11, 48149 Münster, Germany
* Correspondence: ca.s@uni-muenster.de
† These authors contributed equally to this work and share the first authorship.

Contents:

I. Synthesis and structural characterization

I.1 *Synthesis*

I.2 *NMR spectroscopy*

I.3 *X-ray diffractometric analysis*

II. Photophysical characterization

II.1 *Steady-state and time-resolved photoluminescence spectroscopy*

III. Cyclovoltammetry measurements

IV. References

I. Synthesis and structural characterization

I.1 Synthesis

Commercially available reagents were used without further purification. Silica gel 60 (0.063–0.200 mm) for column chromatography was purchased from Merck (mentioned hereafter as silica) and aluminum oxide 90 (standardized for column chromatographic adsorption analysis acc. to Brockmann) from Merck (mentioned hereafter as alumina) were used for column chromatography if not otherwise stated. Chlorido-(κ^3_{CNN}-*N*-(6-phenylidopyridin-2-yl)-*N*-pentyl-thiazol-2-amine)-platinum(II) ([**PtLCl**]) was prepared according to a formerly published procedure [42].

Exact mass (EM) determination by mass spectrometry (MS) was carried out at the Organisch-Chemisches Institut, Univ. Münster, using an LTQ Orbitrap LTQ XL (Thermo-Fisher Scientific, Bremen) with nanospray-injection (ESI) or Autoflex Speed MALDI-TOF with matrix-assisted laser desorption ionization (MALDI).

NMR spectra were obtained on a Bruker Avance I or Avance III 400 (at the Institut für Anorganische und Analytische Chemie, Univ. Münster). All measurements were performed at room temperature (300 K) if not otherwise mentioned. The ^{1}H-NMR, ^{13}C-NMR, ^{19}F-NMR, ^{31}P-NMR, and ^{195}Pt-NMR chemical shifts (δ) of the signals are given in parts per million and referenced to residual protons in the deuterated solvent: DCM-d_2, *i.e.*, methylene chloride-d_2 (5.32 ppm / 53.8 ppm), THF-d_8, *i.e.*, tetrahydrofuran-d_8 (1.72 ppm, 3.58 ppm / 25.3 ppm, 67.2 ppm), MeOD-d_4, *i.e.*, methanol-d_4 (3.31 ppm / 49.0 ppm). The signal multiplicities are abbreviated as follows: s, singlet; d, doublet; t, triplet; q, quartet; m, multiplet.

Preparation of carbonyl-(κ^3_{CNN}-N-(6-phenylidopyridin-2-yl)-N-pentyl-thiazol-2-amine)-platinum(II) (hexafluoridophosphate) [PtCO(L)]

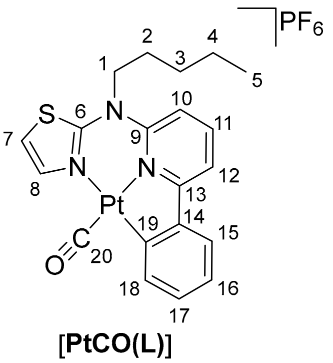

[PtCO(L)]

A solution of [**PtClL**] (77 mg; 0.14 mmol, 1.0 eq) and $AgPF_6$ (35 mg; 0.14 mmol; 1.0 Eq) $AgPF_6$ in 15 mL of DCM was purged with argon with AgCl precipitation. Carbon monoxide was bubbled directly into the solution using a syringe connected to a CO-generator that was purged with argon as well. The carbon monoxide was generated by the dropwise addition of formic acid into concentrated sulfuric acid while vigorously stirring in a large flask cooled with an ice bath. After keeping a steady, light flow of CO-gas through the reaction solution for 4 h, the mixture was directly transferred onto a short-loaded flash chromatography column for purification (silica, eluent: DCM to retrieve residual precursor [**PtClL**], then 2.5% MeOH in DCM *v:v*). The product was obtained as a dark-yellowish, ocher solid (70 mg; 0.13 mmol; 92 %).

Analytical data for [PtCO(L)]:

^{1}H-NMR (400 MHz, DCM-d_2/MeOD-d_4): δ (ppm) = 8.22 (t, $^3J_{HH}$ = 8.3 Hz, 1H, H_{11}), 7.77 (d, $^3J_{HH}$ = 7.8 Hz, 1H, H_{12}), 7.68 (dd, $^3J_{HH}$ = 8.0 Hz, $^4J_{HH}$ = 1.4 Hz, 1H, H_{15}), 7.65 (d, $^3J_{HH}$ = 4.1 Hz, 1H, H_8), 7.47 (d, $^3J_{HH}$ = 8.7 Hz, 1H, H_{10}), 7.43 (d, $^3J_{HH}$ = 4.1 Hz, 1H, H_7), 7.36 (dd, $^3J_{HH}$ = 7.5 Hz, $^4J_{HH}$ = 1.3 Hz, 1H, H_{18}), 7.28 (td, $^3J_{HH}$ = 7.6 Hz, $^3J_{HH}$ = 1.3 Hz, 1H, H_{16}), 7.19 (td, $^3J_{HH}$ = 7.5 Hz, $^4J_{HH}$ = 1.5 Hz, 1H, H_{17}), 4.37 – 4.02 (m, 2H, H_1), 2.02 – 1.88 (m, 2H, H_2), 1.52 – 1.31 (m, 4H, H_{3+4}), 1.01 – 0.82 (m, 3H, H_5).

^{1}H-NMR (500 MHz, DCM-d_2/MeOD-d_4): δ (ppm) = 8.16 (t, $^3J_{HH}$ = 8.2 Hz, 1H, H_{11}), 7.73 – 7.68 (m, 1H, H_{12}), 7.66 – 7.56 (m, 2H, H_{8+15}), 7.45 – 7.39 (m, 2H, H_{7+10}), 7.29 – 7.22 (m, 2H, H_{16+18}), 7.14 (t, $^3J_{HH}$ = 7.8 Hz, 1H, H_{17}), 4.26 – 4.09 (m, 2H, H_1), 1.99 – 1.87 (m, 2H, H_2), 1.59 – 1.33 (m, 4H, H_{3+4}), 1.01 – 0.87 (m, 3H, H_5). (Measured at a later date for 2D reference, already partly decomposed)

^{13}C{^{1}H}-NMR (101 MHz, DCM-d_2/MeOD-d_4): δ (ppm) = 165.2 (C_{20}), 164.9 (C_{13}), 163.0 (C_6), 148.3 (C_9), 143.8 (C_{11}), 143.1 (C_{14}), 142.8 (C_8), 138.0 (C_{19}), 136.4 (C_{18}), 132.4 (C_{17}), 127.0 (C_{16}), 125.7 (C_{15}), 115.4 (C_{12}), 115.3 (C_{10}), 113.0 (C_7), 58.4 (C_1), 28.4 (C_3), 26.0 (C_2), 22.2 (C_4), 13.6 (C_5).

^{19}F-NMR (471 MHz, DCM-d_2/MeOD-d_4): δ (ppm) = -73.5 (d, $^1J_{FP}$ = 710 Hz).

^{31}P-NMR (202 MHz, DCM-*d*$_2$/MeOD-*d*$_4$): *δ* (ppm) = -144.5 (hept, $^1J_{PF}$ = 710 Hz).

^{195}Pt-NMR (107 MHz, DCM-*d*$_2$/MeOD-*d*$_4$): *δ* (ppm) = -3891.

MS-ESI-EM (MeOH/DCM, M = $C_{20}H_{20}N_3SOPt$): found 545.09560 for [M]$^+$ (calcd. *m/z* = 545.09709 for [M]$^+$).

Preparation of nitrito-N-(κ$^3_{CNN}$-N-(6-phenylidopyridin-2-yl)-N-pentyl-thiazol-2-amine)-platinum(II) [PtNO$_2$(L)]

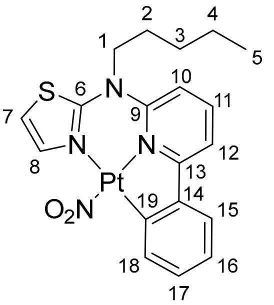

[PtNO$_2$(L)]

[**PtClL**] (150 mg; 0.271 mmol; 1.0 eq) was dissolved in 20 mL of DCM and the solution was purged with argon and wrapped to protect it from light. $AgNO_2$ was recrystallized from hot water and dried *in vacuo* before use. To the reaction solution, $AgNO_2$ (42.0 mg; 0.27 mmol, 1.0 eq) was added and suspended by fast stirring. The mixture was stirred for 18 h at room temperature and directly transferred onto a short-loaded flash chromatography column for purification (silica, eluent: DCM to retrieve residual precursor **1**, then 1% MeOH in DCM *v:v*). [**PtNO$_2$(L)**] was obtained as a pale-yellow solid (134 mg; 0.238 mmol; 88 % yield).

Analytical data for [PtNO$_2$(L)]:

^{1}H-NMR (500 MHz, DCM-*d*$_2$): *δ* (ppm) = 7.97 (dd, $^3J_{HH}$ = 8.6 Hz, $^3J_{HH}$ = 7.8 Hz, 1H, H_{11}), 7.57 – 7.54 (m, 2H, H_{12+15}), 7.42 (d, $^3J_{HH}$ = 4.1 Hz, 1H, H_8), 7.24 – 7.18 (m, 2H, H_{17+18}), 7.18 – 7.13 (m, 2H, H_{10+16}), 7.04 (d, $^3J_{HH}$ = 4.1 Hz, 1H, H_7), 4.22 – 4.09 (m, 2H, H_1), 1.94 (q, $^3J_{HH}$ = 7.3 Hz, 2H, H_2), 1.46 – 1.37 (m, 4H, H_{3+4}), 1.08 – 0.91 (m, 3H, H_5).

^{13}C{^{1}H}-NMR (126 MHz, DCM-*d*$_2$): *δ* (ppm) = 166.8 (C_{13}), 161.6 (C_6), 149.4 (C_9), 144.2 (C_{19}), 144.1 (C_{14}), 140.0 (C_8), 140.0 (C_{11}), 135.0 (C_{18}), 130.8 (C_{17}), 124.6 (C_{16}), 124.1 (C_{15}), 114.3 (C_{12}), 112.6 (C_7), 111.7 (C_{10}), 58.1 (C_1), 28.9 (C_3), 26.5 (C_2), 22.5 (C_4), 14.1 (C_5).

^{195}Pt-NMR (107 MHz, DCM-*d*$_2$): *δ* (ppm) = -3720.

MS-ESI-EM (MeOH, M = $C_{19}H_{20}N_4SO_2Pt$): found 564.10247 for [M+H]$^+$ (calcd. *m/z* = 564.10288 for [M+H]$^+$), found 586.08421 for [M+Na]$^+$ (calcd. *m/z* = 586.08483 for [M+Na]$^+$).

Preparation of Nitrito-N-ditrifluoroacetate-(κ$^3_{CNN}$-N-(6-phenylidopyridin-2-yl)-N-pentyl-thiazol-2-amine)-platinum(IV) [PtNO$_2$(TFA)$_2$(L)]

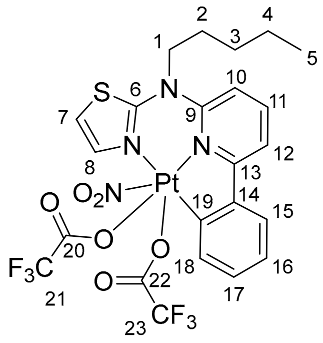

[PtNO$_2$(TFA)$_2$(L)]

[**PtNO$_2$(L)**] (59 mg; 0.10 mmol; 1.0 eq) was dissolved in 10 mL of DCM and 0.1 mL of TFA was added. The mixture was stirred at room temperature overnight and changed colour from pale-yellow to dark-red to brown. The solvent was allowed to evaporate slowly over a week, which led to the formation of crystals that were washed 3 times with 4 mL of DCM and diethylether each. Eight milligrams of [**PtNO$_2$(TFA)$_2$(L)**] (0.010 mmol, 10%) were yielded while the non-reacted [**PtNO$_2$(L)**] could be regained from the washing solutions quantitatively (52 mg, 88%).

Analytical data for [PtNO$_2$(TFA)$_2$(L)]:

^{1}H-NMR (400 MHz, THF-*d*$_8$): *δ* (ppm) = 8.57 (d, *J* = 4.1 Hz, 1H, H_8), 8.09 (d, *J* = 7.9 Hz, 1H, H_{18}), 7.99 (t, *J* = 8.2 Hz, 1H, H_{11}), 7.59 (d, *J* = 7.8 Hz, 1H, H_{12}), 7.49 (d, *J* = 8.0 Hz, 1H, H, H_{15}), 7.26 (d, *J* = 8.6 Hz, 1H, H_{10}), 7.16 (d, *J* = 4.1 Hz, 1H, H_7), 7.07 (t, *J* = 7.4 Hz, 1H, $H_{16/17}$), 6.97 (t, *J* = 7.6 Hz, 1H, $H_{16/17}$), 4.34 – 4.11 (m, 2H, H_1), 2.01 – 1.90 (m, 2H, H_2), 1.48 – 1.32 (m, 4H, H_{3+4}), 1.02 – 0.88 (m, 3H, H_5).

^{19}F-NMR (376 MHz, THF-*d*$_8$): *δ* (ppm) = -76.9.

MS-ESI-EM (MeOH/DCM, M = $C_{23}H_{20}N_4SO_4F_6PtP$): found 812.05624 for [M+Na]$^+$ (calcd. *m/z* = 812.05479 for [M+Na]$^+$), found 743.07312 for [M-NO$_2$]$^+$ (calcd. *m/z* = 743.07266 for [M-NO$_2$]$^+$), found 676.08062 for [M-TFA]$^+$ (calcd. *m/z* = 676.08053 for [M-TFA]$^+$).

Preparation of trifluoroacetate-(κ$^3_{CNN}$-N-(6-phenylidopyridin-2-yl)-N-pentyl-thiazol-2-amine)-platinum(II) [PtTFA(L)]

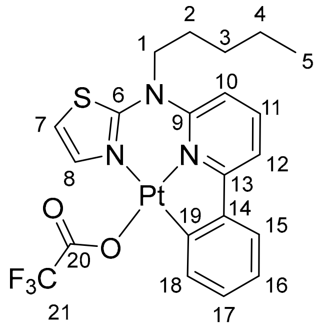

[**PtClL**] (68.0 mg; 0.126 mmol; 1.0 eq.) and AgTFA (56.0 mg; 0.252 mmol; 2.0 eq.) were suspended in DCM. The mixture was purged with argon for 10 min before stirring for 16 h. The suspension was filtrated via column chromatography (DCM); the first pale-yellow fractions yield the product as a yellow to pale-yellow solid (33.0 mg; 0.053 mmol; 42 %).

Analytical data for [PtTFA(L)]:

^{1}H-NMR (500 MHz, DCM-*d*$_2$): δ (ppm) = 7.83 (dd, $^3J_{HH}$ = 8.7 Hz, $^3J_{HH}$ = 7.7 Hz, 1H, H$_{11}$), 7.44 (dd, $^3J_{HH}$ = 7.8 Hz, $^4J_{HH}$ = 1.4 Hz, 1H, H$_{15}$), 7.37 – 7.34 (m, 1H, H$_{12}$), 7.32 (d, $^3J_{HH}$ = 4.1 Hz, 1H, H$_8$), 7.21 (td, $^3J_{HH}$ = 7.3 Hz, $^4J_{HH}$ = 1.4 Hz, 1H, H$_{17}$), 7.15 – 7.08 (m, 2H, H$_{16+18}$), 7.03 (dd, $^3J_{HH}$ = 8.7 Hz, $^4J_{HH}$ = 1.1 Hz, 1H, H$_{10}$), 6.96 (d, $^3J_{HH}$ = 4.0 Hz, 1H, H$_7$), 4.15 – 3.90 (m, 2H, H$_1$), 1.95 – 1.81 (m, 2H, H$_2$), 1.44 – 1.36 (m, 4H, H$_{3+4}$), 1.08 – 0.93 (m, 3H, H$_5$).

^{13}C{^{1}H}-NMR (126 MHz, DCM-*d*$_2$): δ (ppm) = 167.5 (C$_{13}$), 162.4 (q, $^2J_{CF}$ = 36.3 Hz, C$_{20}$), 160.1 (C$_6$), 148.7 (C$_9$), 144.7 (C$_{14/19}$), 144.7 (C$_{14/19}$), 138.8 (C$_{11}$), 136.9 (C$_8$), 132.3 (C$_{18}$), 129.8 (C$_{17}$), 124.2 (C$_{16}$), 123.7 (C$_{15}$), 116.0 (q, $^1J_{CF}$ = 290.1 Hz, C$_{21}$), 113.9 (C$_{12}$), 111.9 (C$_7$), 111.8 (C$_{10}$), 58.1 (C$_1$), 28.8 (C$_3$), 26.0 (C$_2$), 22.5 (C$_4$), 14.2 (C$_5$).

^{19}F-NMR (471 MHz, DCM-*d*$_2$): δ (ppm) = -74.7.

^{195}Pt-NMR (107 MHz, DCM-*d*$_2$): δ (ppm) = -3020.

MS-ESI-EM (MeOH, M = $C_{21}H_{20}N_3SO_2F_3Pt$): found 631.09546 for [M+H]$^+$ (calcd. *m*/*z* = 631.09505 for [M+H]$^+$), found 653.07696 for [M+Na]$^+$ (calcd. *m*/*z* = 653.07700 for [M+Na]$^+$).

Preparation of trichlorido-(κ^3_{CNN}-N-(6-phenylidopyridin-2-yl)-N-pentyl-thiazol-2-amine)-platinum(IV) [PtCl$_3$(L)]

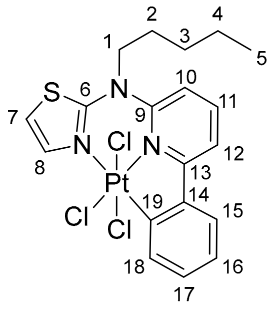

[**PtCl(L)**] (71.0 mg; 0.135 mmol; 1.0 eq.) and PhICl$_2$ (44.5 mg; 0.162 mmol; 1.2 eq.) were suspended in CHCl$_3$ (30 mL) and stirred for 16 h in the dark. The solvent was removed and the residue was filtrated via column chromatography (DCM). The product was obtained as a yellow solid (54.5 mg; 0.087 mmol; 65 %).

Analytical data for [PtCl$_3$(L)]:

^{1}H-NMR (500 MHz, DCM-*d*$_2$): δ (ppm) = 8.45 (d, $^3J_{HH}$ = 4.1 Hz, 1H, H$_8$), 8.07 (dd, $^3J_{HH}$ = 8.5 Hz, $^3J_{HH}$ = 7.8 Hz, 1H, H$_{11}$), 7.98 (dd, $^3J_{HH}$ = 8.1 Hz, $^4J_{HH}$ = 1.2 Hz, 1H, H$_{18}$), 7.83 (dd, $^3J_{HH}$ = 7.9 Hz, $^4J_{HH}$ = 1.2 Hz, 1H, H$_{12}$), 7.73 (dd, $^3J_{HH}$ = 7.8 Hz, $^4J_{HH}$ = 1.6 Hz, 1H, H$_{15}$), 7.43 (ddd, $^3J_{HH}$ = 8.0 Hz, $^3J_{HH}$ = 7.3 Hz, $^4J_{HH}$ = 1.5 Hz, 1H, H$_{17}$), 7.35 – 7.23 (m, 3H, H$_{7+10+16}$), 4.41 – 4.11 (m, 2H, H$_1$), 2.03 – 1.90 (m, 2H, H$_2$), 1.46 – 1.32 (m, 4H, H$_{3+4}$), 0.91 (t, $^3J_{HH}$ = 7.2 Hz, 3H, H$_5$).

^{13}C{^{1}H}-NMR (126 MHz, DCM-*d*$_2$): δ (ppm) = 164.6 (C$_{13}$), 162.7 (C$_6$), 149.1 (C$_9$), 142.4 (C$_{11}$), 141.8 (C$_{14}$), 138.2 (C$_8$), 136.6 (C$_{19}$), 132.8 (C$_{18}$), 132.4 (C$_{17}$), 126.8 (C$_{16}$), 125.8 (C$_{15}$), 116.4 (C$_{12}$), 114.4 (C$_{10}$), 113.9 (C$_7$), 58.6 (C$_1$), 28.9 (C$_3$), 27.4 (C$_2$), 22.5 (C$_4$), 14.0 (C$_5$).

^{195}Pt-NMR (107 MHz, DCM-*d*$_2$): δ (ppm) = -1107.

MS-ESI-EM (MeOH/CHCl$_3$, M = $C_{19}H_{20}N_3SPtCl_3$): found 587.03967 for [M–Cl]$^+$ (calcd. *m*/*z* = 587.03986 for [M–Cl]$^+$), found 641.04053 for [M+NH$_4$]$^+$ (calcd. *m*/*z* = 641.04083 for [M+NH$_4$]$^+$), found 645.99584 for [M+Na]$^+$ (calcd. *m*/*z* = 645.99622 for [M+Na]$^+$).

I.2 NMR spectroscopy

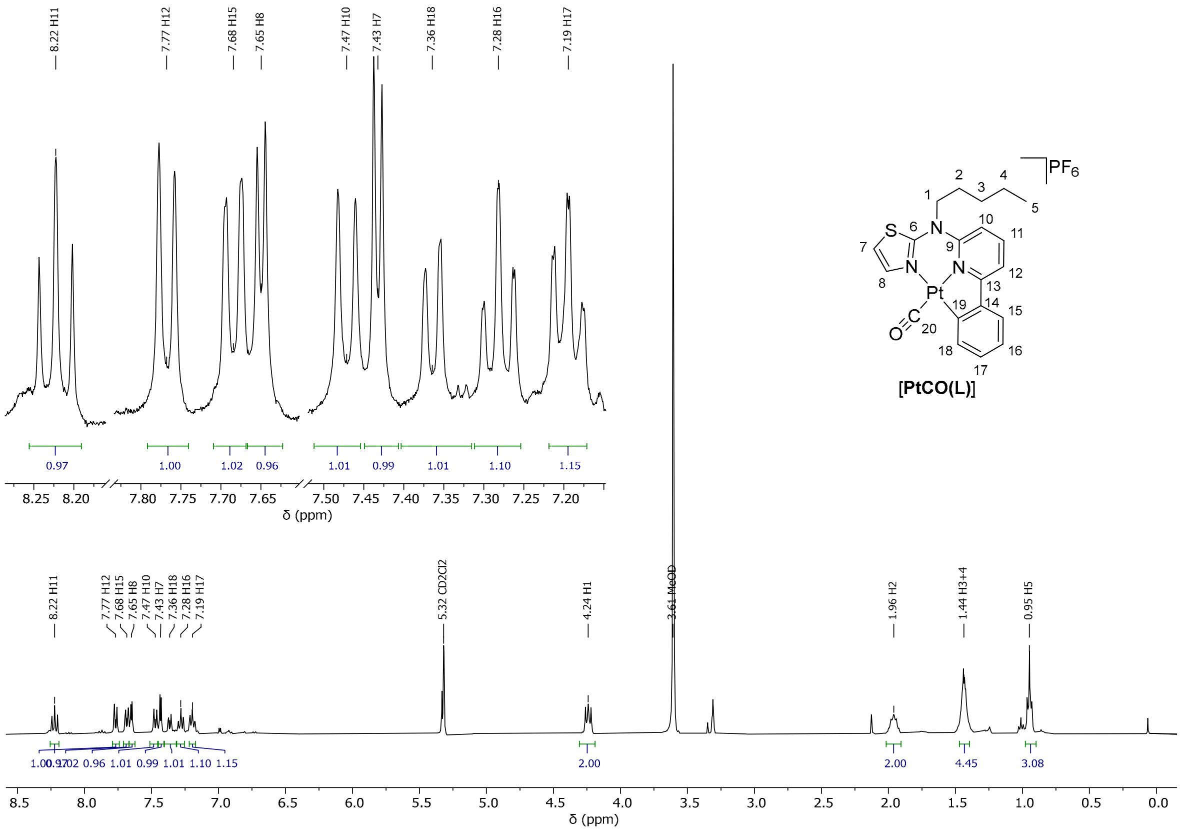

Figure S1. ^{1}H-NMR spectrum (400 MHz, DCM-d$_2$/MeOD-d$_4$) of [**PtCO(L)**].

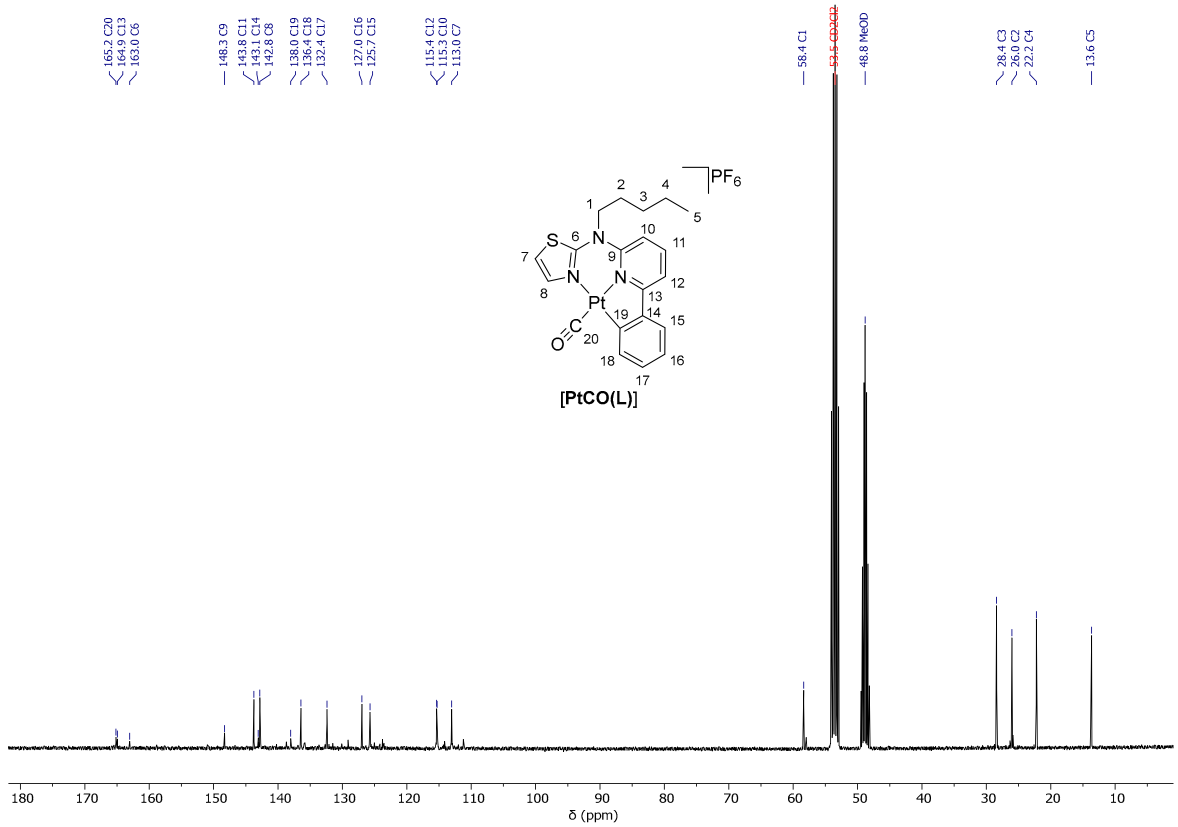

Figure S2. ^{13}C{^{1}H}-NMR spectrum (101 MHz, DCM-d$_2$/MeOD-d$_4$) of [**PtCO(L)**].

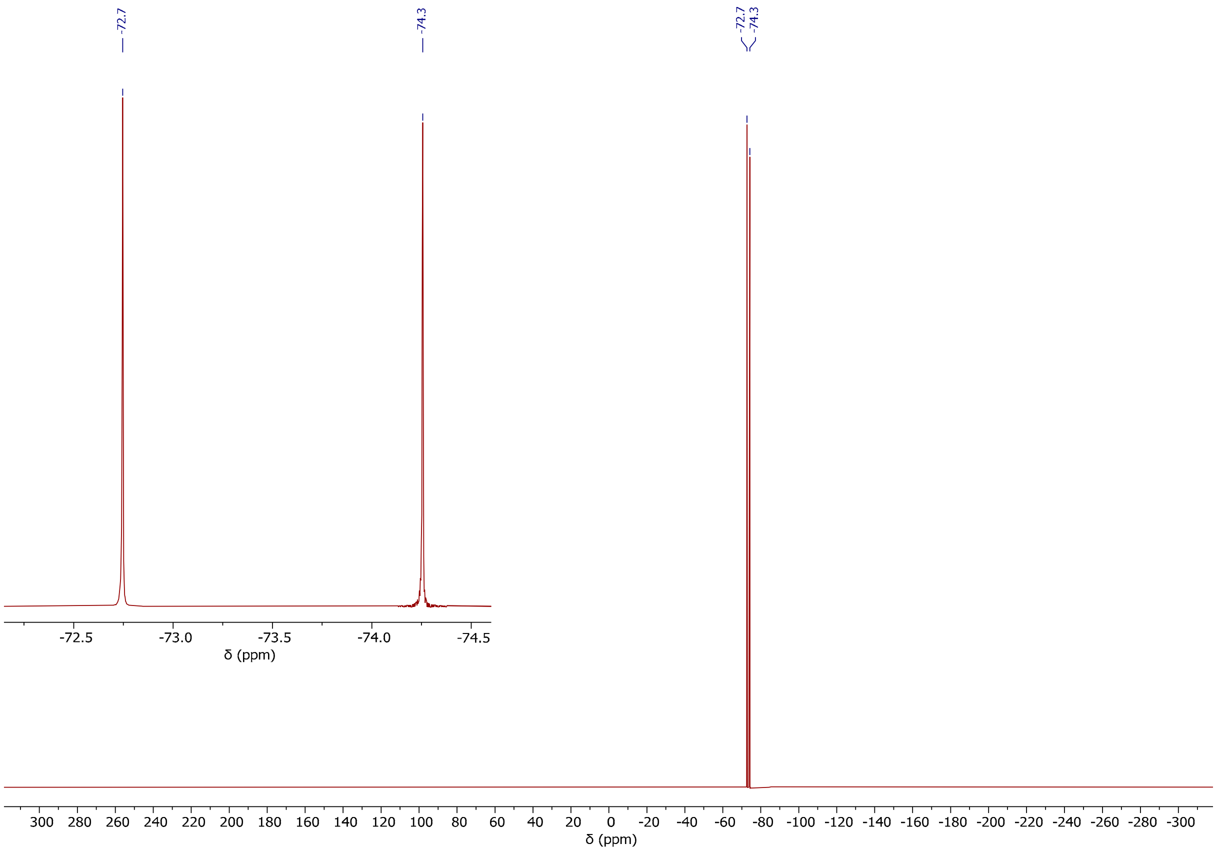

Figure S3. ^{19}F-NMR spectrum (471 MHz, DCM-d_2/MeOD-d_4) of [**PtCO(L)**].

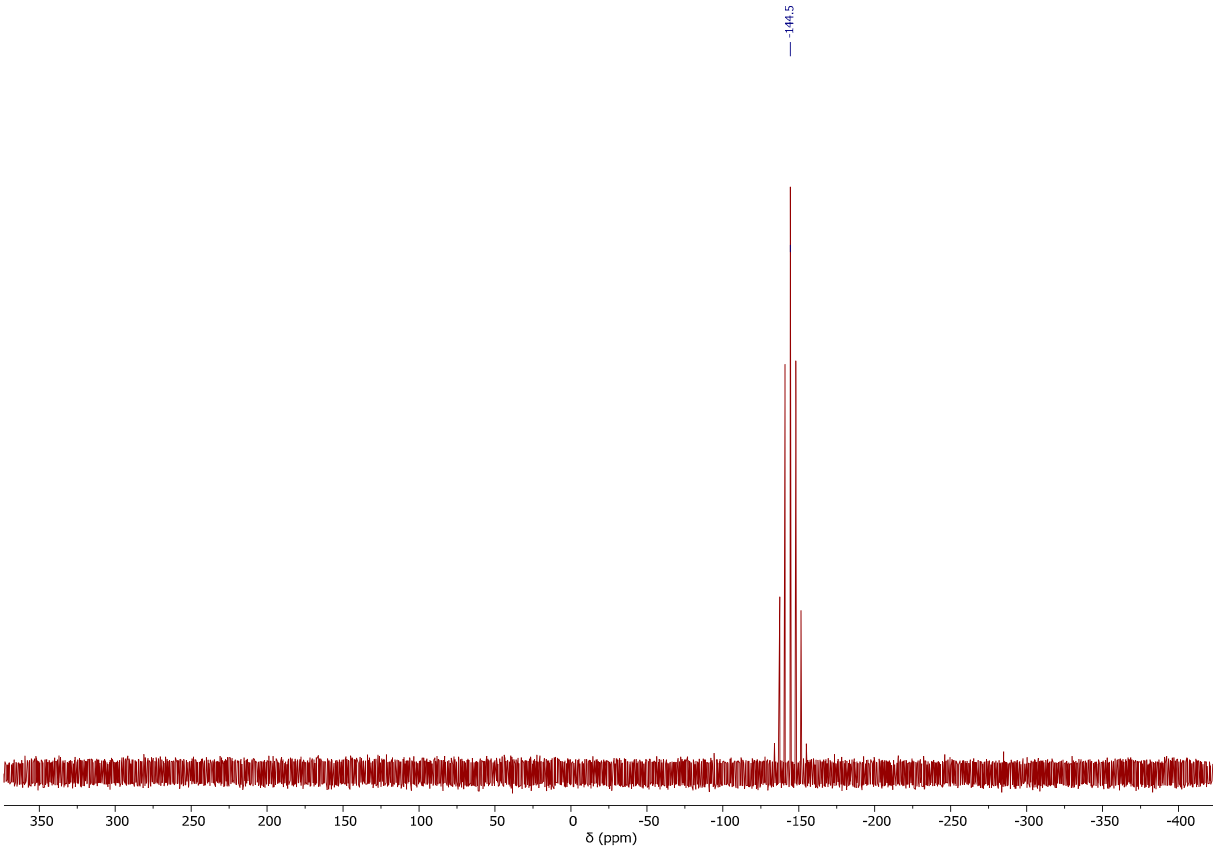

Figure S4. ^{31}P-NMR spectrum (202 MHz, DCM-d_2/MeOD-d_4) of [**PtCO(L)**].

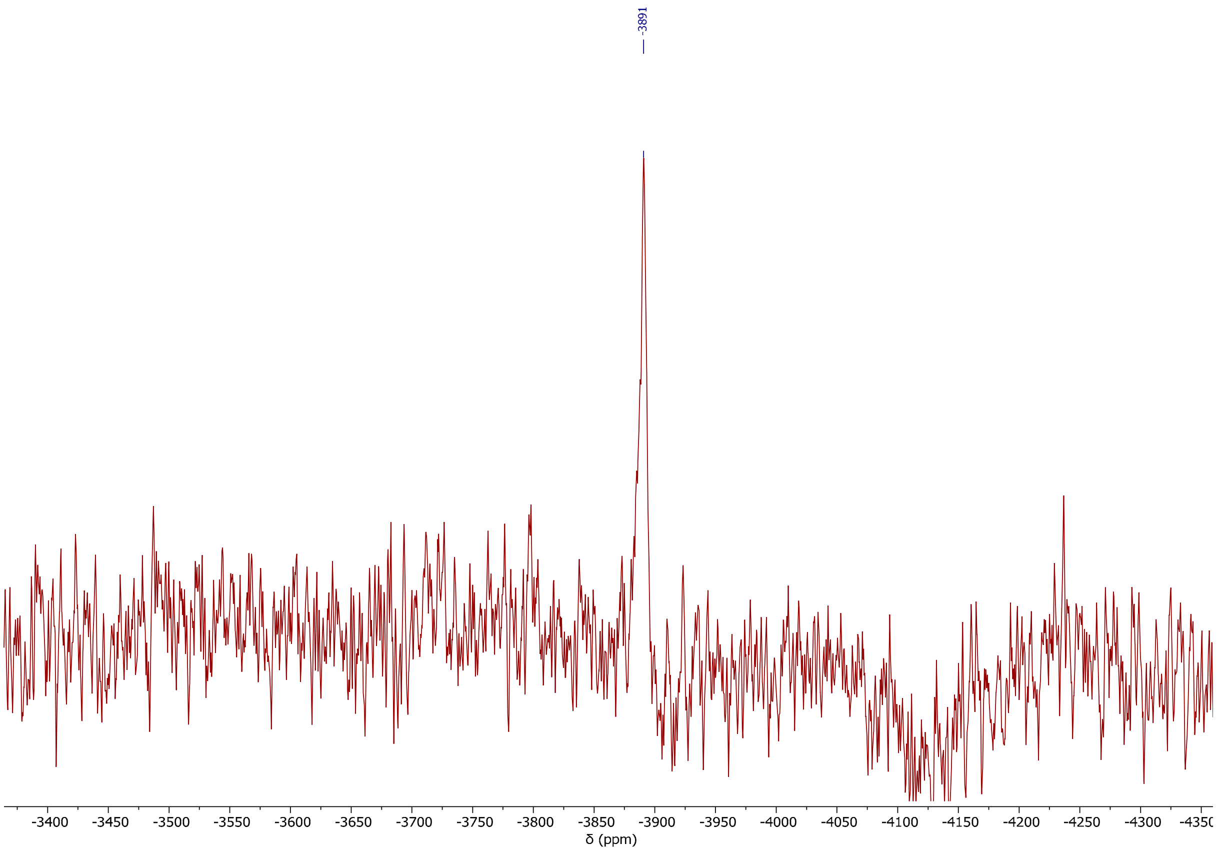

Figure S5. ^{195}Pt-NMR spectrum (107 MHz, DCM-d_2/MeOD-d_4) of [**PtCO(L)**].

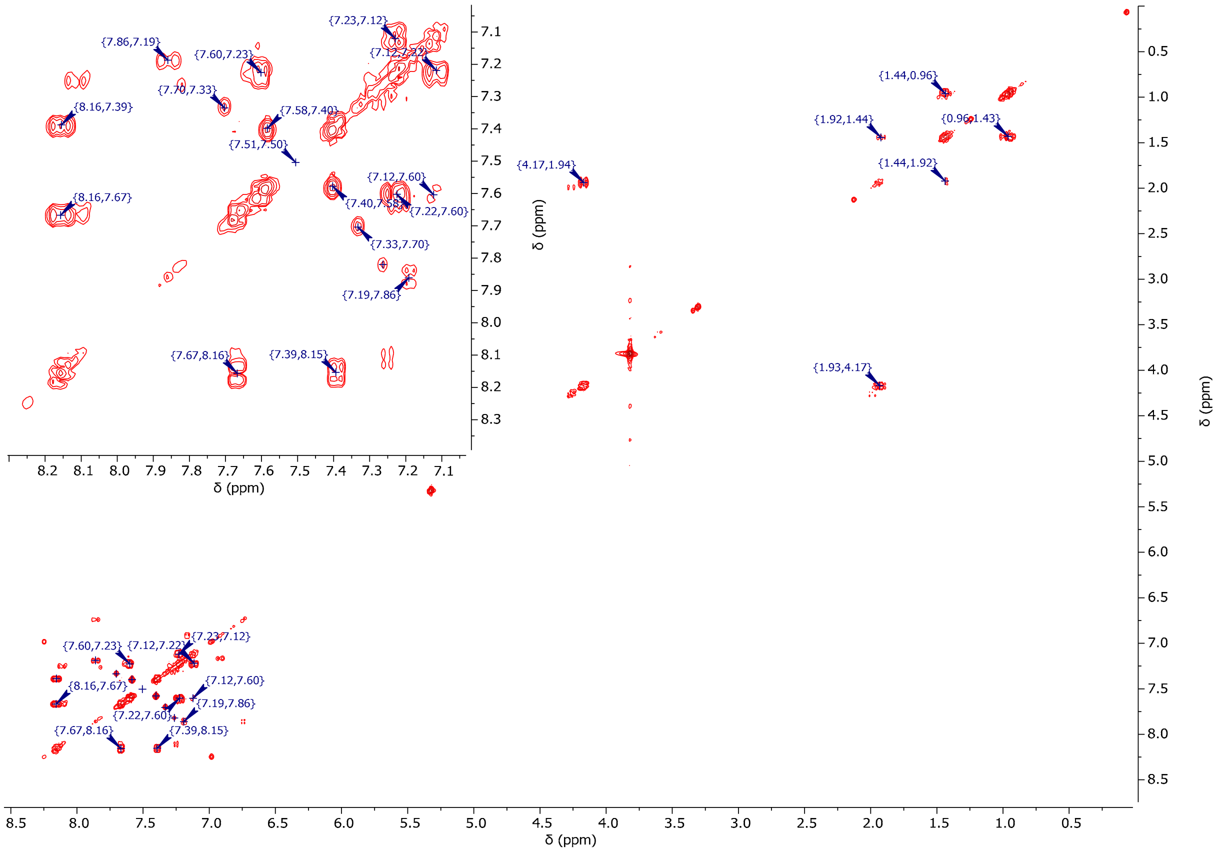

Figure S6. ^{1}H/^{1}H-COSY-NMR spectrum (400 MHz/400 MHz, DCM-d_2/MeOD-d_4) of [**PtCO(L)**].

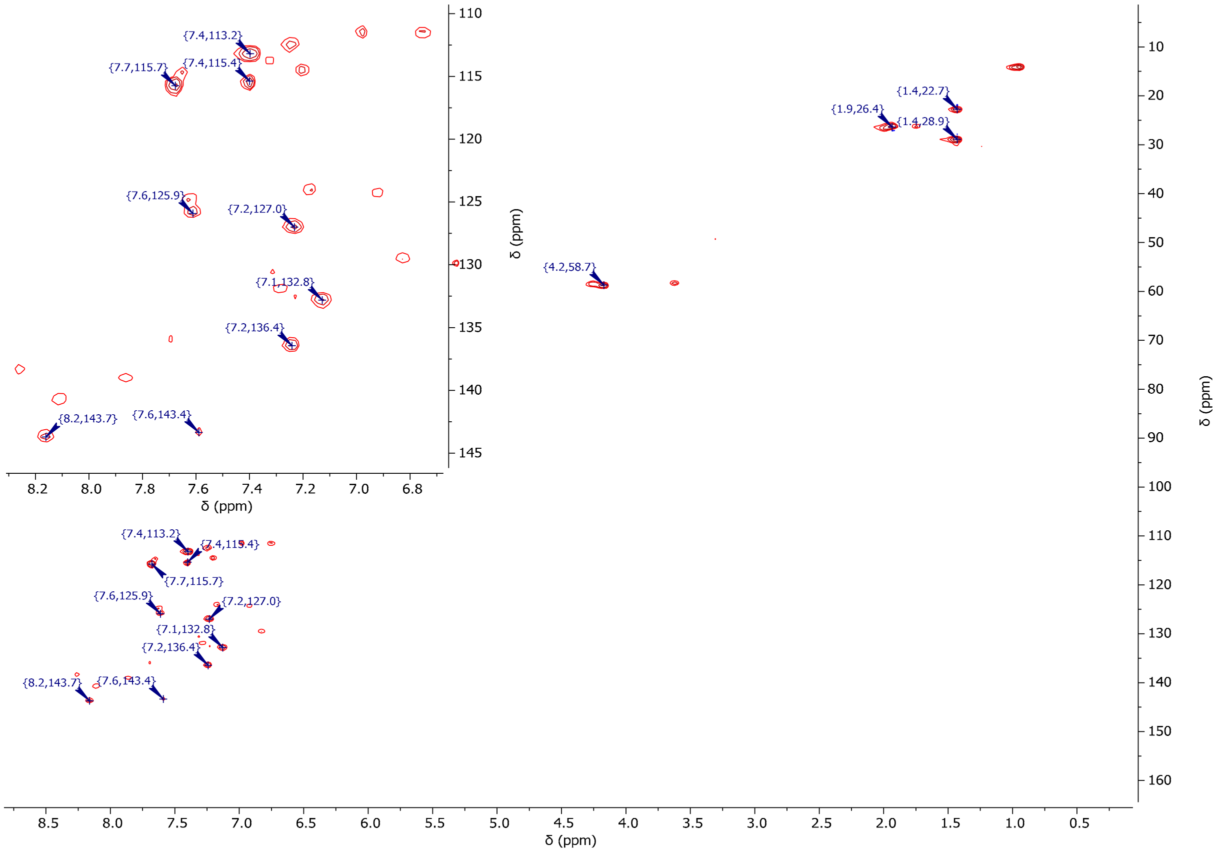

Figure S7. $^{1}H/^{13}C$-gHSQC-NMR spectrum (400 MHz/101 MHz, DCM-d$_2$/MeOD-d$_4$) of [**PtCO(L)**].

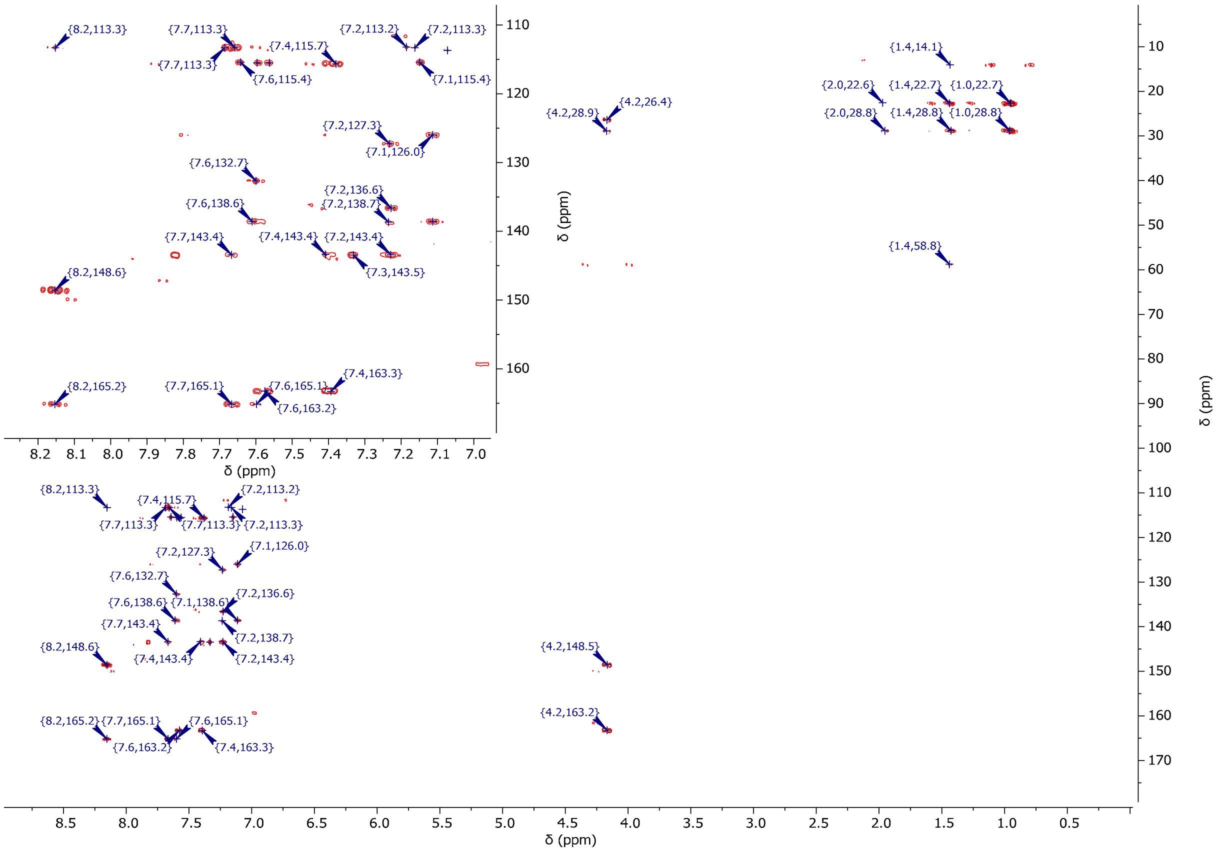

Figure S8. $^{1}H/^{13}C$-gHMBC-NMR spectrum (400 MHz/101 MHz, DCM-d$_2$/MeOD-d$_4$) of [**PtCO(L)**].

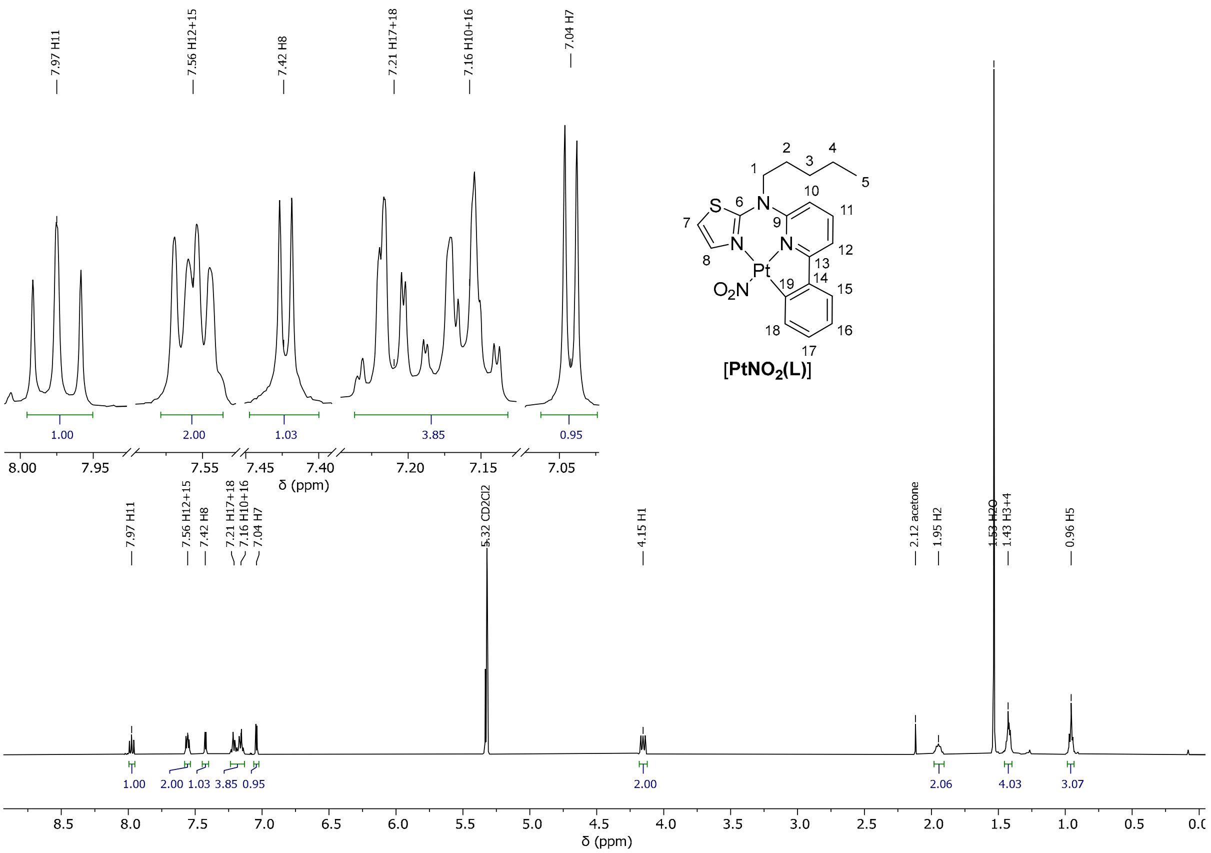

Figure S9. ^{1}H-NMR spectrum (500 MHz, DCM-d$_2$) of **[PtNO$_2$(L)]**.

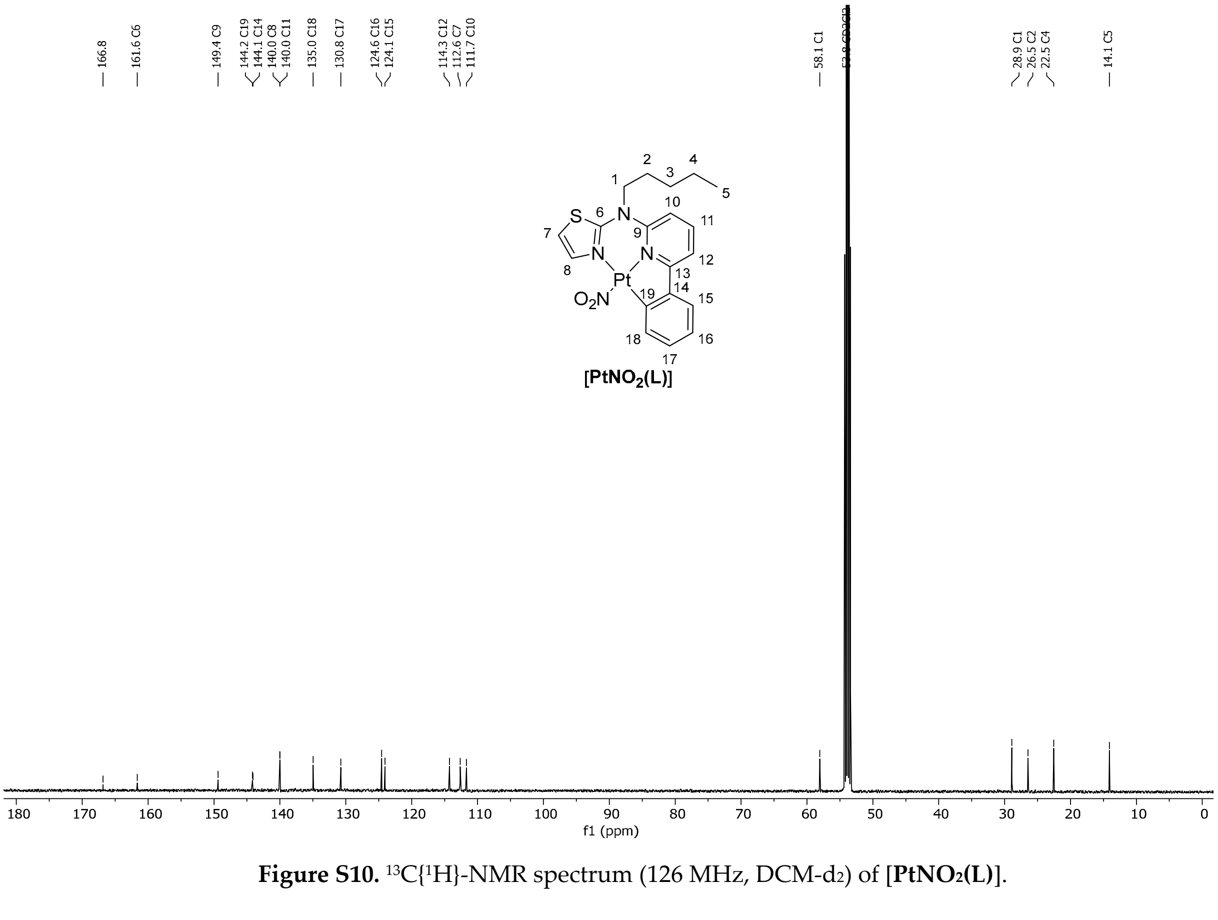

Figure S10. ^{13}C{^{1}H}-NMR spectrum (126 MHz, DCM-d$_2$) of **[PtNO$_2$(L)]**.

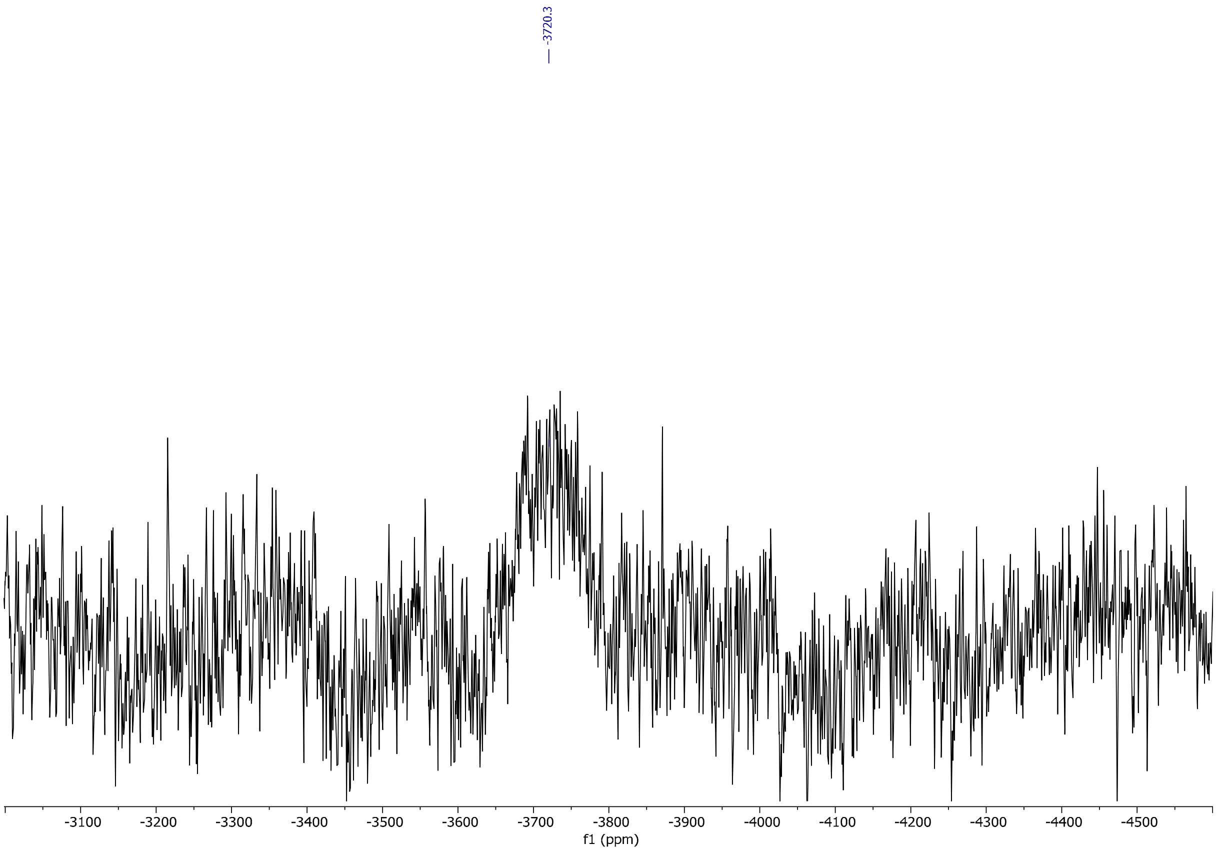

Figure S11. ^{195}Pt-NMR spectrum (107 MHz, DCM-d2) of [**PtNO2(L)**].

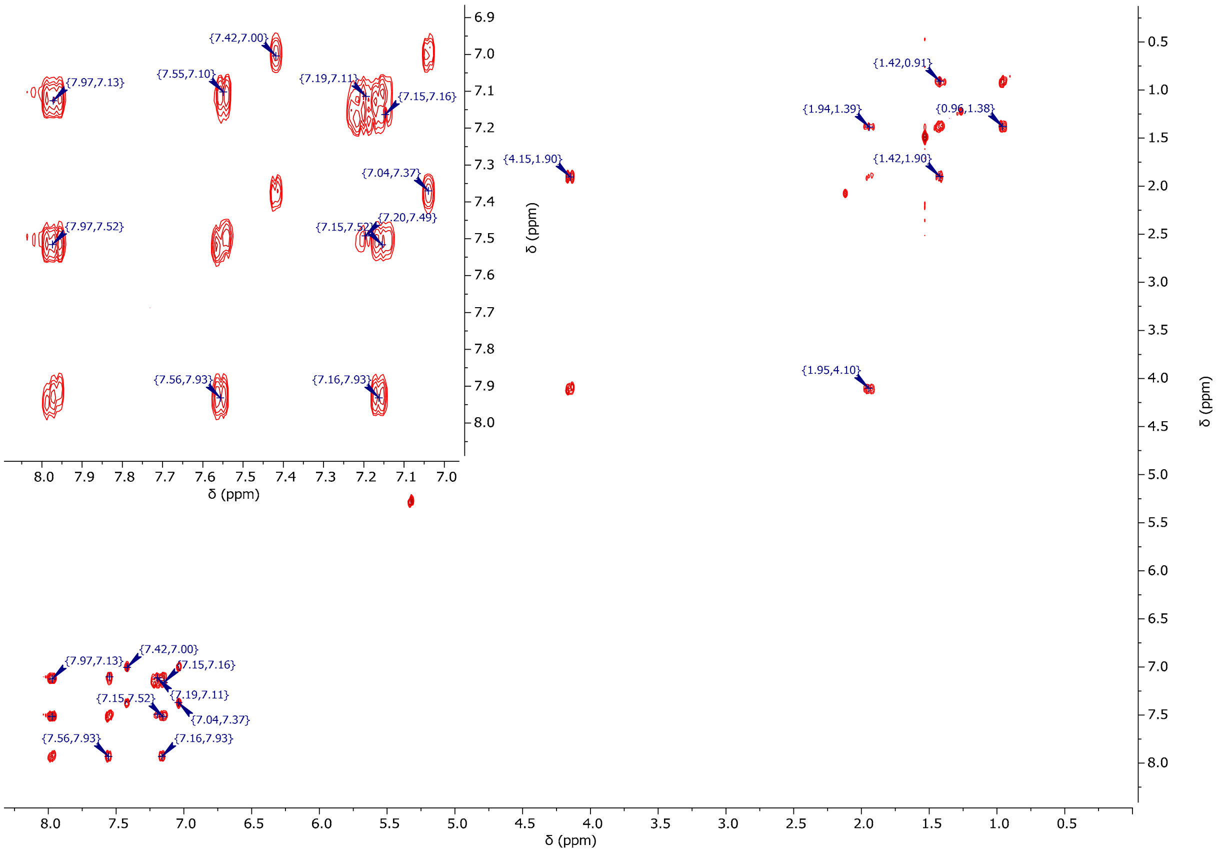

Figure S12. ^{1}H/^{1}H-COSY-NMR spectrum (500 MHz/500 MHz, DCM-d2) of [**PtNO2(L)**].

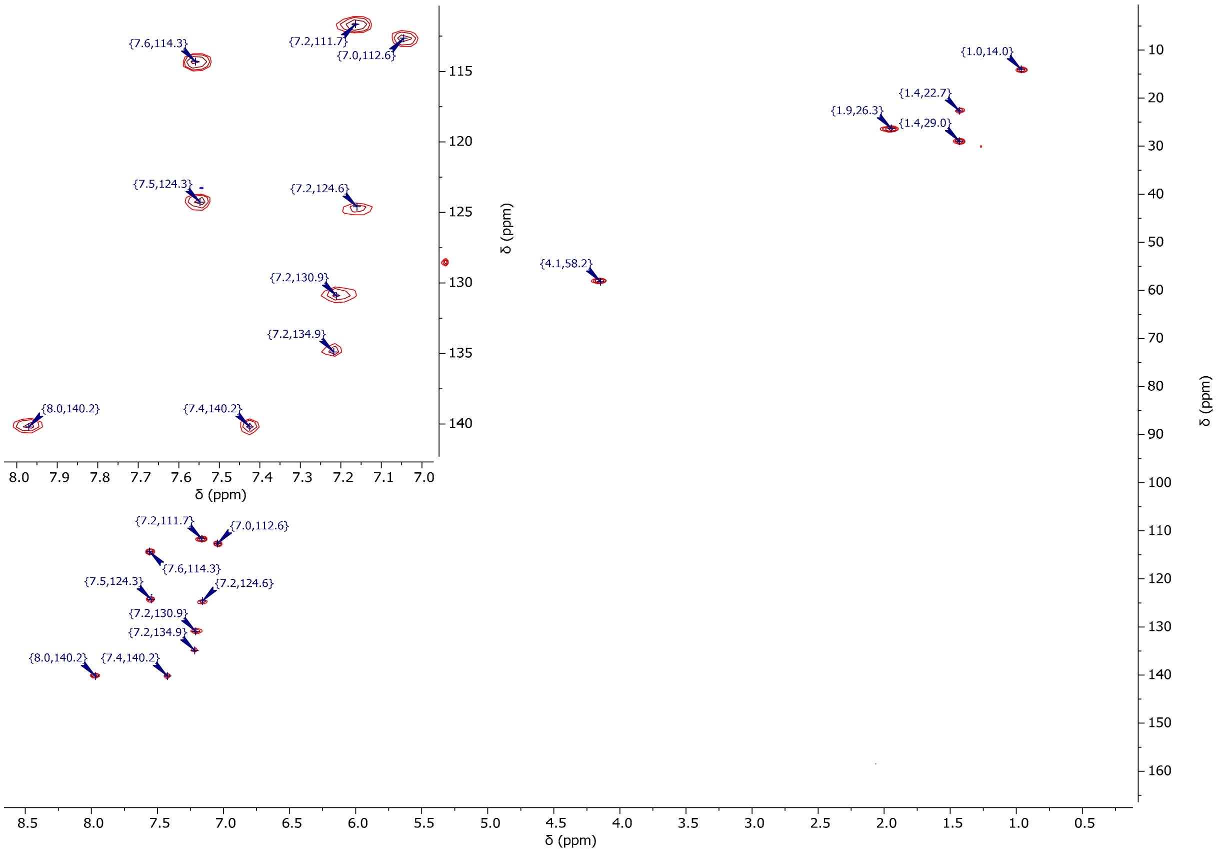

Figure S13. $^{1}H/^{13}C$-gHSQC-NMR spectrum (500 MHz/126 MHz, DCM-d$_2$) of **[PtNO$_2$(L)]**.

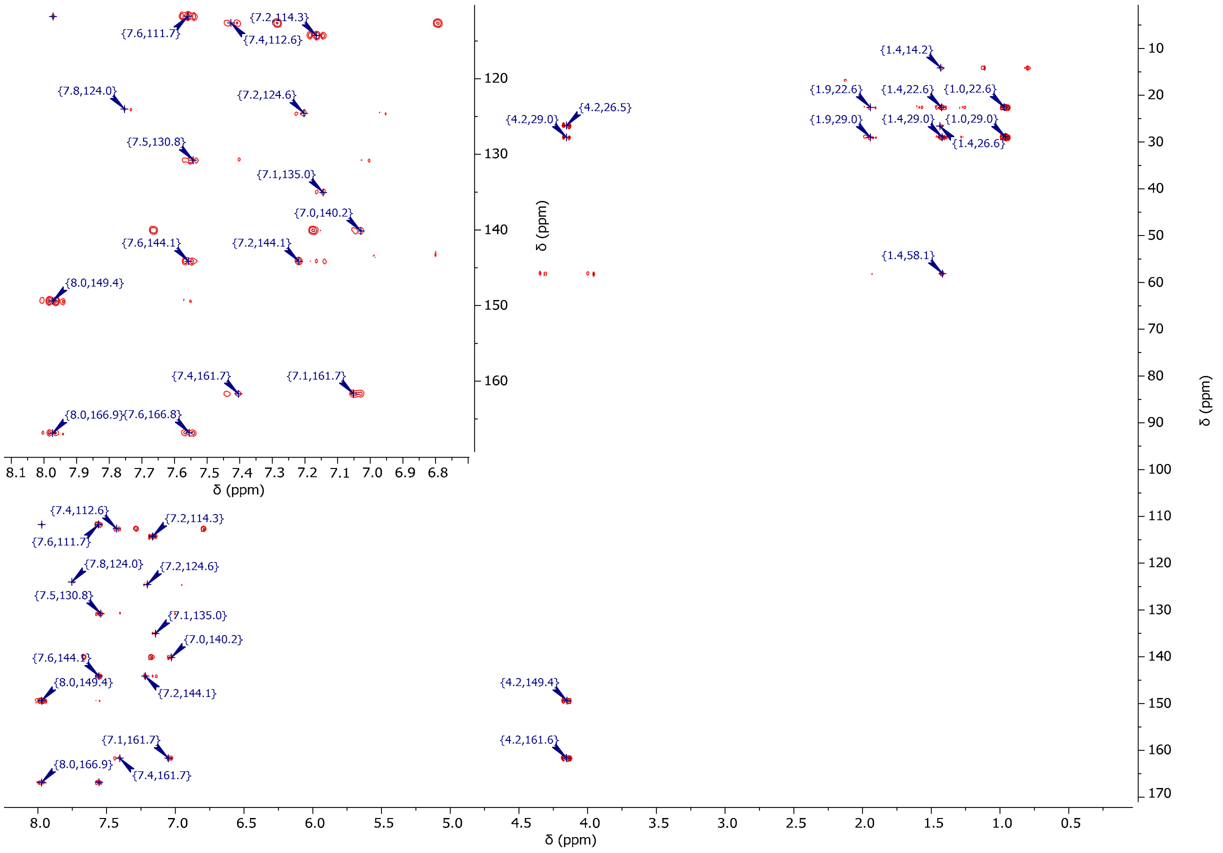

Figure S14. $^{1}H/^{13}C$-gHMBC-NMR spectrum (500 MHz/126 MHz, DCM-d$_2$) of **[PtNO$_2$(L)]**.

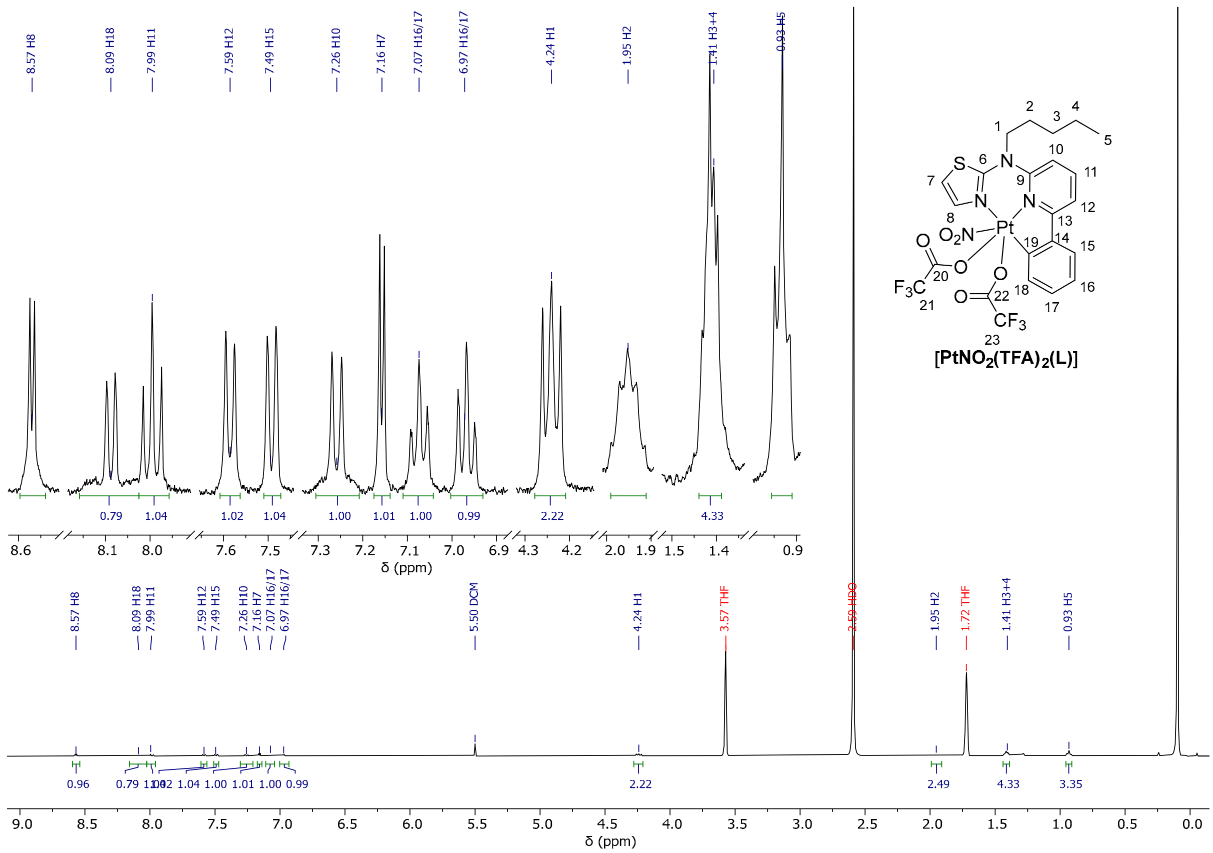

Figure S15. ^{1}H-NMR spectrum (500 MHz, THF-d$_8$) of [**PtNO$_2$(TFA)$_2$(L)**].

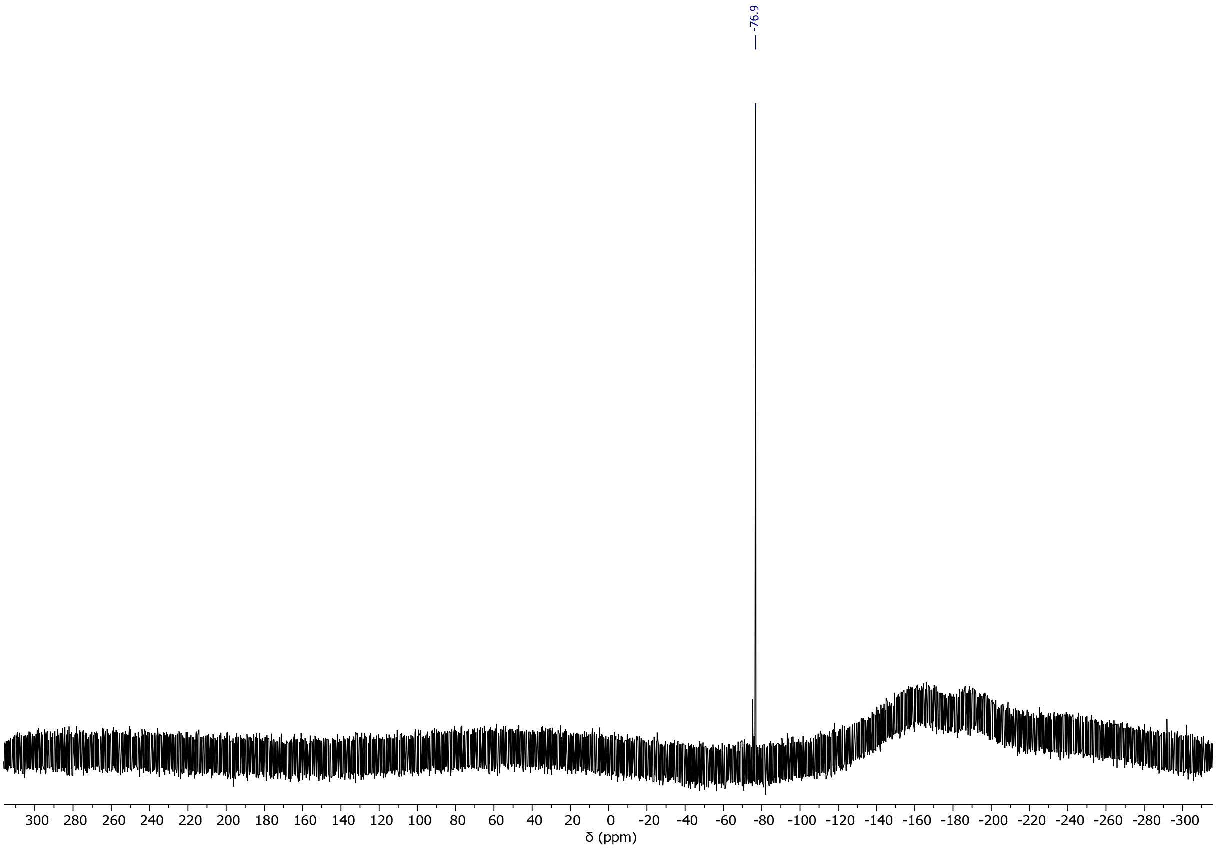

Figure S16. ^{19}F-NMR spectrum (471 MHz, THF-d$_8$) of [**PtNO$_2$(TFA)$_2$(L)**].

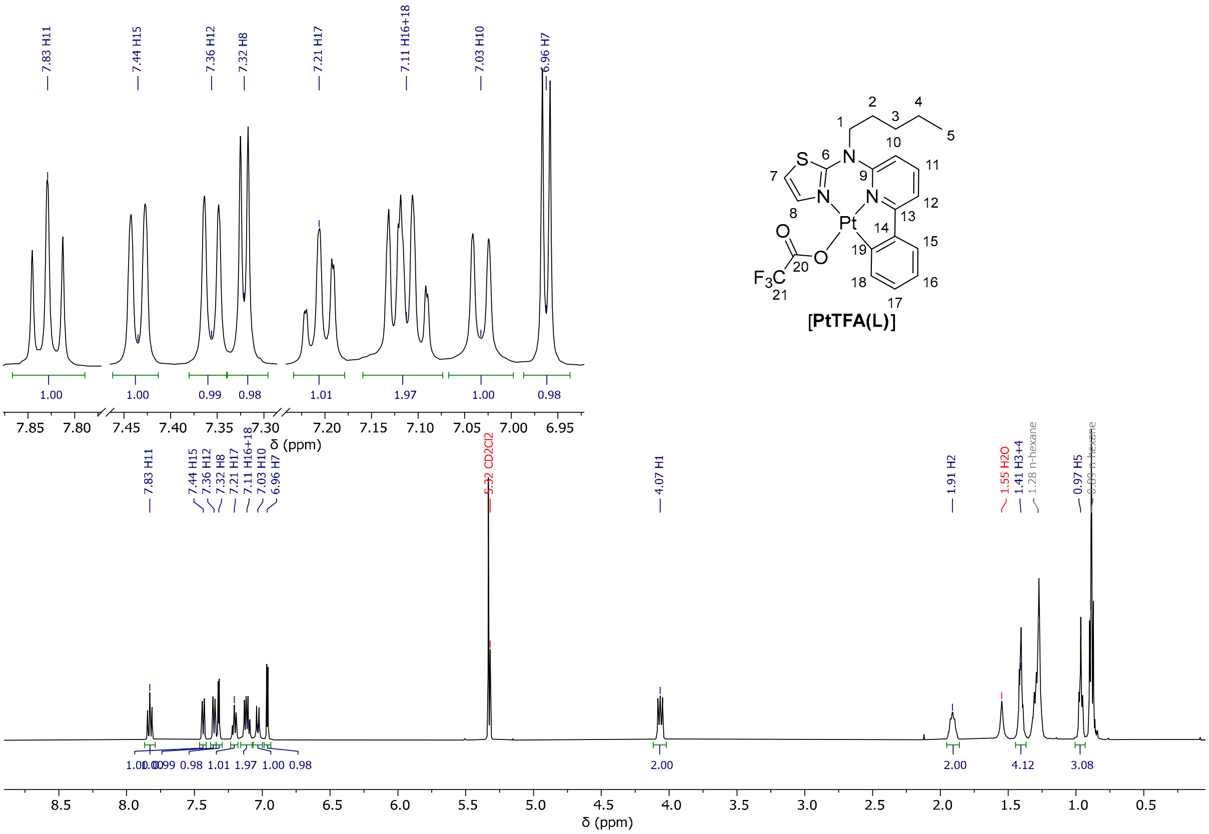

Figure S17. ^{1}H-NMR spectrum (500 MHz, DCM-d_2) of **[PtTFA(L)]**.

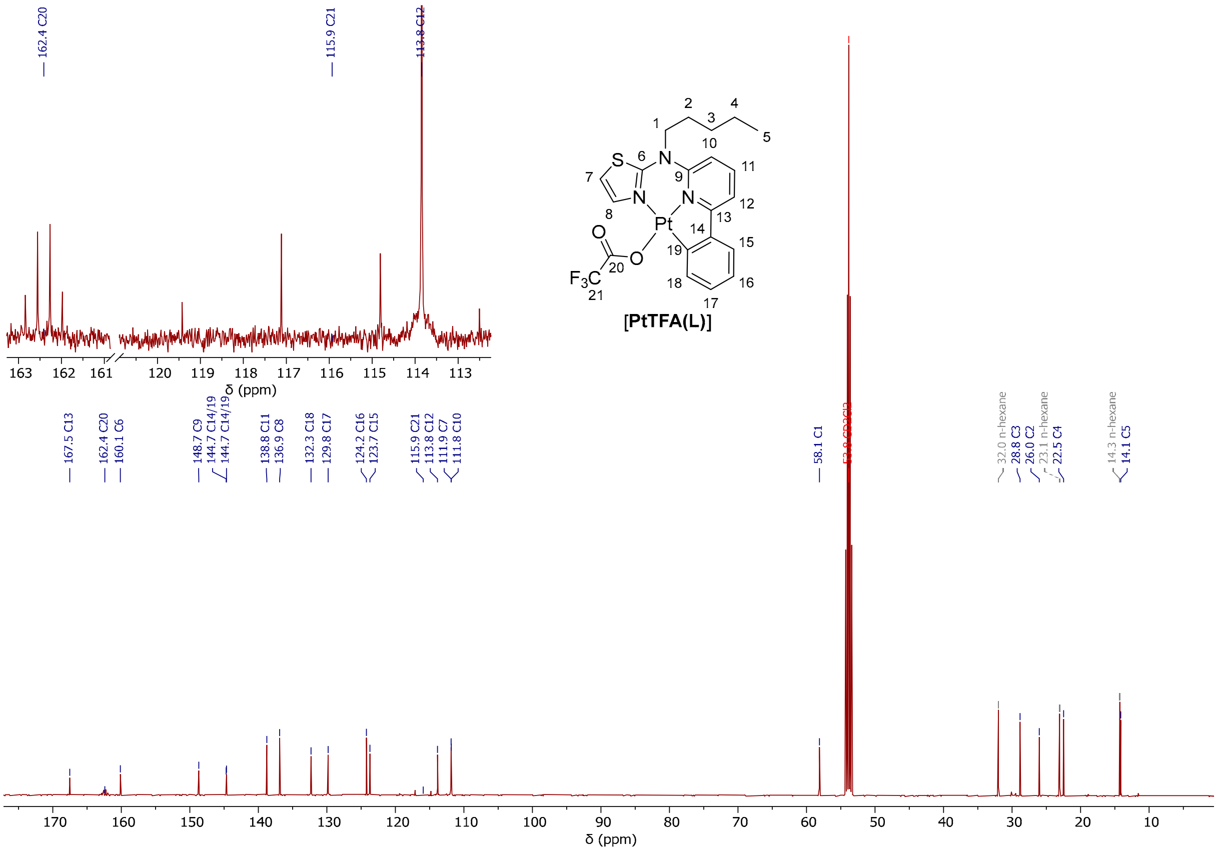

Figure S18. $^{13}C\{^{1}H\}$-NMR spectrum (126 MHz, DCM-d_2) of **[PtTFA(L)]**.

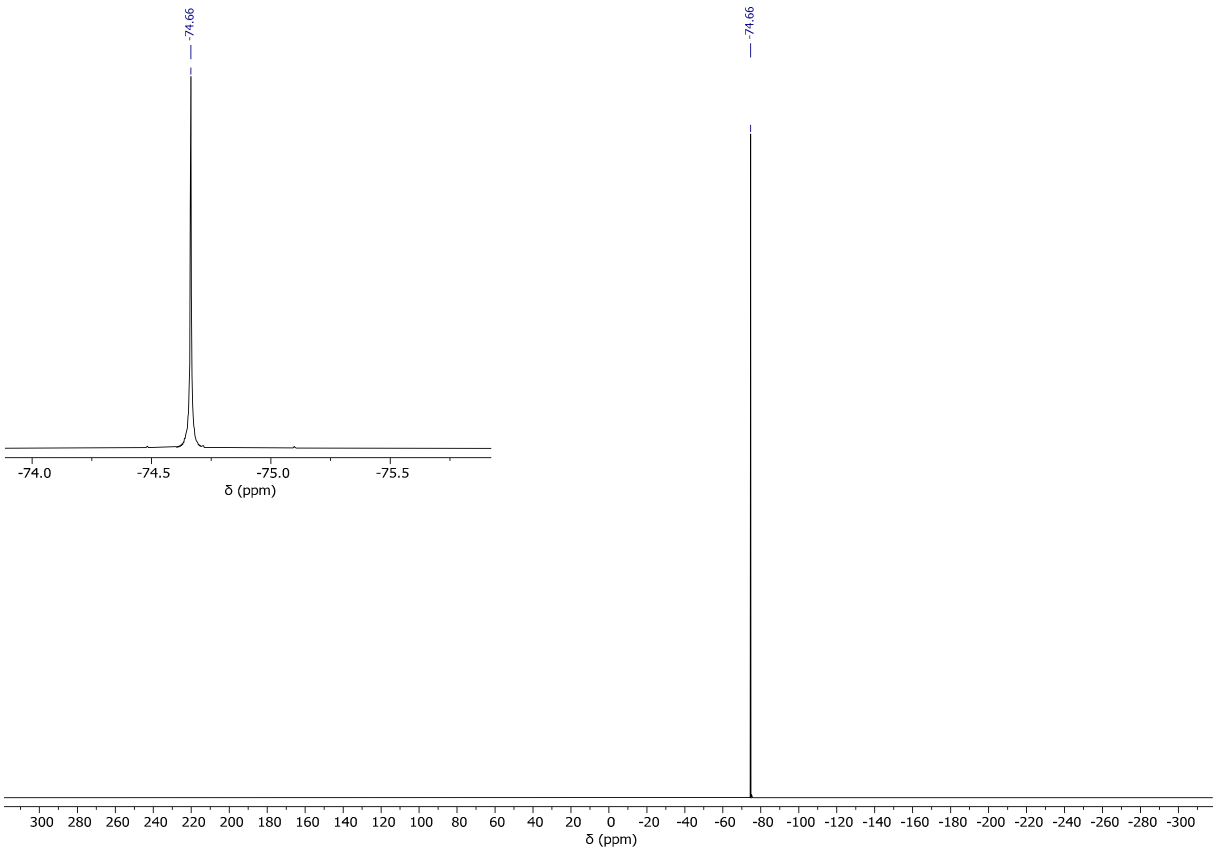

Figure S19. ^{19}F-NMR spectrum (471 MHz, DCM-d$_2$) of [**PtTFA(L)**].

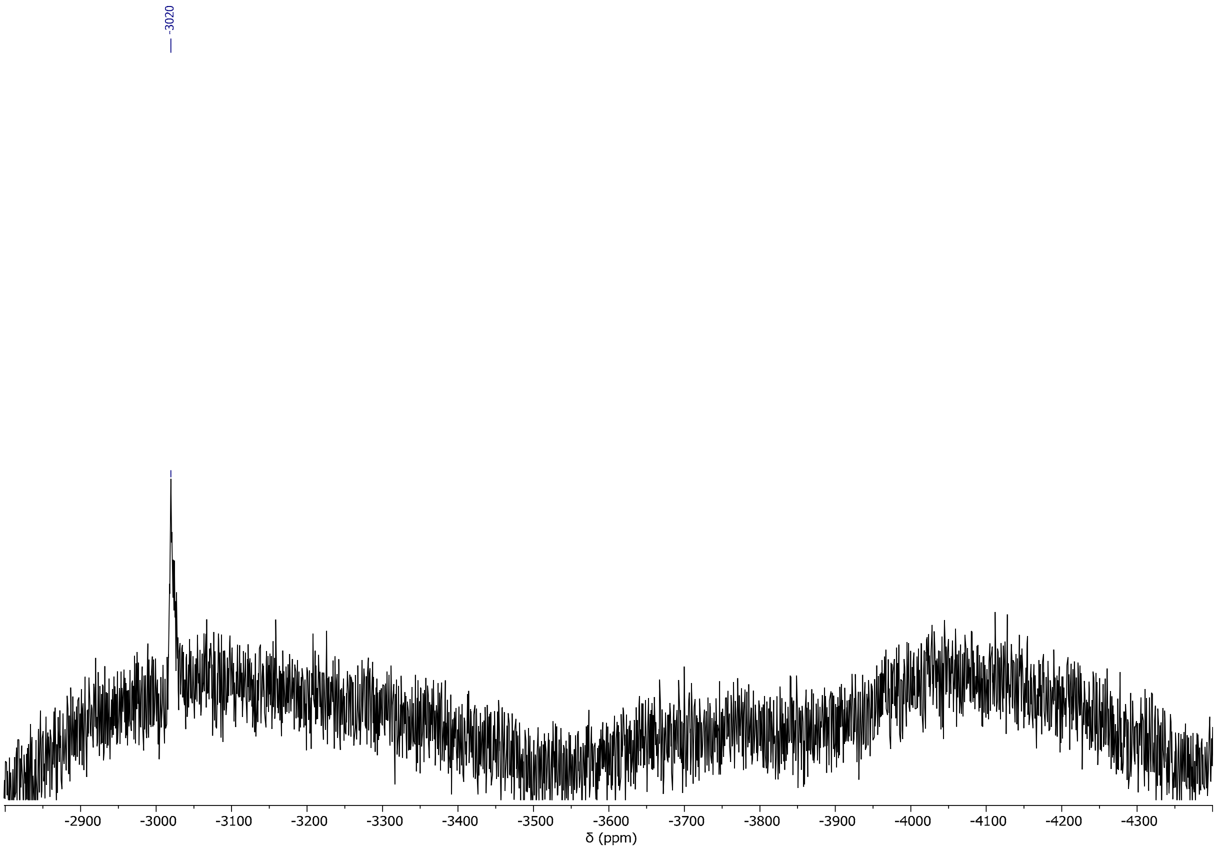

Figure S20. ^{195}Pt-NMR spectrum (107 MHz, DCM-d$_2$) of [**PtTFA(L)**].

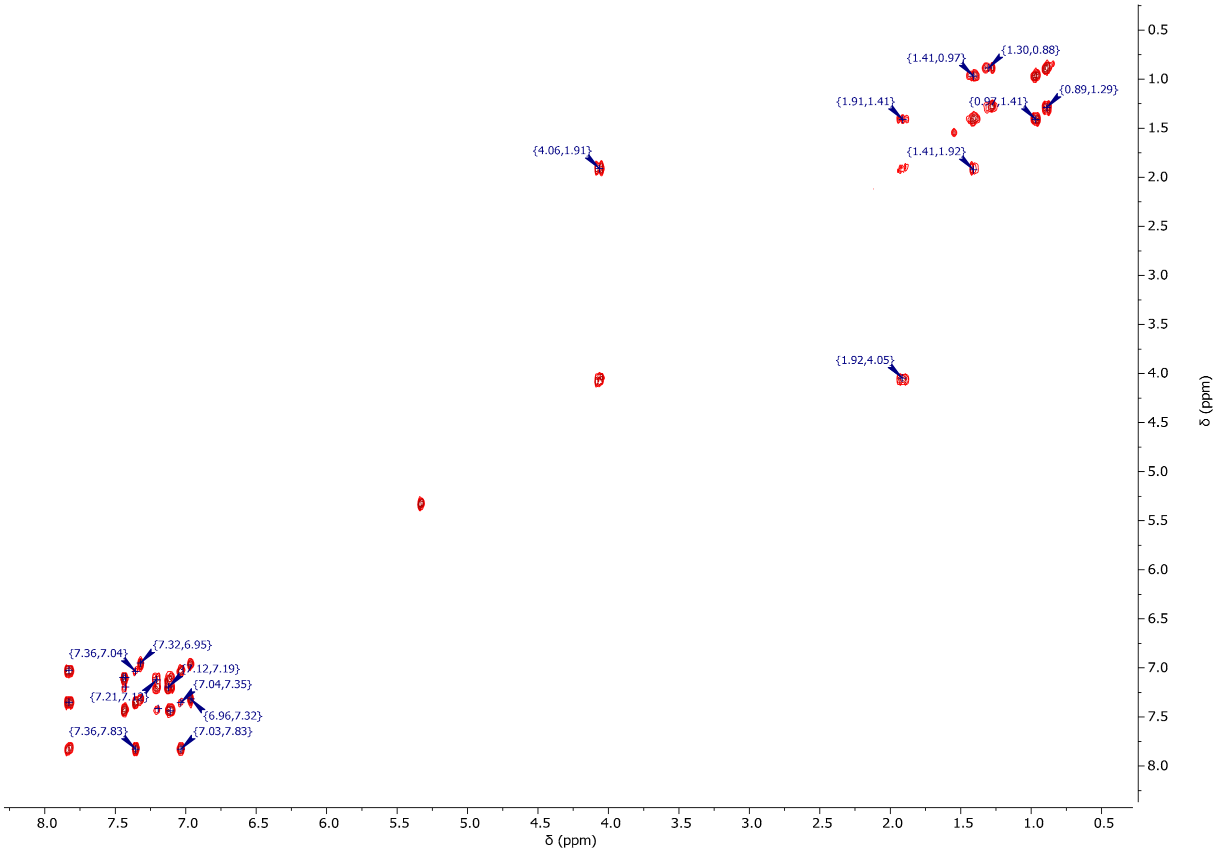

Figure S21. $^1H/^1H$-COSY-NMR spectrum (500 MHz/500 MHz, DCM-d_2) of [**PtTFA(L)**].

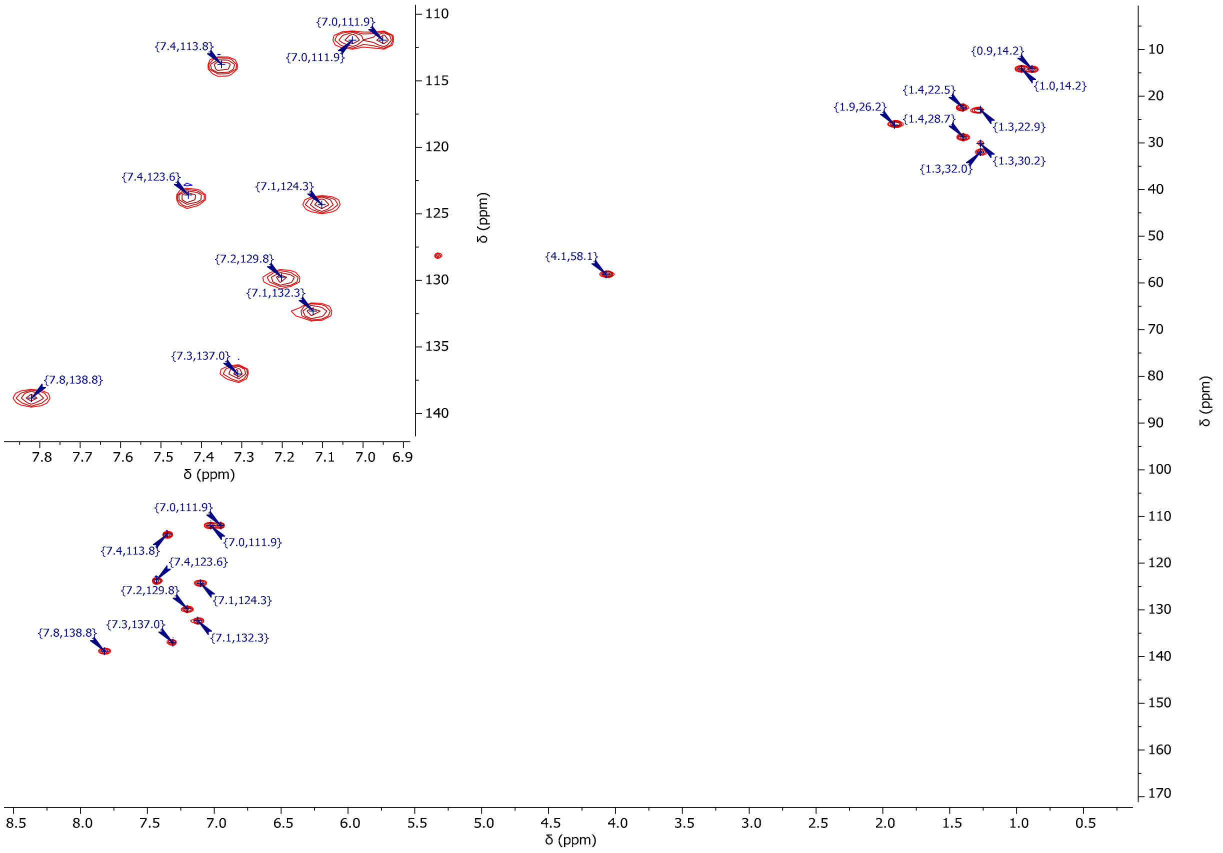

Figure S22. $^1H/^{13}C$-gHSQC-NMR spectrum (500 MHz/126 MHz, DCM-d_2) of [**PtTFA(L)**].

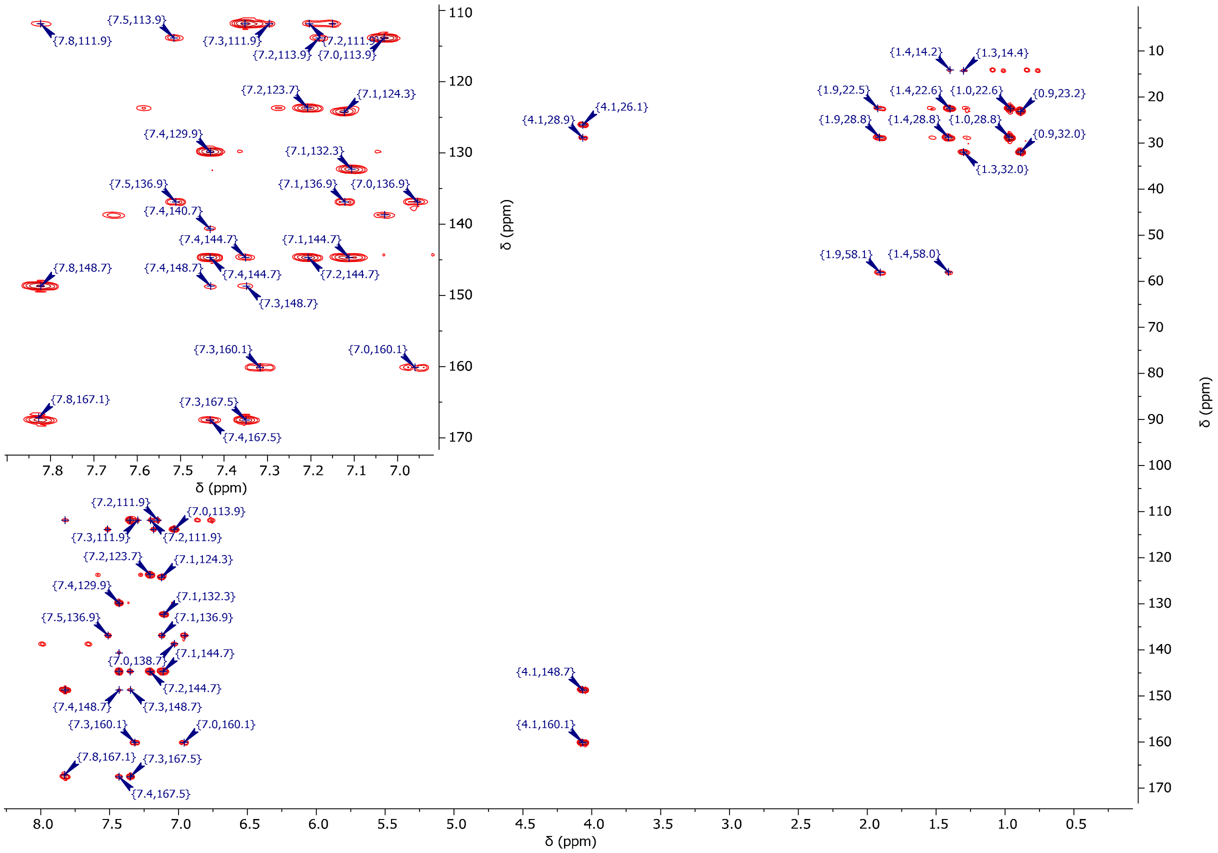

Figure S23. $^{1}H/^{13}C$-gHMBC-NMR spectrum (500 MHz/126 MHz, DCM-d$_2$) of **[PtTFA(L)]**.

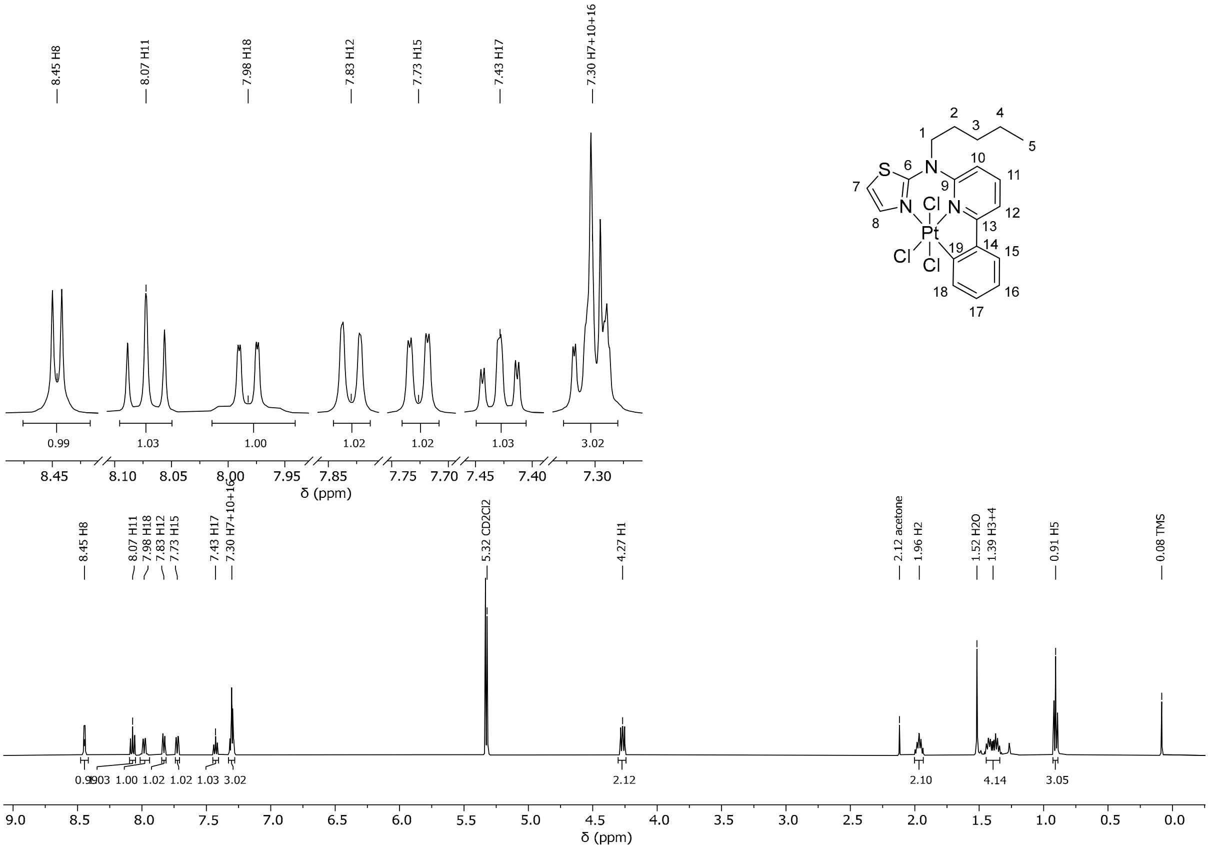

Figure S24. ^{1}H-NMR spectrum (500 MHz, DCM-d$_2$) of **[PtCl$_3$(L)]**.

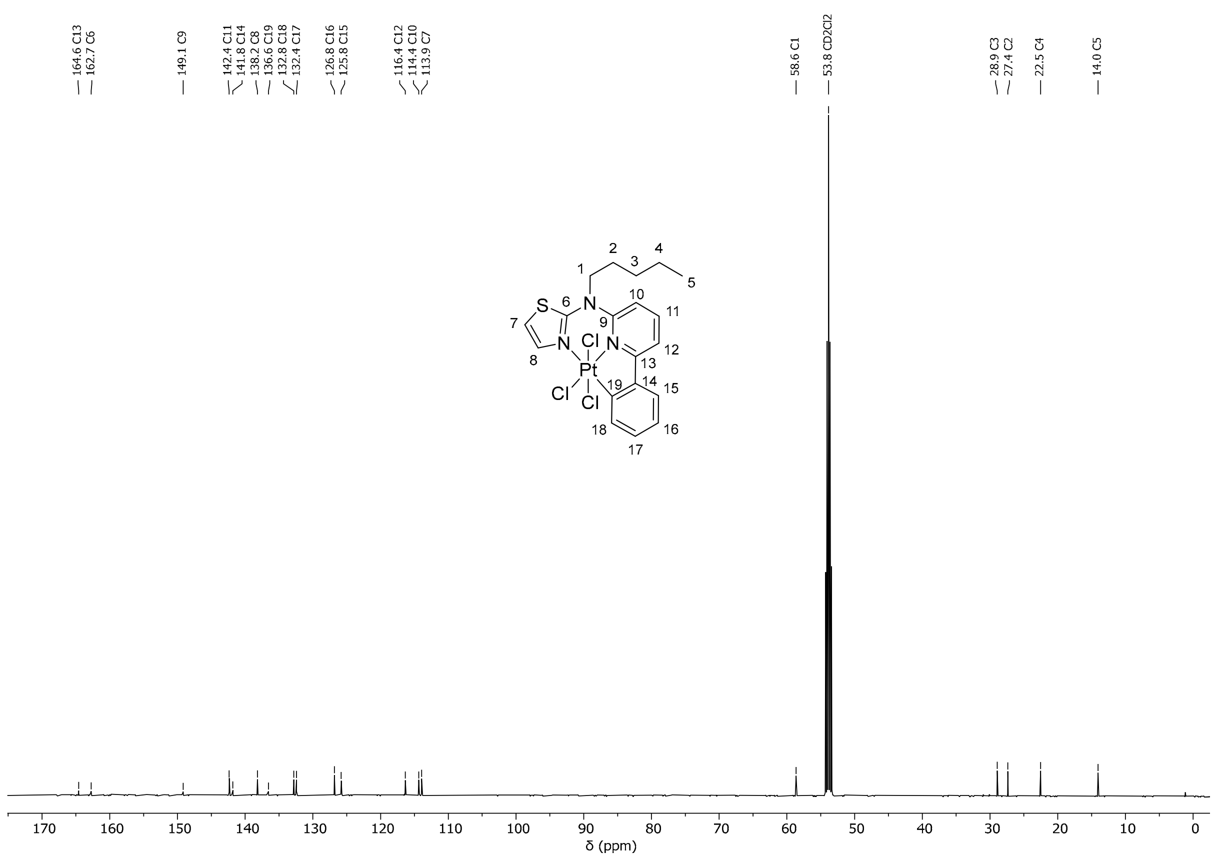

Figure S25. $^{13}C\{^1H\}$-NMR spectrum (126 MHz, DCM-d$_2$) of [**PtCl$_3$(L)**].

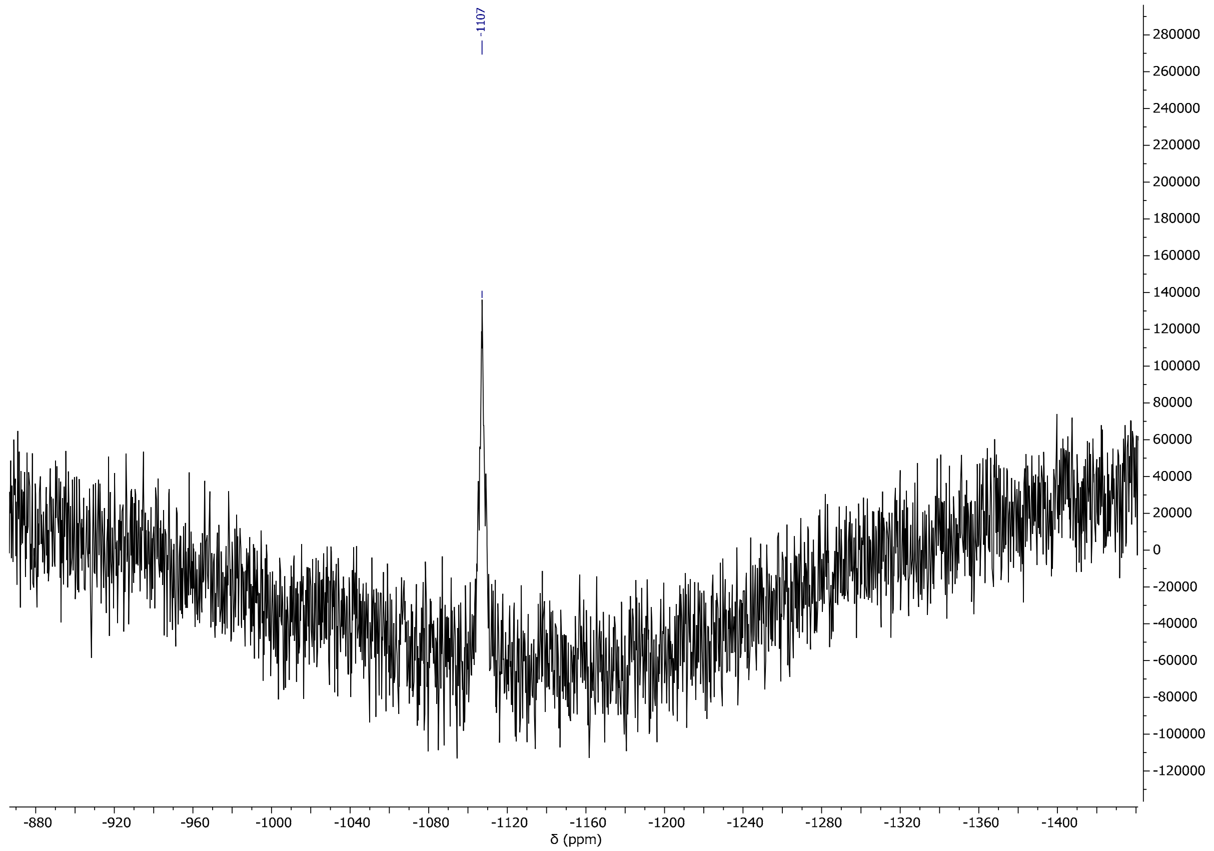

Figure S26. ^{195}Pt-NMR spectrum (107 MHz, DCM-d$_2$) of [**PtCl$_3$(L)**].

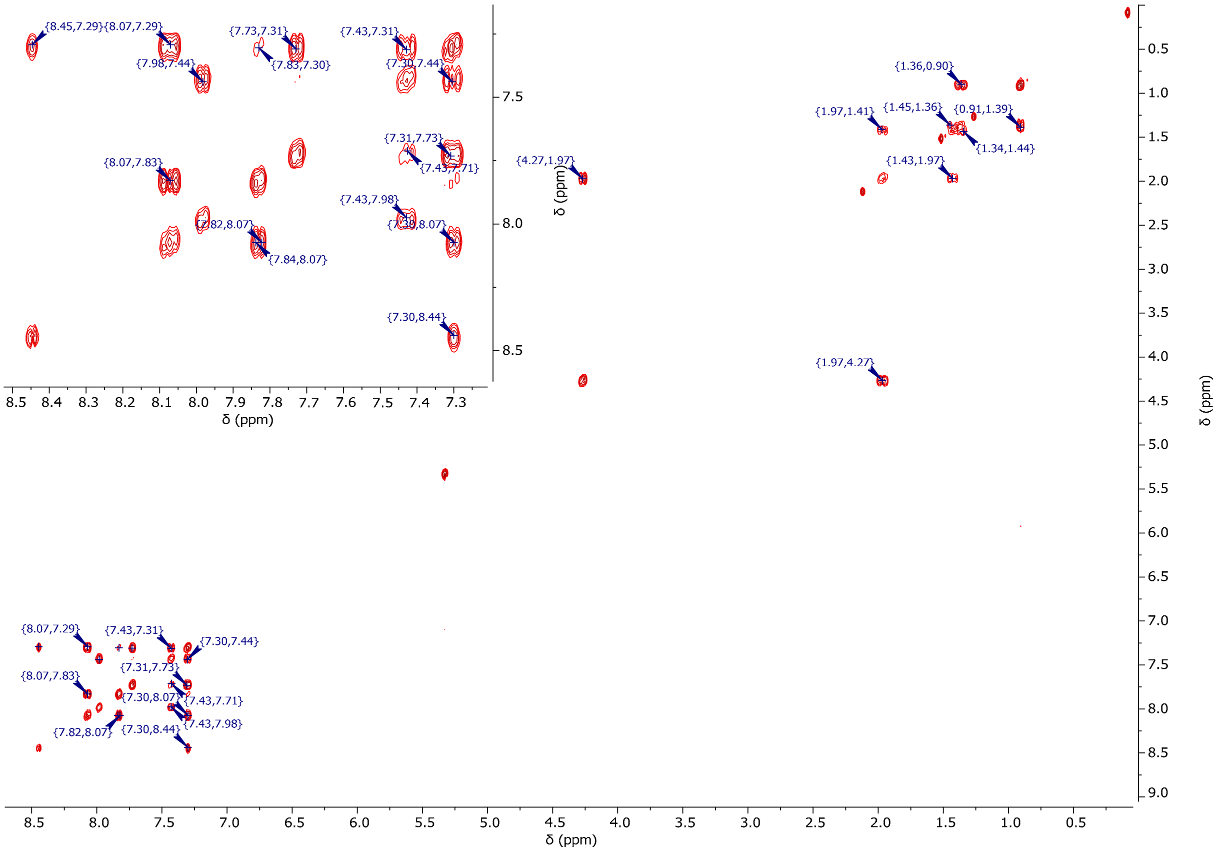

Figure S27. $^{1}H/^{1}H$-COSY-NMR spectrum (500 MHz/500 MHz, DCM-d_2) of **[PtCl$_3$(L)]**.

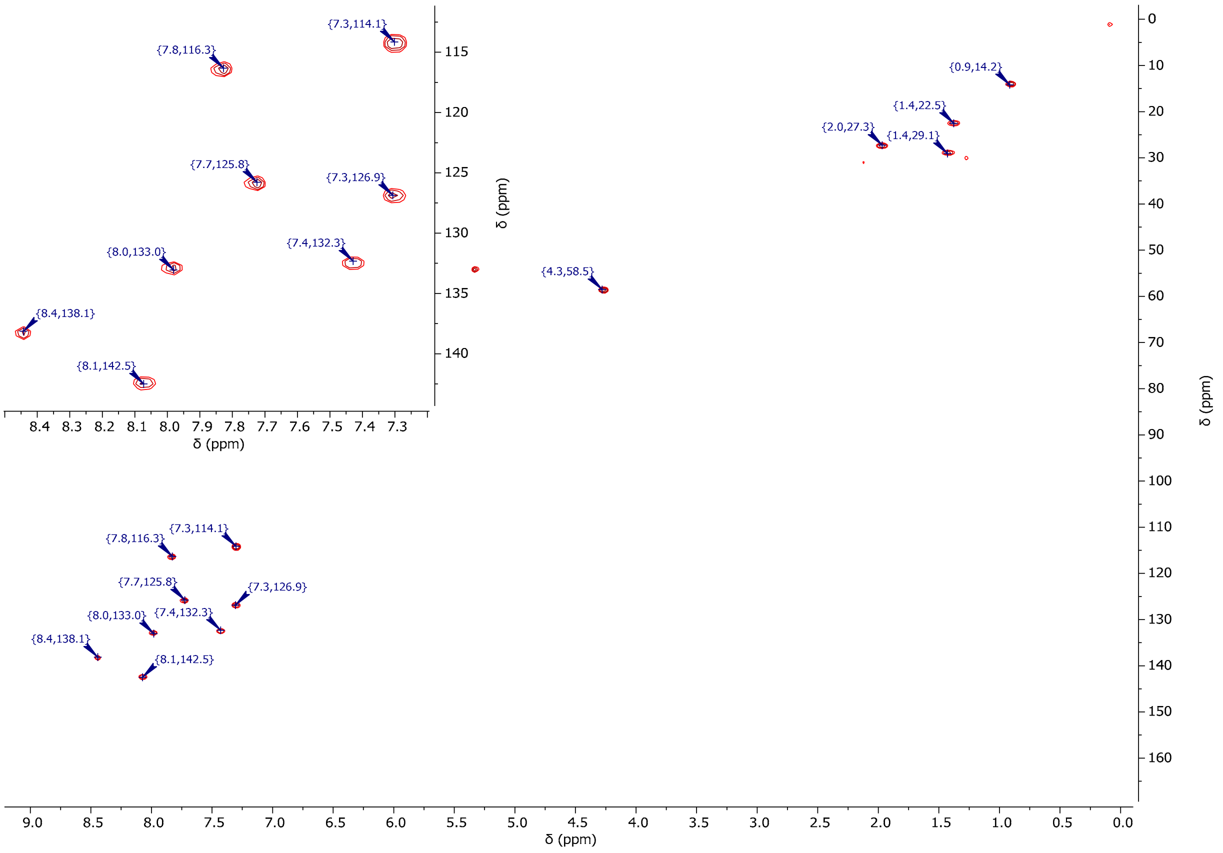

Figure S28. $^{1}H/^{13}C$-gHSQC-NMR spectrum (500 MHz/126 MHz, DCM-d_2) of **[PtCl$_3$(L)]**.

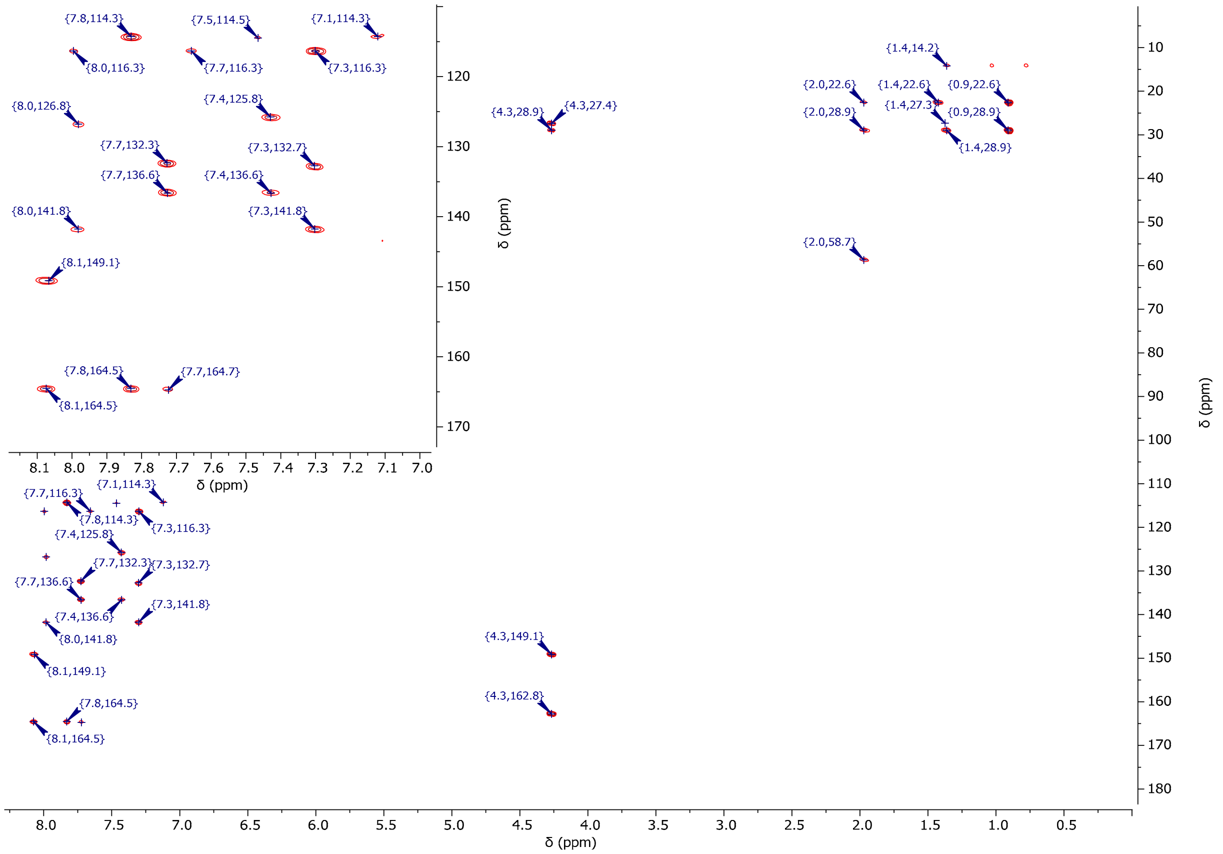

Figure S29. $^{1}H/^{13}C$-gHMBC-NMR spectrum (500 MHz/126 MHz, DCM-d_2) of **[PtCl$_3$(L)]**.

I.3 X-ray diffractometric analysis

Single crystals suitable for measurements of **[PtNO$_2$(L)]** and **[Pt(TFA)$_2$NO$_2$(L)]** were obtained by slowly evaporating the solvent of a saturated DCM solution of the compound or diffusing cyclohexane or *n*-hexane to a DCM solution. The single crystal for **[PtCl$_3$(L)]** was obtained by slowly evaporating a saturated $CHCl_3$ solution of **[PtCl(L)]**. Data sets for compound **[PtNO$_2$(L)]** and **[Pt(TFA)$_2$NO$_2$(L)]** were collected on a Bruker APEX II Quazar. Data sets for compound **[PtTFA(L)]** and **[PtCl$_3$(L)]** were collected on a Bruker D8 Venture.

For compound **[PtNO$_2$(L)]**, an alert A was obtained: PLAT973_ALERT_2_A Check Calcd. Positive Resid. Density on Pt1 2.08 eA^{-3}. This is a known alert for heavy metals and is probably due to difficulties in (or inefficiency in) absorption correction.[77] Software used to prepare material for publication: Mercury.

Table S1. Parameters and data from X-ray diffractometry on single crystals of the Pt(II) complexes.

**Complex**	**[PtNO$_2$(L)]**	**[PtTFA(L)]**
**Formula**	$C_{19}H_{20}N_4O_2SPt$	$C_{21}H_{20}N_3O_2SF_3Pt$
**Fw**	563.54	630.55
**CCDC No.**	2298207	2298208
**Temperature/K**	101(2)	105(2)
**Crystal color**	rods, yellow	rods, yellow
**Crystal system**	trigonal	triclinic
Space group	*R*-3:H	*P*-1
***a*/Å**	32.7842(6)	8.1197(2)
***b*/Å**	32.7842(6)	10.0406(3)

***c*/Å**	8.8154(3)	13.5347(4)
***α*/°**	90	89.298(1)
***β*/°**	90	85.524(1)
***γ*/°**	120	74.169(1)
***V*/Å^3**	8205.4(4)	1058.30(5)
***Z*-value**	18	2
**Calculated density/g cm^{-3}**	2.053	1.979
**Crystal size/mm^3**	0.45 × 0.15 × 0.14	0.27 × 0.11 × 0.06
**Radiation/wavelength/pm**	Mo*Kα* / 71.073	Mo*Kα* / 71.073
***μ*(MoK*α*)/mm^{-1}**	7.83	6.78
**F000**	4896	608
***θ* range, deg**	2.15–27.86	2.58–27.88
**h,k,l_{max}**	±43, ±43, ±11	±10, ±13, ±17
**T_{min},T_{max}**	0.300, 0.746	0.478, 0.746
**Total no. reflections**	41357	14103
**Independent reflections / R_{int}**	4352 / 0.0639	4977 / 0.0215
**Reflections with $I > 2\sigma(I)$ / R_σ**	3696 / 0.0306	4853 / 0.0256
**Data/parameters**	4352 / 292	4977 / 281
**Goodness-of-fit on F^2**	1.079	1.071
***R*1/*wR*2 for $I > 2\sigma(I)$**	0.0360 / 0.0897	0.0162 / 0.0419
***R*1/*wR*2 for all data**	0.0451 / 0.0953	0.0167 / 0.0422
**Larg. diff. peak/hole/e Å^{-3}**	2.53 / –0.66	1.62 / –0.80

Table S2. Parameters and data from X-ray diffractometry on single crystals of the Pt(IV) complexes.

**Complex**	**[Pt(TFA)$_2$NO$_2$(L)]**	**[PtCl$_3$(L)]**
**Formula**	$C_{23}H_{20}N_4O_6SF_6Pt$	$C_{19}H_{20}N_3SPtCl_3$
**Fw**	789.58	623.88
**CCDC No.**	2298209	2298210
**Temperature/K**	100(2)	100(2)
**Crystal color**	block, colourless	botryoidal, yellow
**Crystal system**	monoclinic	monoclinic
Space group	*P*2$_1$/*c*	*P*2$_1$/*c*
***a*/Å**	11.628(11)	10.5848(4)
***b*/Å**	16.348(16)	14.0274(6)
***c*/Å**	13.292(13)	13.6981(5)
***α*/°**	90	90
***β*/°**	90.963(19)	101.863(1)
***γ*/°**	90	90
***V*/Å^3**	2526(4)	1990.42(14)
***Z*-value**	4	4
**Calculated density/g cm^{-3}**	2.076	2.082
**Crystal size/mm^3**	0.5 × 0.45 × 0.3	0.25 × 0.22 × 0.14

**Radiation/wavelength/pm**	Mo*Kα* / 71.073	Mo*Kα* / 71.073
**μ(MoKα)/mm^{-1}**	5.73	7.57
**F000**	1528	1200
**θ range, deg**	1.75–27.88	2.10–30.11
**h,k,l$_{max}$**	±15, ±21, ±17	±14, ±19, ±19
**T$_{min}$,T$_{max}$**	0.526, 0.746	0.119, 0.304
**Total no. reflections**	37343	29070
**Independent reflections / R_{int}**	6025 / 0.0415	5856 / 0.0392
**Reflections with $I > 2\sigma(I)$ / R_σ**	5539 / 0.0268	5715 / 0.0267
**Data/parameters**	6025 / 371	5856 / 245
**Goodness-of-fit on F^2**	1.057	1.235
**$R1/wR2$ for $I > 2\sigma(I)$**	0.0277 / 0.0716	0.0216 / 0.0554
**$R1/wR2$ for all data**	0.0307 / 0.0733	0.0222 / 0.0557
**Larg. diff. peak/hole/e Å^{-3}**	2.09 / –0.72	1.20 / –1.64

Structure of [PtNO$_2$(L)] (QcS4_r-3; CCDC-Nr.: 2298207):

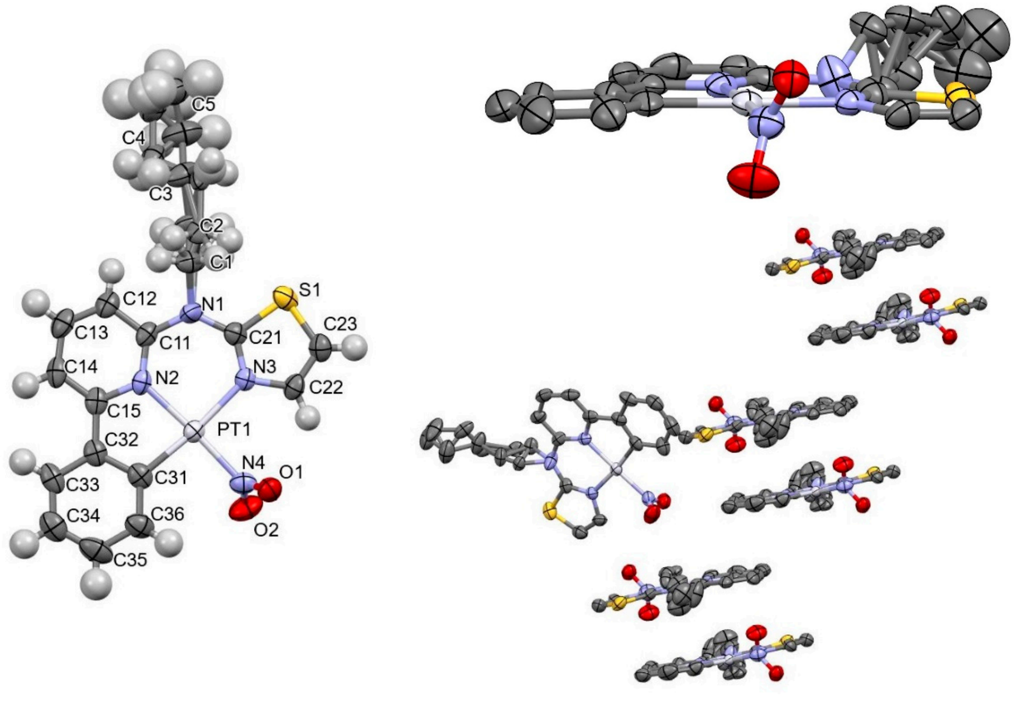

Figure S30. Molecular structure in the single crystal of [**PtNO$_2$(L)**] (left), viewed along the luminophore-Pt plane (right, top) and selected intermolecular interactions and chain formation for the dimers in the crystal structure (right, bottom). Hydrogen atoms are omitted for clarity (right). Displacement ellipsoids are shown at 50 % probability.

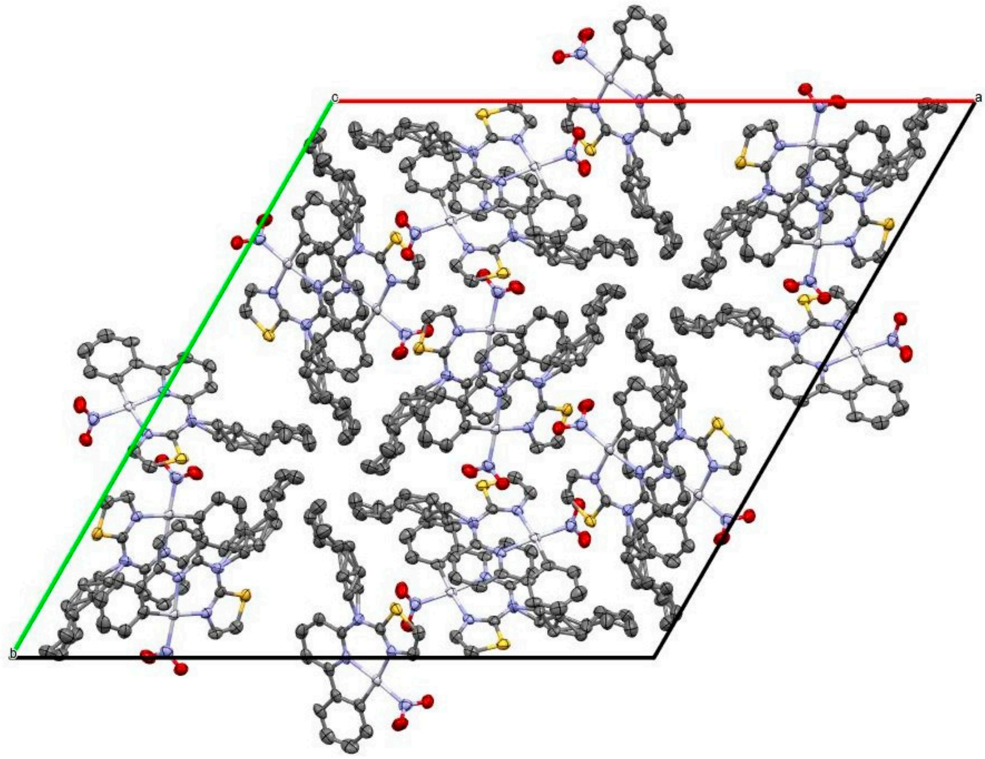

Figure S31. Unit cell for the crystal structure of [**PtNO$_2$(L)**]. Hydrogen atoms are omitted for clarity. Displacement ellipsoids are shown at 50 % probability.

Table S3. Selected bond lengths and angles for [**PtNO$_2$(L)**].

**X-Y**	***d*(X-Y) in Å**	**X-Y-Z**	**∠(XYZ) in °**
Pt1-N2	2.014(4)	N2-Pt1-N3	91.41(19)
Pt1-N3	2.054(5)	N3-Pt1-C31	174.1(2)
Pt1-C31	1.970(6)	C31-Pt1-N4	94.0(2)
Pt1-N4	1.999(5)	N4-Pt1-N2	176.2(2)
		N2-Pt1-C31	82.7(2)
		N3-Pt1-N4	91.90(19)

Structure of [Pt(TFA)$_2$NO$_2$(L)] (sb200610_0m; CCDC-Nr.: 2298209):

Copyright: © 2023 by the authors. Licensee MDPI, Basel, Switzerland. This article is an open access article distributed under the terms and conditions of the Creative Commons Attribution (CC BY) license (https://creativecommons.org/licenses/by/4.0/).

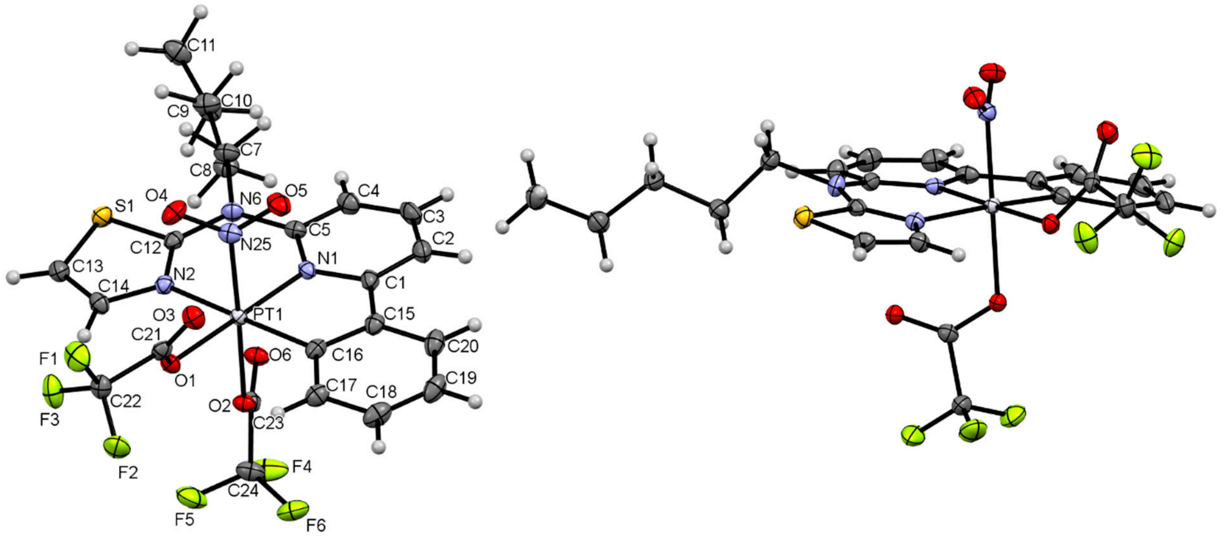

Figure S32. Molecular structure in the single crystal of **[Pt(TFA)$_2$NO$_2$(L)]** (left), viewed along the luminophore-Pt plane (right). Displacement ellipsoids are shown at 50 % probability.

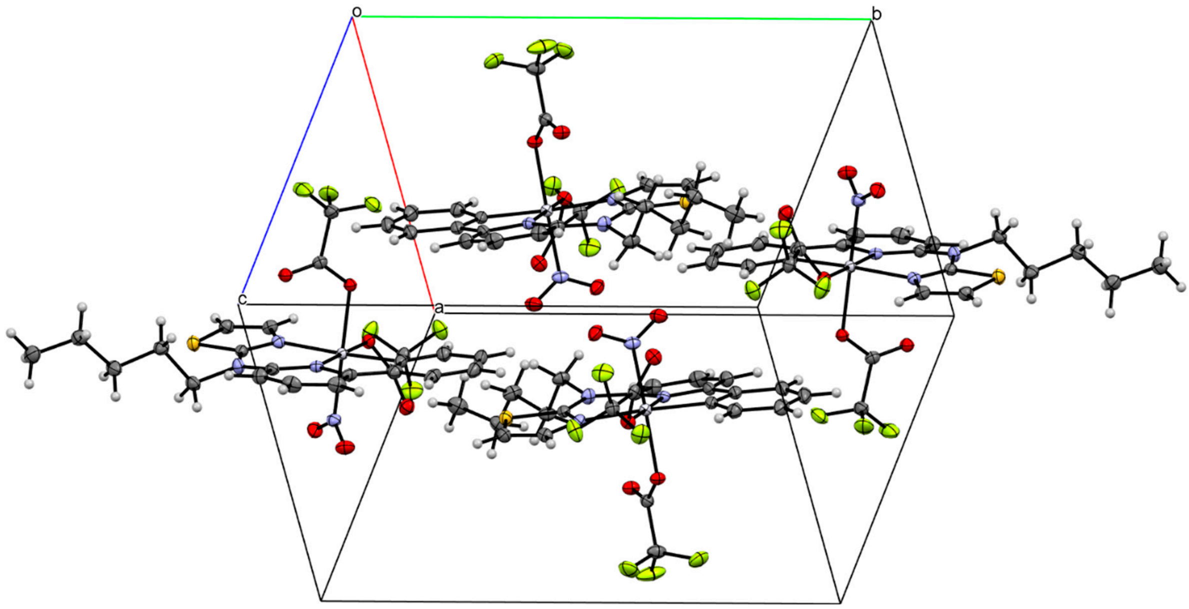

Figure S33. Unit cell for the crystal structure of **[Pt(TFA)$_2$NO$_2$(L)]**. Displacement ellipsoids are shown at 50 % probability.

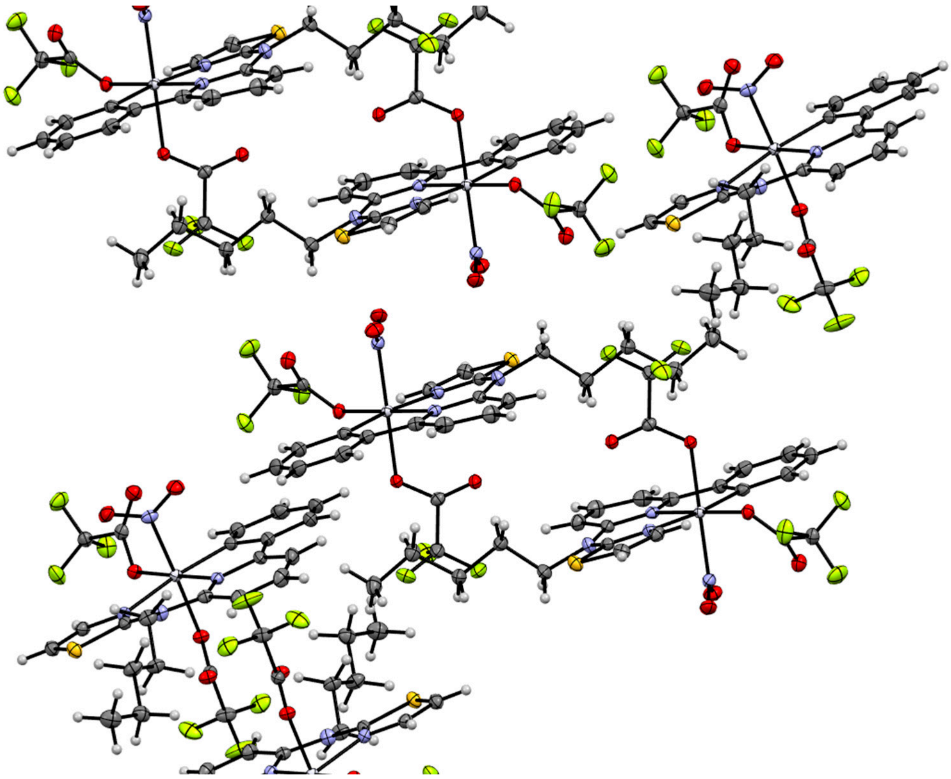

Figure S34. Dimer-formation and F/O-H-interactions (right) of [**Pt(TFA)$_2$NO$_2$(L)**]. Displacement ellipsoids are shown at 50 % probability.

Table S4. Selected bond lengths and angles for [**Pt(TFA)$_2$NO$_2$(L)**].

X-Y	*d*(X-Y) in Å	X-Y-Z	∠(XYZ) in °
Pt1-N1	2.004(3)	N1-Pt1-N2	91.82(11)
Pt1-N2	2.084(4)	N2-Pt1-C16	173.50(13)
Pt1-C16	1.997(4)	C16-Pt1-O1	96.19(13)
Pt1-N25	2.014(3)	O1-Pt1-N1	179.16(10)
Pt1-O1	2.017(3)	N1-Pt1-C16	83.21(14)
Pt1-O2	2.072(3)	N2-Pt1-O1	88.81(10)
		C16-Pt1-N25	94.55(14)
		N2-Pt1-N25	89.28(12)
		N1-Pt1-N25	86.02(14)
		O1-Pt1-N25	93.44(13)
		C16-Pt1-O2	86.62(12)
		N2-Pt1-O2	89.68(11)
		N1-Pt1-O2	95.44(13)
		O1-Pt1-O2	85.11(12)
		N25-Pt1-O2	178.23(11)

Structure of [PtTFA(L)] (ViS14_p-1; CCDC-Nr.: 2298208):

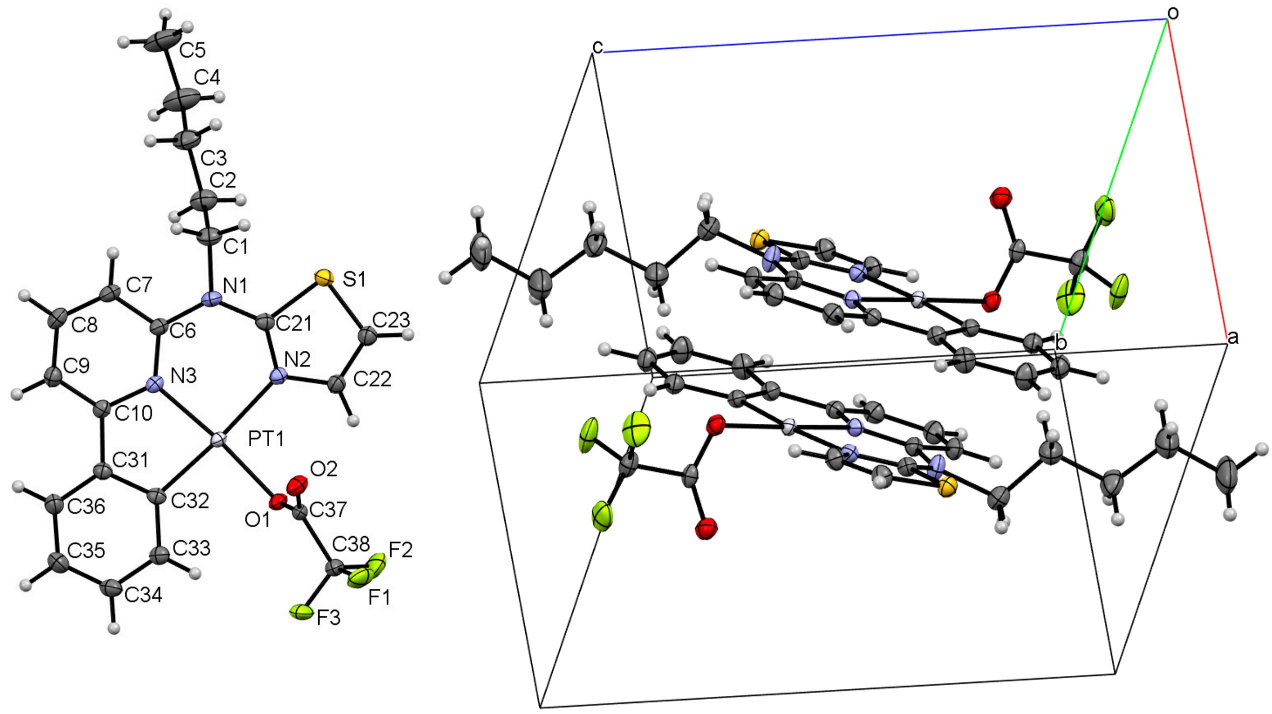

Figure S35. Molecular structure in the single crystal of **[PtTFA(L)]** (left), unit cell for the crystal structure (right). Displacement ellipsoids are shown at 50 % probability.

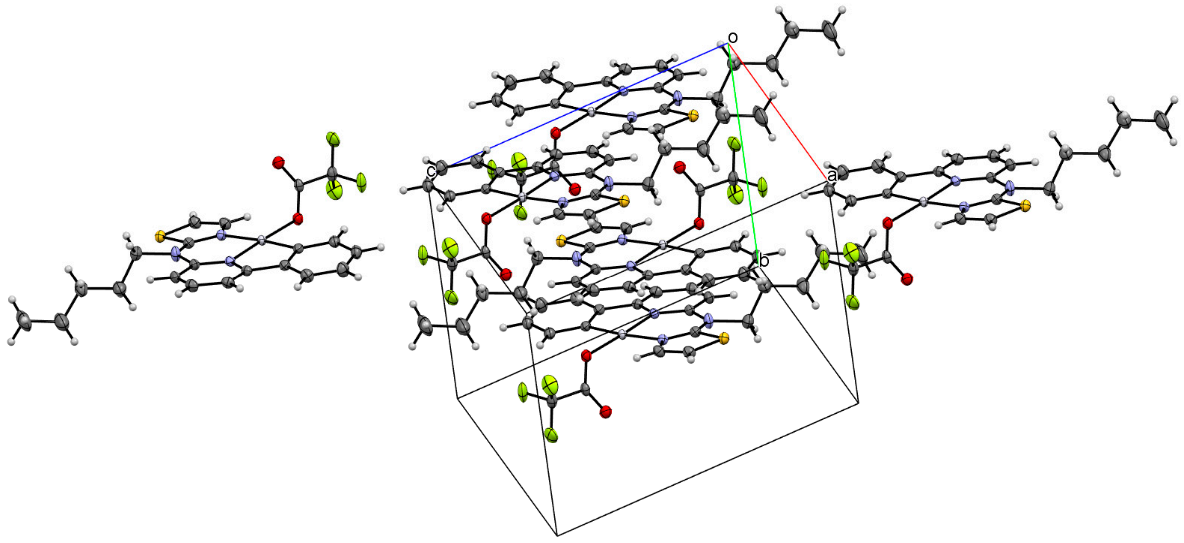

Figure S36. Dimer-formation and F/O-H-interactions of **[PtTFA(L)]**. (Displacement ellipsoids are shown at 50 % probability.

Table S5. Selected bond lengths and angles for **[PtTFA(L)]**.

X-Y	*d*(X-Y) in Å	X-Y-Z	∠(XYZ) in °
Pt1-N2	2.057 (2)	N2-Pt1-N3	92.33 (8)
Pt1-N3	1.991 (2)	N3-Pt1-C32	83.24 (9)
Pt1-C32	1.977 (2)	C32-Pt1-O1	91.54 (9)
Pt1-O1	2.0364 (17)	O1-Pt1-N2	92.91 (8)
		N2-Pt1-C32	175.15(8)
		N3-Pt1-O1	174.73 (7)

Structure of [$PtCl_3$(L)] (ve17_sadp21c_a; CCDC-Nr.: 2298210):

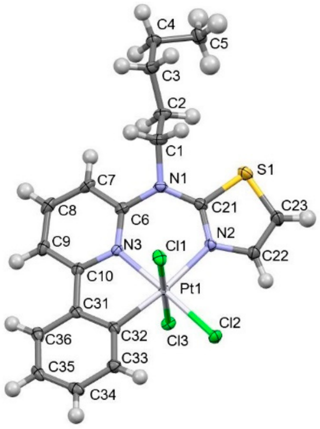

Figure S37. Molecular structure in the single crystal of [**$PtNO_2$(L)**] (left), viewed along the luminophore-Pt plane (right). Displacement ellipsoids are shown at 50 % probability.

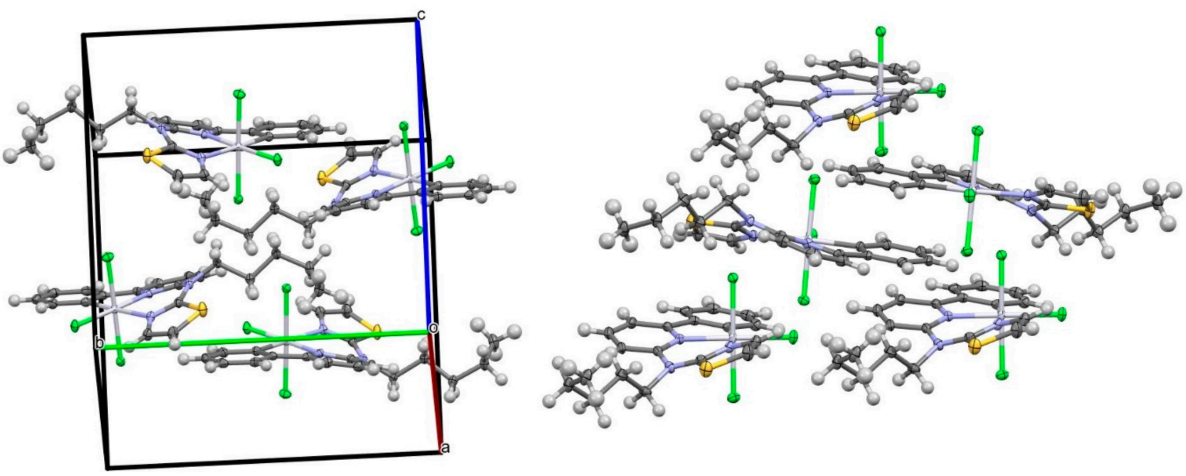

Figure S38. The unit cell for the crystal structure of [**$PtCl_3$(L)**] (left). Display of π-π-stacking and Cl-H-interactions (right). Displacement ellipsoids are shown at 50 % probability.

Table S6. Selected bond lengths and angles for [**$PtCl_3$(L)**].

X-Y	*d*(X-Y) in Å	X-Y-Z	∠(XYZ) in °
Pt1-N2	2.124(2)	N2-Pt1-N3	89.53(10)
Pt1-N3	2.032(2)	N3-Pt1-C32	82.51(11)
Pt1-C32	2.003(3)	C32-Pt1-Cl2	95.49(9)
Pt1-Cl1	2.3182(7)	Cl2-Pt1-N2	92.51(7)
Pt1-Cl2	2.3133(7)	N2-Pt1-C32	171.57(11)
Pt1-Cl3	2.3287(7)	N3-Pt1-Cl2	177.89(7)
		C32-Pt1-Cl1	91.41(8)
		N3-Pt1-Cl1	89.02(7)
		N2-Pt1-Cl1	91.23(7)
		Cl2-Pt1-Cl1	90.36(3)
		C32-Pt1-Cl3	87.91(8)
		N3-Pt1-Cl3	91.70(7)
		N2-Pt1-Cl3	89.55(7)
		Cl2-Pt1-Cl3	88.90(3)
		Cl1-Pt1-Cl3	178.94(2)

II. Photophysical characterization:

Absorption spectra were measured with a Shimadzu UV-1900i UV-VIS-NIR spectrophotometer.

Steady-state excitation and emission spectra were recorded on a FluoTime 300 spectrometer from PicoQuant equipped with a 300 W ozone-free Xe lamp (250–900 nm), a 10 W Xe flashlamp (250–900 nm, pulse width ca. 1 µs) with repetition rates of 0.1–300 Hz, a double-grating excitation monochromator (Czerny-Turner type, grating with 1200 lines/mm, blaze wavelength: 300 nm), diode lasers (pulse width < 80 ps) operated by a computer-controlled laser driver PDL-828 "Sepia II" (repetition rate up to 80 MHz, burst mode for slow and weak decays), two double-grating emission monochromators (Czerny-Turner, selectable gratings blazed at 500 nm with 2.7 nm/mm dispersion and 1200 lines/mm, or blazed at 1200 nm with 5.4 nm/mm dispersion and 600 lines/mm) with adjustable slit width between 25 µm and 7 mm, Glan-Thompson polarizers for excitation (after the Xe-lamps) and emission (after the sample).

Diverse sample holders and a Peltier-cooled mounting unit ranging from -15 to 110 °C, along with two detectors (namely a PMA Hybrid-07 from PicoQuant with transit time spread FWHM < 50 ps, 200–850 nm, or a H10330C-45-C3 NIR detector with transit time spread FWHM 0.4 ns, 950–1700 nm from Hamamatsu) were used. Steady-state spectra and photoluminescence lifetimes were recorded in TCSPC mode by a PicoHarp 300 (minimum base resolution 4 ps) or in MCS mode by a TimeHarp 260 (where up to several ms can be traced). Emission and excitation spectra were corrected for source intensity (lamp and grating) by standard correction curves. Lifetime analysis was performed using the commercial EasyTau 2 software (PicoQuant). The quality of the fit was assessed by minimizing the reduced chi-squared function (χ2) and visual inspection of the weighted residuals and their autocorrelation. All solvents used were of spectrometric grade (Uvasol®, Merck). Photoluminescence quantum yields were measured with a Hamamatsu Photonics absolute PL quantum yield measurement system (C9920-02) equipped with a L9799-01 CW Xe light source (150 W), a monochromator, a C7473 photonic multi-channel analyzer, an integrating sphere and employing U6039-05 software (Hamamatsu Photonics, Ltd., Shizuoka, Japan).

Table S7. Full set of photoluminescence data including Φ_L, as well as exited state lifetimes (τ_{av}) for each complex in DCM at 298 K and in frozen glassy matrix of DCM/MeOH (V:V = 1:1) at 77 K. For multiexponential decays, the amplitude-weighted average lifetimes are given as well as the different components in square brackets with relative amplitudes as percentages in parentheses.

Complex	Medium (T/K)	λ_{em}	λ_{exc}	τ_{av}	Φ_L ± 0.02/± 0.05
**[PtCO(L)]**	DCM, air (298)	495, 531, 568sh	368, 306	1.369 ± 0.006	< 0.02
	DCM, Ar (298)			6.79 ± 0.04 [3 ± 2 (79); 5 ± 2 (21)]	< 0.02
	Glassy matrix (77)	481, 517, 550	394, 357	37.9 ± 0.2 [55 ± 4 (42); 29 ± 6 (48); 11 ± 5 (10)]	n.d.
**[PtNO$_2$(L)]**	DCM, air (298)	488, 524, 555sh	400sh, 365, 335	0.517 ± 0.002 [0.591 ± 0.001 (87); 0.023 ± 0.003 (13)]	< 0.02
	DCM, Ar (298)			1.044 ± 0.005 [2.5 ± 0.6 (2); 1.01 ± 0.02 (98)]	< 0.02
	Glassy matrix (77)	483, 520, 550sh	390sh, 360	60.56 ± 0.07 [75.2 ± 0.3 (65); 33.5 ± 0.4 (35)]	n.d.
**[PtTFA(L)]**	DCM, air (298)	493, 527, 562sh	403sh, 363	0.547 ± 0.001	< 0.02
	DCM, Ar (298)			1.087 ± 0.001	0.03
	Glassy matrix (77)	485, 520, 555	390sh, 365	37.60 ± 0.07 [48.1 ± 0.1 (52); 26 ± 1 (48)]	n.d.
**[PtCl$_3$(L)]**	DCM, air (298)	n.d.	n.d.	n.d.	n.d.

	DCM, Ar (298)			n.d.	n.d.
	Glassy matrix (77)	484, 519, 555	370, 349, 312, 286	23.6 ± 0.2[30 ± 3 (53); 17 ± 2 (47)]	n.d.

II.1 Steady-state and time-resolved photoluminescence spectroscopy

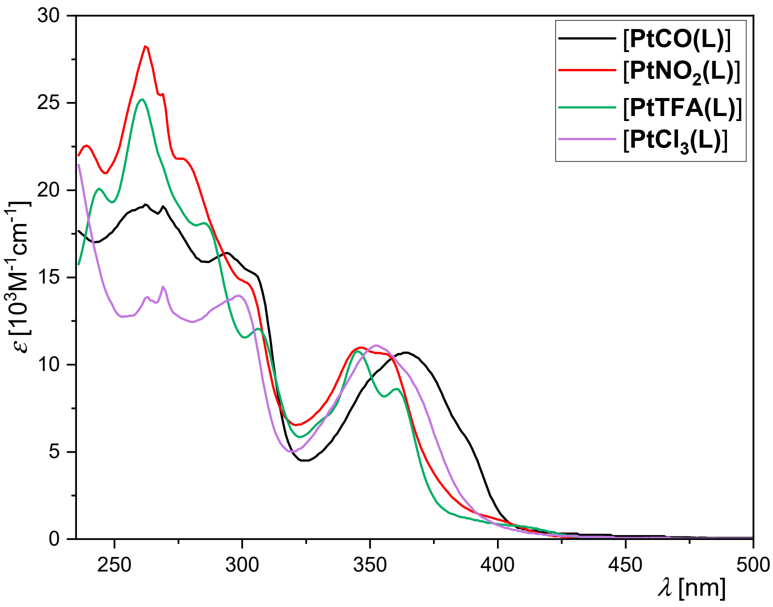

Figure S39. Molar absorption coefficient of **[PtCO(L)]** (black), **[PtNO$_2$(L)]** (red), **[PtTFA(L)]** (green), and **[PtCl$_3$(L)]** (violett) (validity range: c = 2×10^{-5}–1×10^{-6} M in DCM at 298 K).

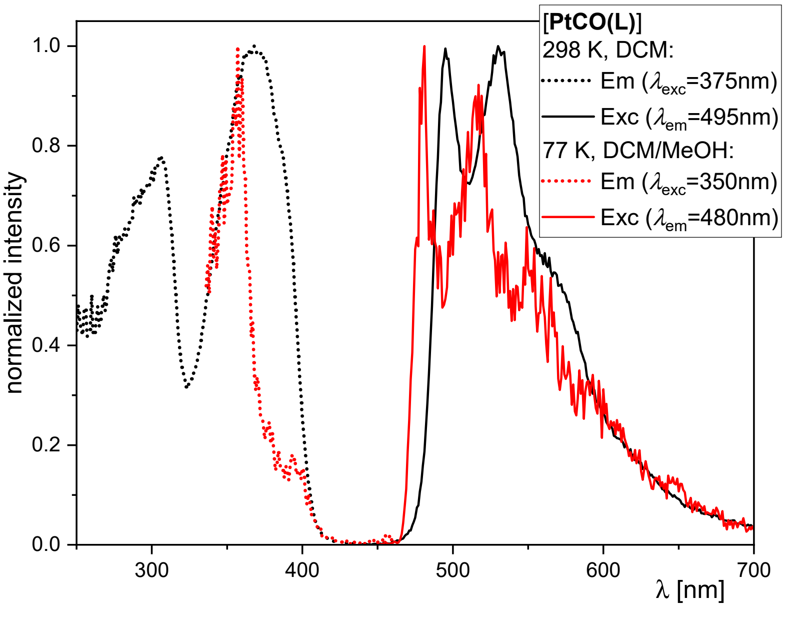

Figure S40. Excitation (dotted) and emission spectra (bold) of **[PtCO(L)]** at 298 K (black, λ_{exc} = 375 nm, λ_{em} = 495 nm) in liquid DCM and at 77 K (red, λ_{exc} = 350 nm, λ_{em} = 480 nm) in a frozen glassy matrix of DCM/MeOH (V:V = 1:1). All solutions were optically diluted (A < 0.1). Normalized to the highest intensity.

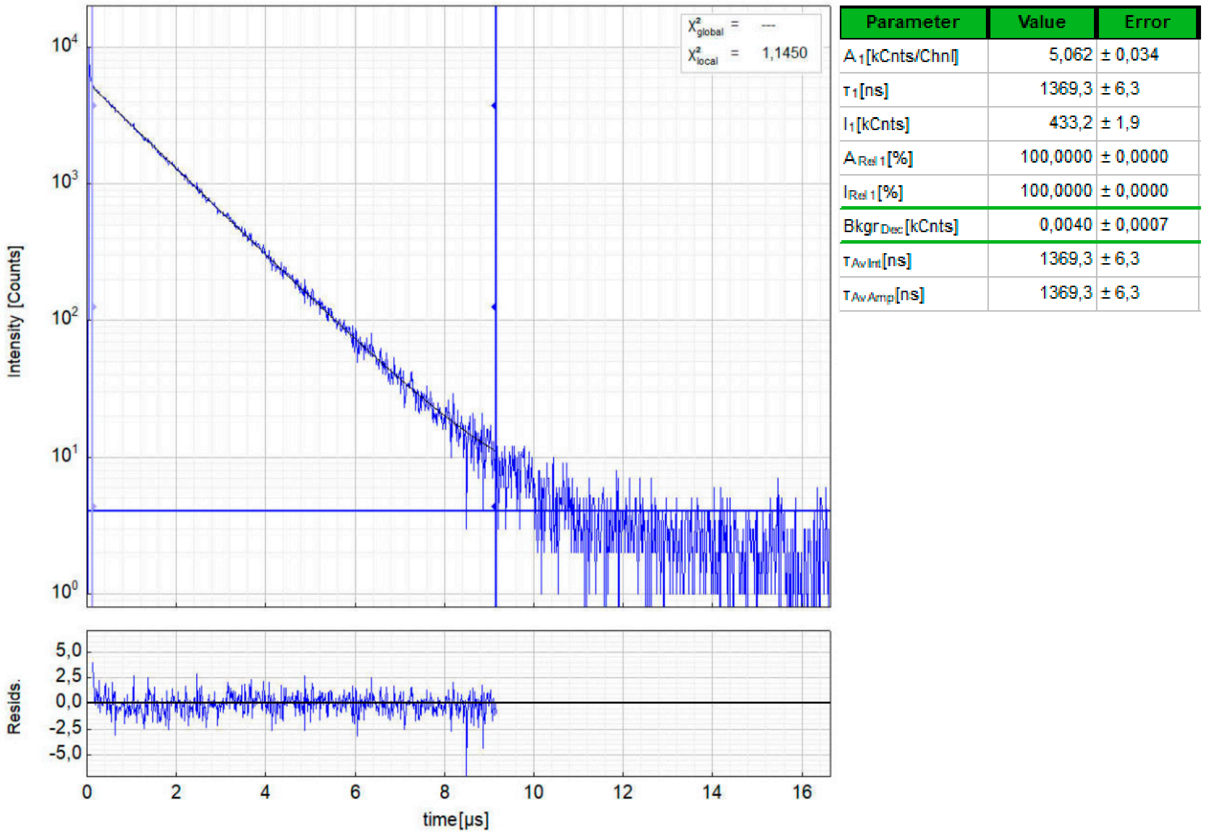

Parameter	Value	Error
A1[kCnts/Chnl]	5,062	± 0,034
τ1[ns]	1369,3	± 6,3
I1[kCnts]	433,2	± 1,9
ARel 1[%]	100,0000	± 0,0000
IRel 1[%]	100,0000	± 0,0000
BkgrDec[kCnts]	0,0040	± 0,0007
τAv Int[ns]	1369,3	± 6,3
τAv Amp[ns]	1369,3	± 6,3

Figure S41. Left: Time-resolved photoluminescence decay of **[PtCO(L)]** in liquid air-equilibrated DCM at 298 K, including the residuals (λ_{exc} = 376.7 nm, λ_{em} = 495 nm). Right: Fitting parameters including pre-exponential factors and confidence limits.

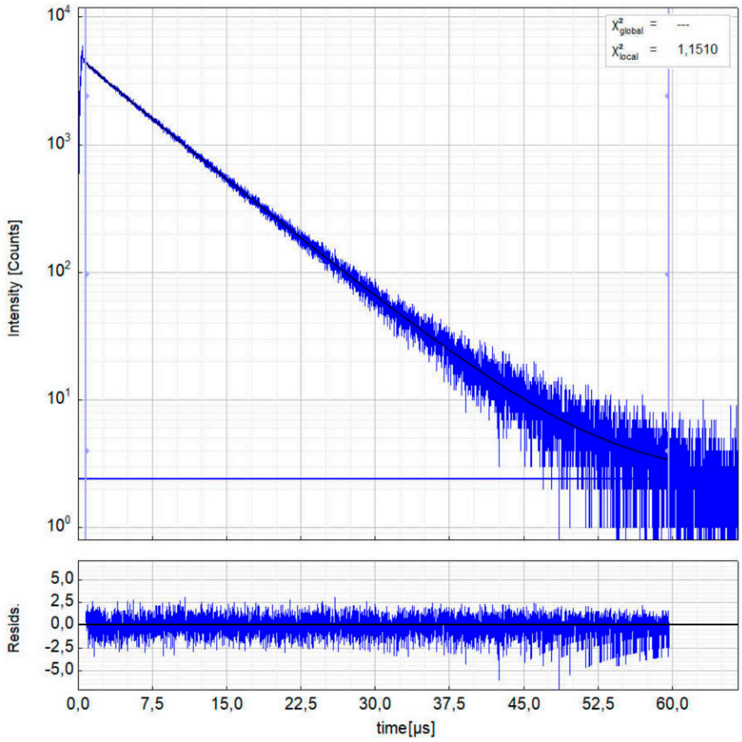

Parameter	Value	Error
A1[kCnts/Chnl]	3,4	± 2,2
τ1[ns]	7250	± 990
I1[kCnts]	3099	± 1823
ARel 1[%]	79	± 51
IRel 1[%]	84	± 50
A2[kCnts/Chnl]	0,9	± 2,2
τ2[ns]	5055	± 2182
I2[kCnts]	569	± 1825
ARel 2[%]	21	± 51
IRel 2[%]	16	± 50
BkgrDec[kCnts]	0,0024	± 0,0004
τAv Int[ns]	6909	± 21
τAv Amp[ns]	6792	± 44

Figure S42. Left: Time-resolved photoluminescence decay of **[PtCO(L)]** in liquid Ar-purged DCM at 298 K, including the residuals (λ_{exc} = 376.7 nm, λ_{em} = 495 nm). Right: Fitting parameters including pre-exponential factors and confidence limits.

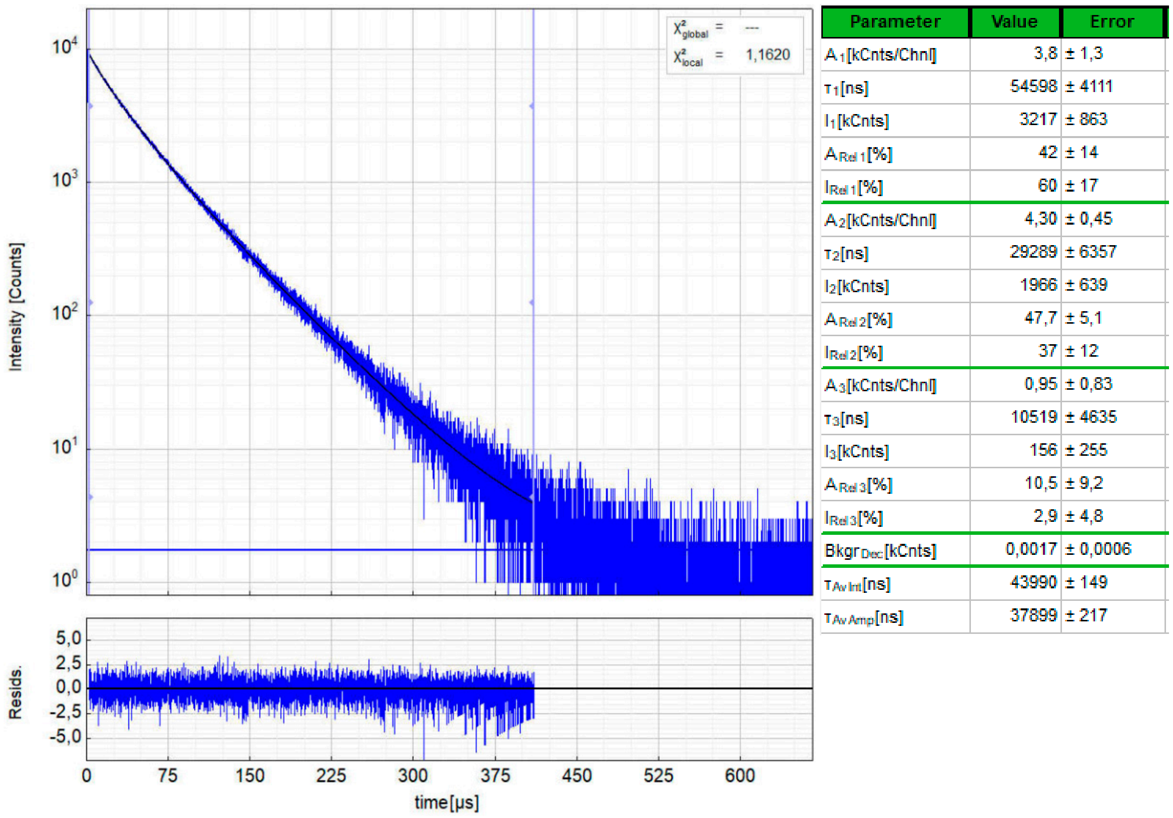

Parameter	Value	Error
A_1[kCnts/Chnl]	3,8	± 1,3
τ_1[ns]	54598	± 4111
I_1[kCnts]	3217	± 863
$A_{Rel\,1}$[%]	42	± 14
$I_{Rel\,1}$[%]	60	± 17
A_2[kCnts/Chnl]	4,30	± 0,45
τ_2[ns]	29289	± 6357
I_2[kCnts]	1966	± 639
$A_{Rel\,2}$[%]	47,7	± 5,1
$I_{Rel\,2}$[%]	37	± 12
A_3[kCnts/Chnl]	0,95	± 0,83
τ_3[ns]	10519	± 4635
I_3[kCnts]	156	± 255
$A_{Rel\,3}$[%]	10,5	± 9,2
$I_{Rel\,3}$[%]	2,9	± 4,8
$Bkgr_{Dec}$[kCnts]	0,0017	± 0,0006
$\tau_{Av\,Int}$[ns]	43990	± 149
$\tau_{Av\,Amp}$[ns]	37899	± 217

Figure S43. Left: Time-resolved photoluminescence decay of [**PtCO(L)**] in a frozen glassy matrix of DCM/MeOH (V:V = 1:1) at 77 K, including the residuals (λ_{exc} = 376.7 nm, λ_{em} = 480 nm). Right: Fitting parameters including pre-exponential factors and confidence limits.

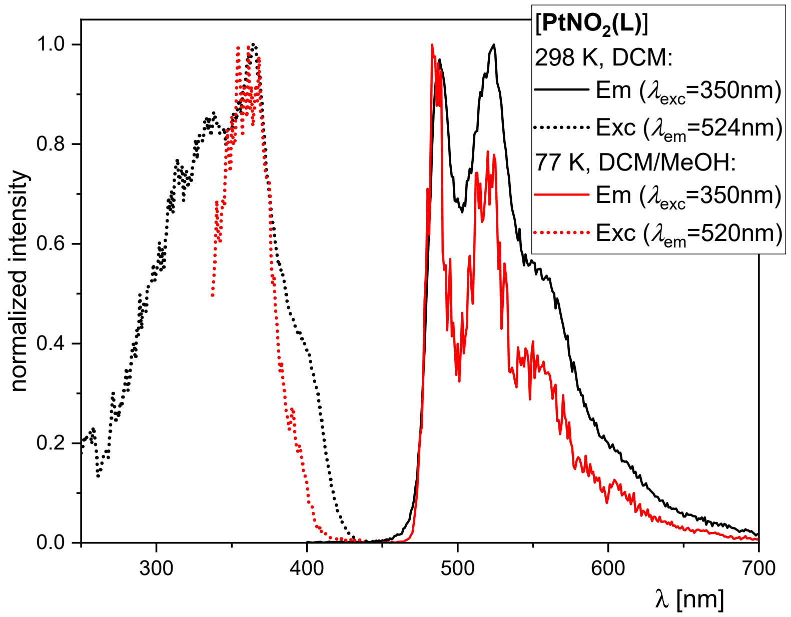

Figure S44. Excitation (dotted) and emission spectra (bold) of [**PtNO$_2$(L)**] at 298 K (black, λ_{exc} = 350 nm, λ_{em} = 524 nm) in liquid DCM and at 77 K (red, λ_{exc} = 350 nm, λ_{em} = 520 nm) in a frozen glassy matrix of DCM/MeOH (V:V = 1:1). All solutions were optically diluted (A < 0.1). Normalized to the highest intensity.

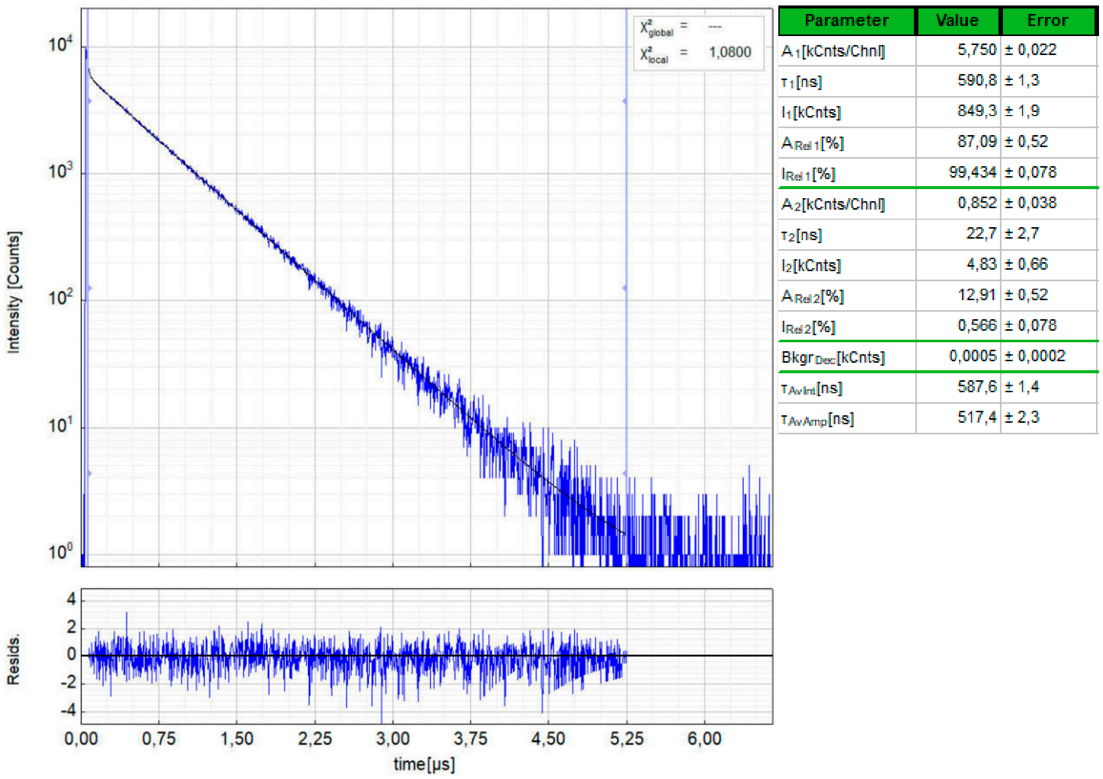

Parameter	Value	Error
A_1[kCnts/Chnl]	5,750	± 0,022
τ_1[ns]	590,8	± 1,3
I_1[kCnts]	849,3	± 1,9
$A_{Rel\,1}$[%]	87,09	± 0,52
$I_{Rel\,1}$[%]	99,434	± 0,078
A_2[kCnts/Chnl]	0,852	± 0,038
τ_2[ns]	22,7	± 2,7
I_2[kCnts]	4,83	± 0,66
$A_{Rel\,2}$[%]	12,91	± 0,52
$I_{Rel\,2}$[%]	0,566	± 0,078
$Bkgr_{Dec}$[kCnts]	0,0005	± 0,0002
$\tau_{Av\,Int}$[ns]	587,6	± 1,4
$\tau_{Av\,Amp}$[ns]	517,4	± 2,3

Figure S45. Left: Time-resolved photoluminescence decay of [**PtNO2(L)**] in liquid air-equilibrated DCM at 298 K, including the residuals (λ_{exc} = 376.7 nm, λ_{em} = 524 nm). Right: Fitting parameters including pre-exponential factors and confidence limits.

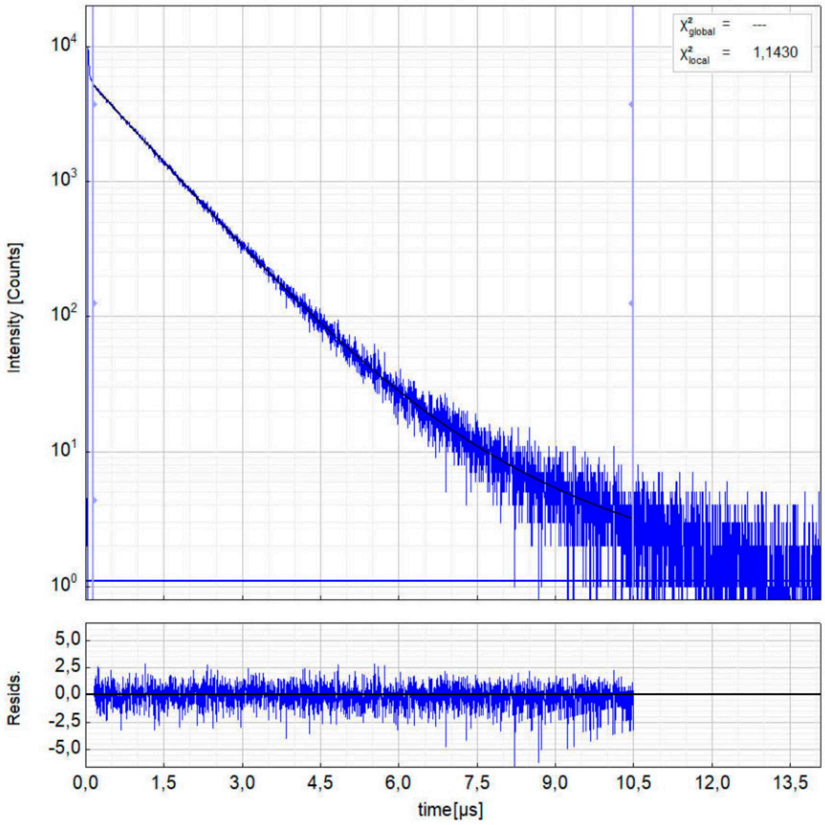

Parameter	Value	Error	Plot
A_1[kCnts/Chnl]	0.130	± 0.051	☐
τ_1[ns]	2453	± 615	☐
I_1[kCnts]	80	± 18	☐
$A_{Rel\,1}$[%]	2.54	± 0.98	☐
$I_{Rel\,1}$[%]	6.0	± 1.4	☐
A_2[kCnts/Chnl]	4.994	± 0.048	☐
τ_2[ns]	1007.0	± 9.4	☐
I_2[kCnts]	1257	± 22	☐
$A_{Rel\,2}$[%]	97.46	± 0.98	☐
$I_{Rel\,2}$[%]	94.0	± 1.4	☐
$Bkgr_{Dec}$[kCnts]	0.0011	± 0.0012	☐
$\tau_{Av\,Int}$[ns]	1093	± 18	☐
$\tau_{Av\,Amp}$[ns]	1043.7	± 4.8	☐

Figure S46. Left: Time-resolved photoluminescence decay of [**PtNO2(L)**] in liquid Ar-purged DCM at 298 K, including the residuals (λ_{exc} = 376.7 nm, λ_{em} = 524 nm). Right: Fitting parameters including pre-exponential factors and confidence limits.

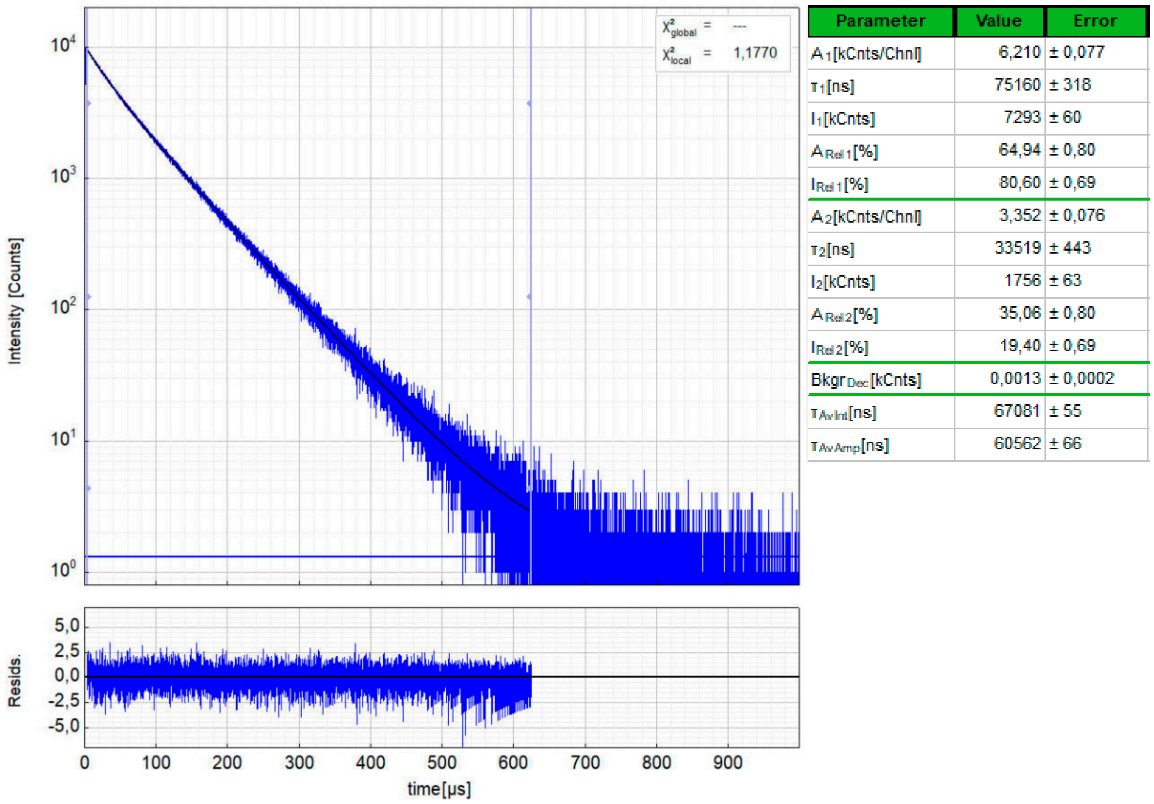

Parameter	Value	Error
A_1[kCnts/Chnl]	6,210	± 0,077
τ_1[ns]	75160	± 318
I_1[kCnts]	7293	± 60
$A_{Rel\,1}$[%]	64,94	± 0,80
$I_{Rel\,1}$[%]	80,60	± 0,69
A_2[kCnts/Chnl]	3,352	± 0,076
τ_2[ns]	33519	± 443
I_2[kCnts]	1756	± 63
$A_{Rel\,2}$[%]	35,06	± 0,80
$I_{Rel\,2}$[%]	19,40	± 0,69
$Bkgr_{Dec}$[kCnts]	0,0013	± 0,0002
$\tau_{Av\,Int}$[ns]	67081	± 55
$\tau_{Av\,Amp}$[ns]	60562	± 66

Figure S47. Left: Time-resolved photoluminescence decay of [**PtNO2(L)**] in a frozen glassy matrix of DCM/MeOH (V:V = 1:1) at 77 K, including the residuals (λ_{exc} = 376.7 nm, λ_{em} = 520 nm). Right: Fitting parameters including pre-exponential factors and confidence limits.

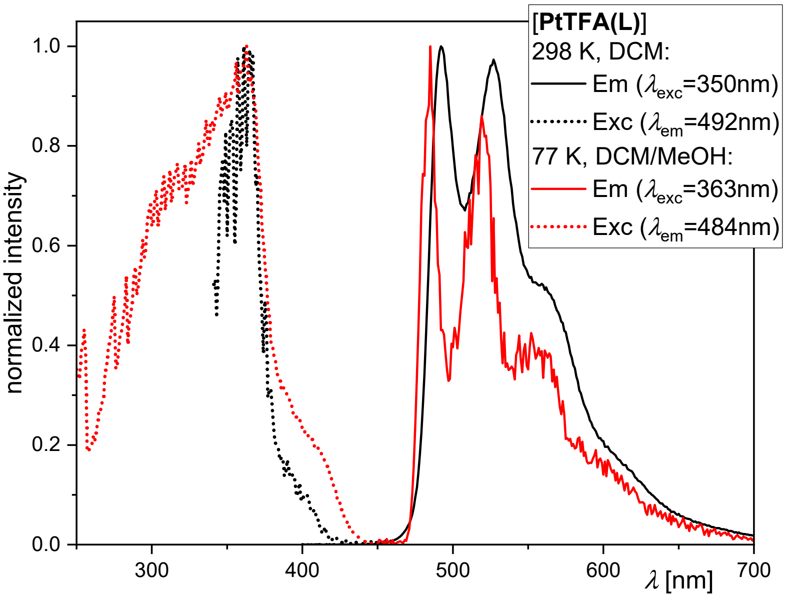

Figure S48. Excitation (dotted) and emission spectra (bold) of [**PtTFA(L)**] at 298 K (black, λ_{exc} = 350 nm, λ_{em} = 492 nm) in liquid DCM and at 77 K (red, λ_{exc} = 363 nm, λ_{em} = 484 nm) in a frozen glassy matrix of DCM/MeOH (V:V = 1:1). All solutions were optically diluted (A < 0.1). Normalized to the highest intensity.

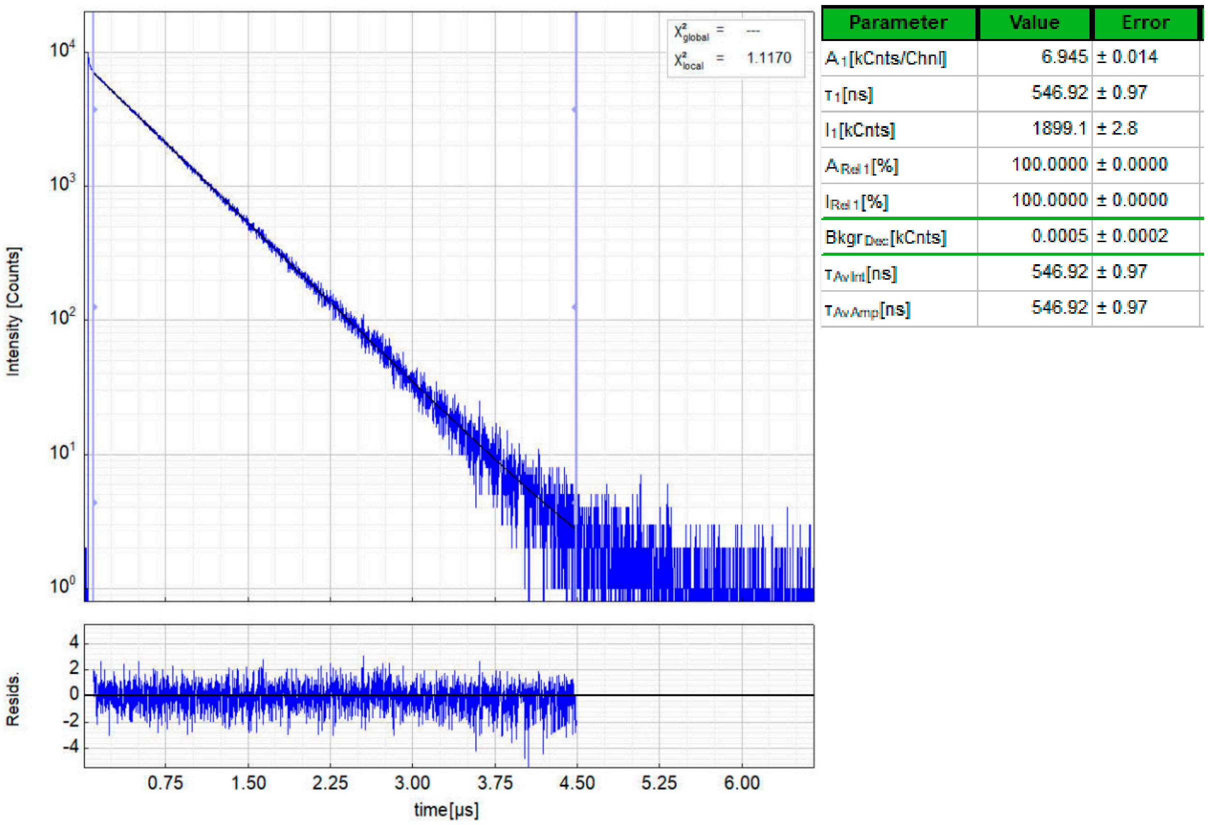

Parameter	Value	Error
A_1[kCnts/Chnl]	6.945	± 0.014
τ_1[ns]	546.92	± 0.97
I_1[kCnts]	1899.1	± 2.8
$A_{Rel\,1}$[%]	100.0000	± 0.0000
$I_{Rel\,1}$[%]	100.0000	± 0.0000
$Bkgr_{Dec}$[kCnts]	0.0005	± 0.0002
$\tau_{Av\,Int}$[ns]	546.92	± 0.97
$\tau_{Av\,Amp}$[ns]	546.92	± 0.97

Figure S49. Left: Time-resolved photoluminescence decay of [**PtTFA(L)**] in liquid air-equilibrated DCM at 298 K, including the residuals (λ_{exc} = 376.7 nm, λ_{em} = 492 nm). Right: Fitting parameters including pre-exponential factors and confidence limits.

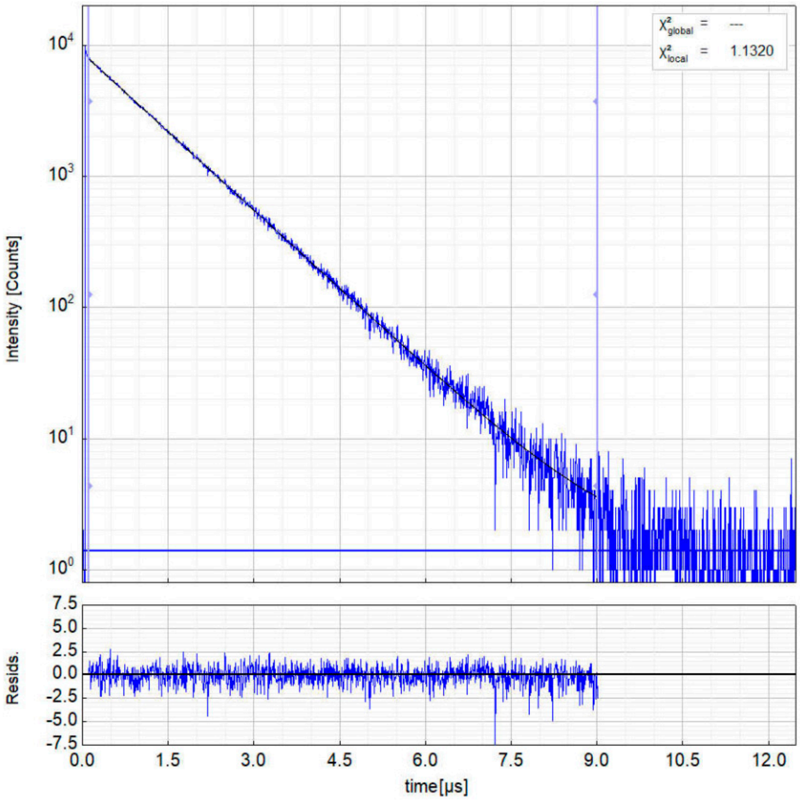

Parameter	Value	Error
A_1[kCnts/Chnl]	7.903	± 0.022
τ_1[ns]	1086.5	± 2.3
I_1[kCnts]	1073.4	± 1.4
$A_{Rel\,1}$[%]	100.0000	± 0.0000
$I_{Rel\,1}$[%]	100.0000	± 0.0000
$Bkgr_{Dec}$[kCnts]	0.0014	± 0.0005
$\tau_{Av\,Int}$[ns]	1086.5	± 2.3
$\tau_{Av\,Amp}$[ns]	1086.5	± 2.3

Figure S50. Left: Time-resolved photoluminescence decay of [**PtTFA(L)**] in liquid Ar-purged DCM at 298 K, including the residuals (λ_{exc} = 376.7 nm, λ_{em} = 492 nm). Right: Fitting parameters including pre-exponential factors and confidence limits.

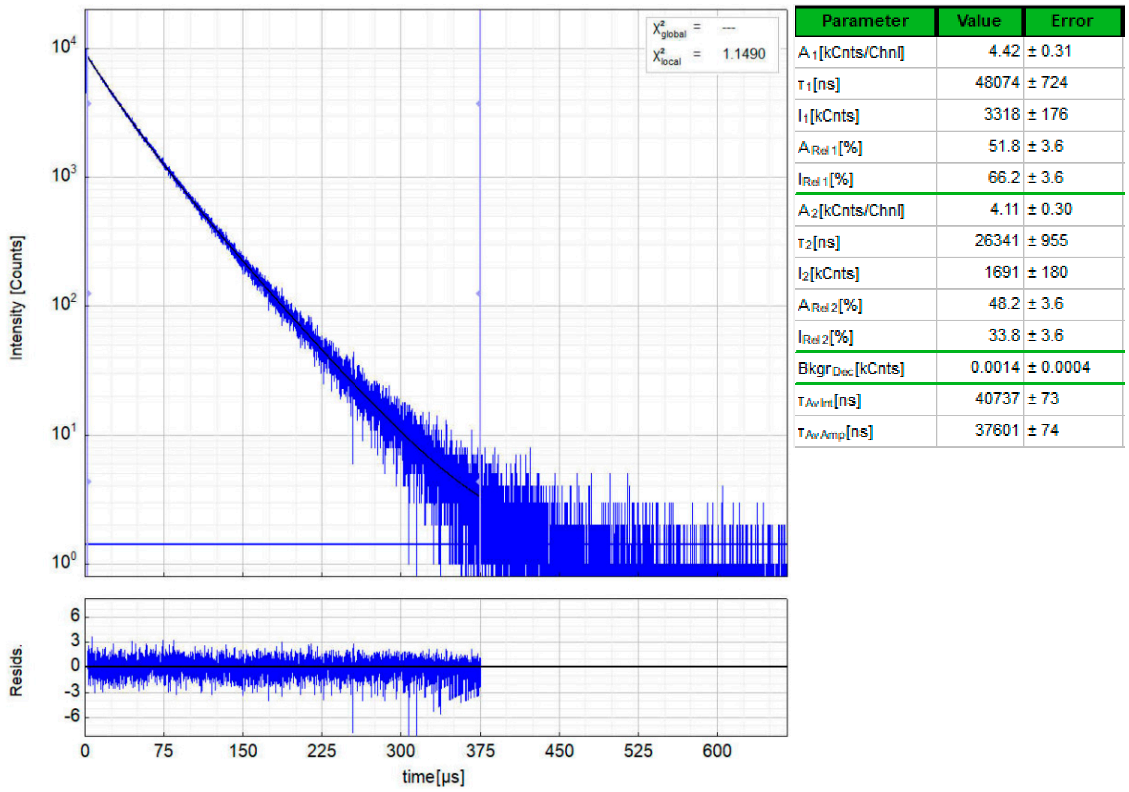

Parameter	Value	Error
A_1[kCnts/Chnl]	4.42	± 0.31
τ_1[ns]	48074	± 724
I_1[kCnts]	3318	± 176
$A_{Rel\,1}$[%]	51.8	± 3.6
$I_{Rel\,1}$[%]	66.2	± 3.6
A_2[kCnts/Chnl]	4.11	± 0.30
τ_2[ns]	26341	± 955
I_2[kCnts]	1691	± 180
$A_{Rel\,2}$[%]	48.2	± 3.6
$I_{Rel\,2}$[%]	33.8	± 3.6
$Bkgr_{Dec}$[kCnts]	0.0014	± 0.0004
$\tau_{Av\,Int}$[ns]	40737	± 73
$\tau_{Av\,Amp}$[ns]	37601	± 74

Figure S51. Left: Time-resolved photoluminescence decay of [**PtTFA(L)**] in a frozen glassy matrix of DCM/MeOH (V:V = 1:1) at 77 K, including the residuals (λ_{exc} = 376.7 nm, λ_{em} = 484 nm). Right: Fitting parameters including pre-exponential factors and confidence limits.

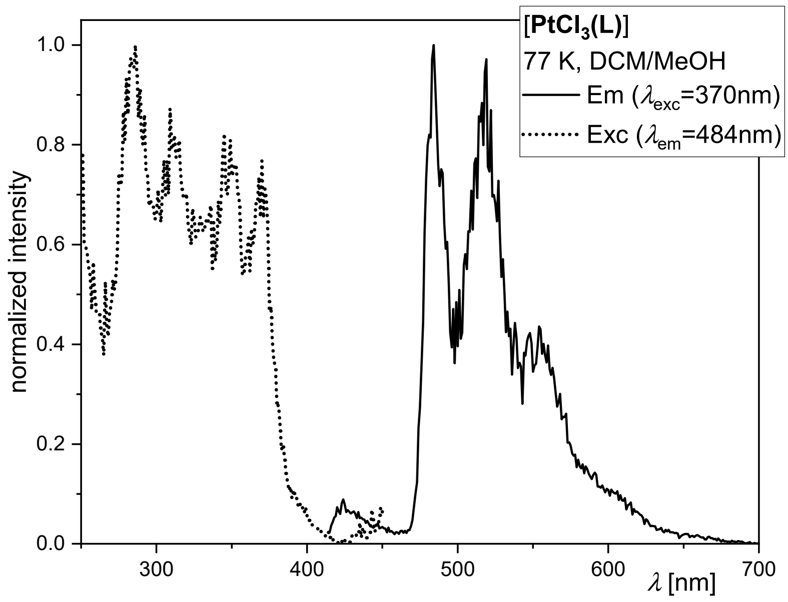

Figure S52. Excitation (dotted) and emission spectra (bold) of [**$PtCl_3$(L)**] at 77 K (black, λ_{exc} = 370 nm, λ_{em} = 484 nm) in a frozen glassy matrix of DCM/MeOH (V:V = 1:1). All solutions were optically diluted (A < 0.1). Normalized to the highest intensity.

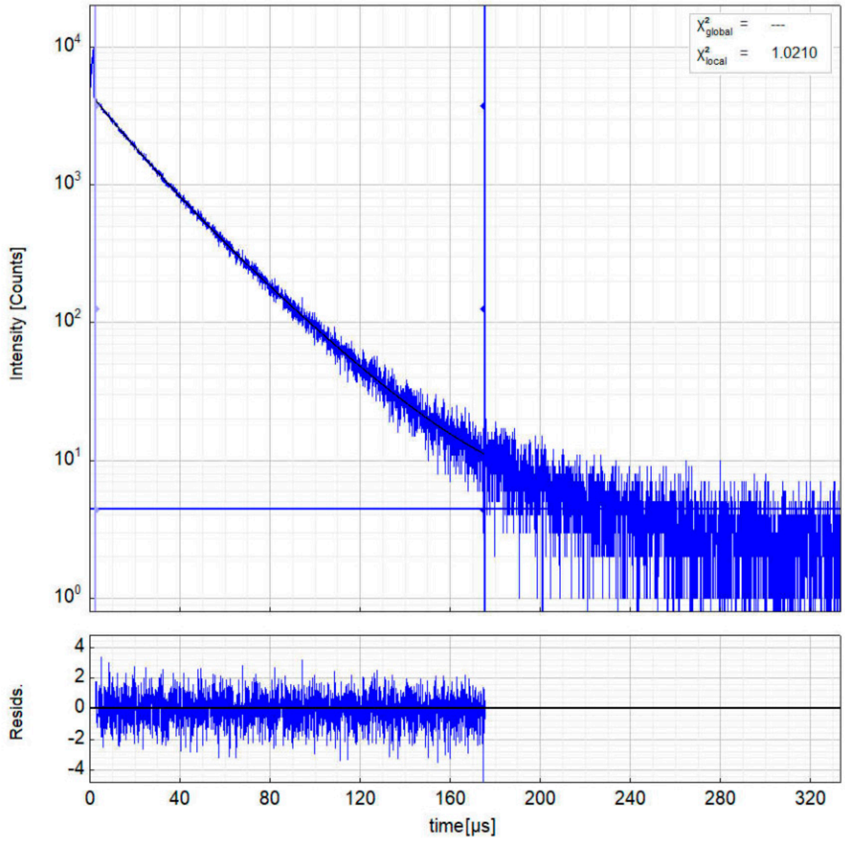

Parameter	Value	Error
A_1[kCnts/Chnl]	2.17	± 0.61
τ_1[ns]	29791	± 2527
I_1[kCnts]	1011	± 223
$A_{Rel\,1}$[%]	53	± 15
$I_{Rel\,1}$[%]	67	± 15
A_2[kCnts/Chnl]	1.92	± 0.61
τ_2[ns]	16619	± 1962
I_2[kCnts]	498	± 226
$A_{Rel\,2}$[%]	47	± 15
$I_{Rel\,2}$[%]	33	± 15
$Bkgr_{Dec}$[kCnts]	0.0045	± 0.0016
$\tau_{Av\,Int}$[ns]	25441	± 271
$\tau_{Av\,Amp}$[ns]	23611	± 179

Figure S53. Left: Time-resolved photoluminescence decay of [**$PtCl_3$(L)**] in a frozen glassy matrix of DCM/MeOH (V:V = 1:1) at 77 K, including the residuals (λ_{exc} = 376.7 nm, λ_{em} = 484 nm). Right: Fitting parameters including pre-exponential factors and confidence limits.

III. Cyclovoltammetry measurements

The electrochemical experiments were carried out using a three-electrode configuration with the Potentiostat VersaSTAT 3 (AMETEK, Berwyn Pennsylvania). For the voltammetric experiments, a glassy carbon disk (0.0707 cm^2) polished on a felt pad with alumina (0.05 µm) was used as a working electrode. A silver wire was used as a pseudo-reference electrode and a platinum coil as a counter electrode. All measurements were performed in Ar-purged DCM solutions containing tetrabutylammonium hexafluoridophosphate (TBAHFP, purity >99%, Merck, 0.1 M) as support electrolyte and the corresponding complex (1mM). Ferrocene/ferrocenium redox couple (Fc/Fc^+ = 0.40 V vs. SCE) was used as an internal standard and all reported potential values are expressed relative to these.

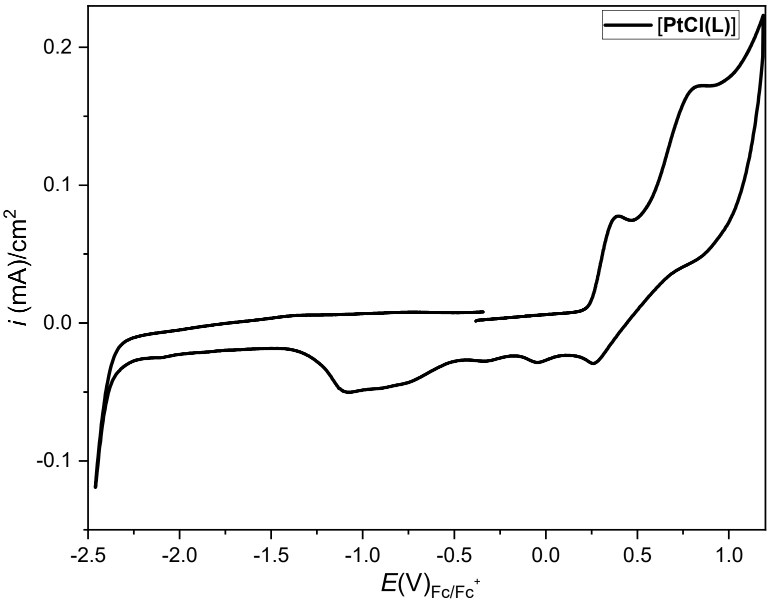

Figure S54. Cyclic voltammograms of [**PtCl(L)**] in 0.1 M n-Bu_4NPF_6 liquid Ar-purged DCM.

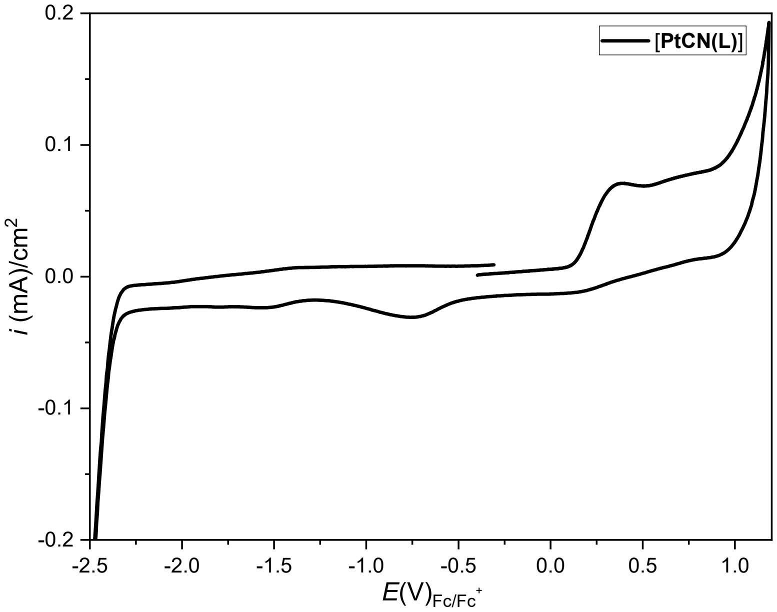

Figure S55. Cyclic voltammograms of [**PtCN(L)**] in 0.1 M n-Bu_4NPF_6 liquid Ar-purged DCM.

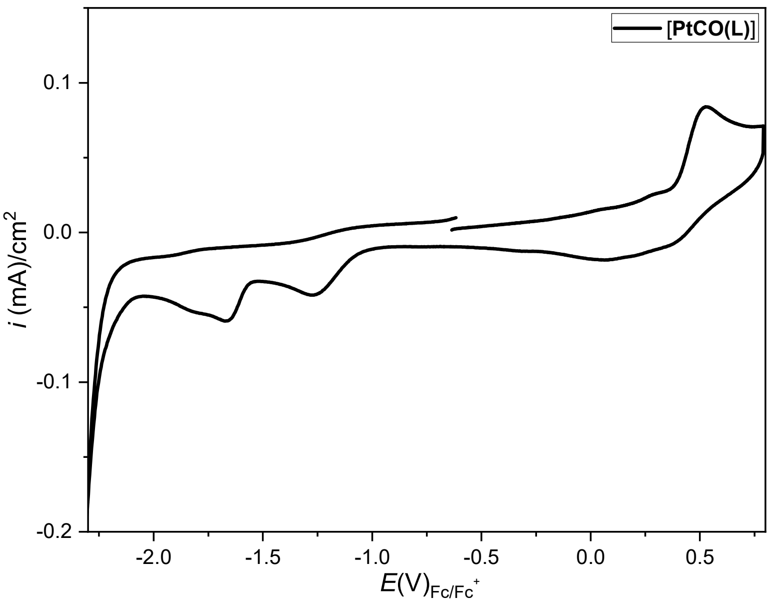

Figure S56. Cyclic voltammograms of [**PtCO(L)**] in 0.1 M n-Bu_4NPF_6 liquid Ar-purged DCM.

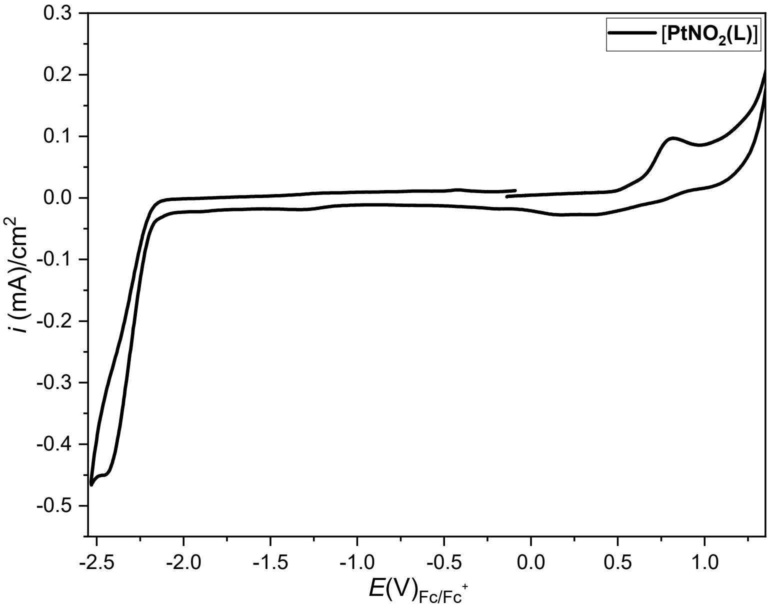

Figure S57. Cyclic voltammograms of [**$PtNO_2$(L)**] in 0.1 M n-Bu_4NPF_6 liquid Ar-purged DCM.

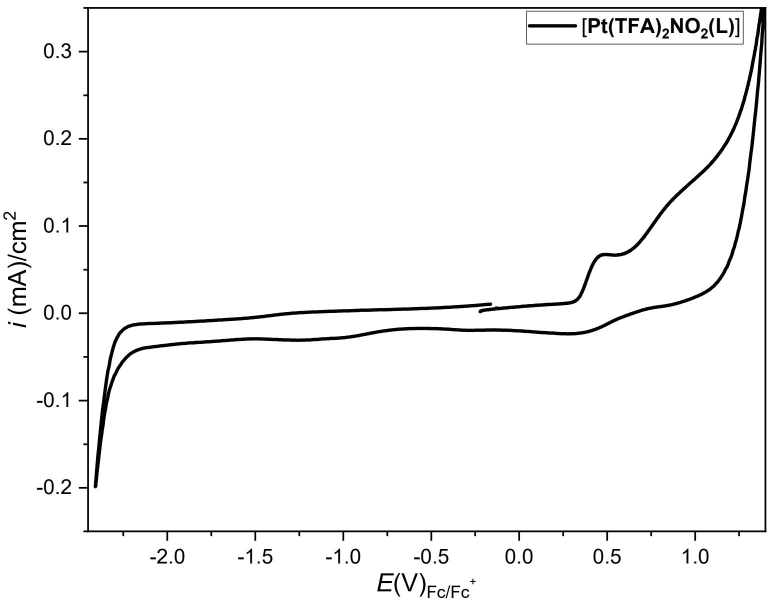

Figure S58. Cyclic voltammograms of [**Pt(TFA)**$_2$**NO**$_2$**(L)**] in 0.1 M n-Bu_4NPF_6 liquid Ar-purged DCM.

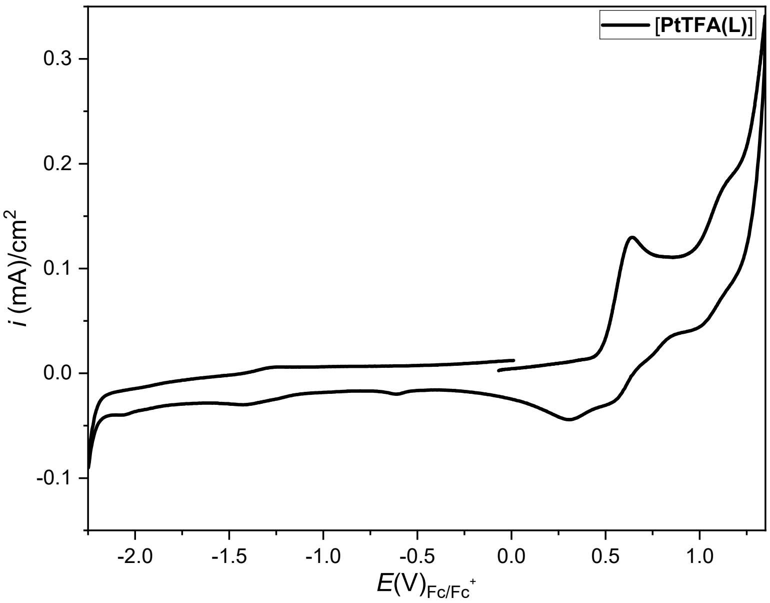

Figure S59. Cyclic voltammograms of [**PtTFA(L)**] in 0.1 M n-Bu_4NPF_6 liquid Ar-purged DCM.

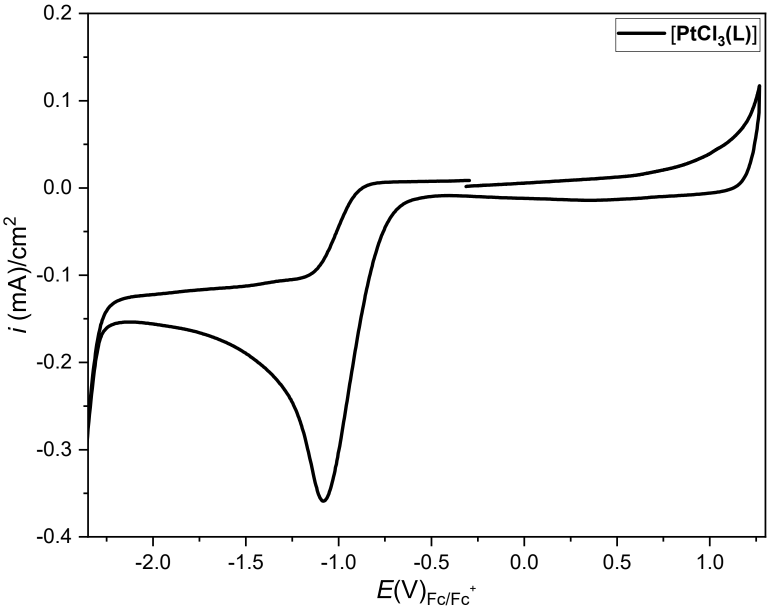

Figure S60. Cyclic voltammograms of [**PtCl$_3$(L)**] in 0.1 M n-Bu_4NPF_6 liquid Ar-purged DCM.

References

[42] The herein reported chlorido- and cyanido-derivatives bearing *n*-pentyl moieties were synthesized analogously to the corresponding *n*-propyl-substituted Pt(II) complexes described previously and in further detail by Knedel, T.-O.; Buss, S.; Maisuls, I.; Daniliuc, C.G.; Schlüsener, C.; Brandt, P.; Weingart, O.; Vollrath, A.; Janiak, C.; Strassert, C.A. Encapsulation of Phosphorescent Pt(II) Complexes in Zn-Based Metal–Organic Frameworks toward Oxygen-Sensing Porous Materials. *Inorg. Chem.* **2020**, *59*, 7252–7264. https://doi.org/10.1021/acs.inorgchem.0c00678; see also ref. [56].

[74] Ikemoto, K.; Inokuma, Y.; Rissanen, K.; Fujita, M. X-Ray Snapshot Observation of Palladium-Mediated Aromatic Bromination in a Porous Complex. *J. Am. Chem. Soc.* **2014**, *136* (19), 6892–6895. DOI: 10.1021/ja502996h.

Disclaimer/Publisher's Note: The statements, opinions and data contained in all publications are solely those of the individual author(s) and contributor(s) and not of MDPI and/or the editor(s). MDPI and/or the editor(s) disclaim responsibility for any injury to people or property resulting from any ideas, methods, instructions or products referred to in the content.
